# The hypertrophic cardiomyopathy-associated A331P actin variant enhances basal contractile activity and elicits resting muscle dysfunction

**DOI:** 10.1016/j.isci.2025.111816

**Published:** 2025-01-16

**Authors:** Matthew H. Doran, Michael J. Rynkiewicz, Evan Despond, Meera C. Viswanathan, Aditi Madan, Kripa Chitre, Axel J. Fenwick, Duncan Sousa, William Lehman, John F. Dawson, Anthony Cammarato

**Affiliations:** 1 Department of Pharmacology, Physiology & Biophysics, Boston University Chobanian & Avedisian School of Medicine, 72 E. Concord St, Boston, MA 02118, USA; 2 Department Molecular and Cellular Biology, University of Guelph, 50 Stone Road E, Guelph, ON N1G 2W1, Canada; 3 Department of Medicine, Division of Cardiology, Johns Hopkins University, 720 Rutland Avenue, Baltimore, MD 21205, USA; 4 Department of Biophysics, Johns Hopkins University, 725 N. Wolfe Street, Baltimore, MD 21205, USA; 5 Department of Physiology, Johns Hopkins University School of Medicine, 720 Rutland Avenue, Baltimore, MD 21205, USA

**Keywords:** Biological sciences, Cell biology, Molecular interaction, Molecular Structure, Structural biology

## Abstract

Previous studies aimed at defining the mechanistic basis of hypertrophic cardiomyopathy caused by A331P cardiac actin have reported conflicting results. The mutation is located along an actin surface strand, proximal to residues that interact with tropomyosin. These F-actin-tropomyosin associations are vital for proper contractile inhibition. To help resolve disease pathogenesis, we implemented a multidisciplinary approach. Transgenic *Drosophila*, expressing A331P actin, displayed skeletal muscle hypercontraction and elevated basal myocardial activity. A331P thin filaments, reconstituted using recombinant human cardiac actin, exhibited higher *in vitro* myosin-based sliding speeds, exclusively at low Ca^2+^ concentrations. Cryo-EM-based reconstructions revealed no detectable A331P-related structural perturbations in F-actin. *In silico*, however, the P331-containing actin surface strand was less mobile and established diminished van der Waal’s attractive forces with tropomyosin, which correlated with greater variability in inhibitory tropomyosin positioning. Such mutation-induced effects potentially elevate resting contractile activity among our models and may stimulate pathology in patients.

## Highlights

• A331P HCM actin caused hypercontraction and diastolic dysfunction in flies
• Cryo-EM of human wild-type and A331P actin revealed minimal structural differences
• MD resolved variability in inhibitory tropomyosin positioning along A331P filaments
• Elevated resting contractile activity alone may be sufficient to drive pathology

## Graphical abstract

**Figure:**
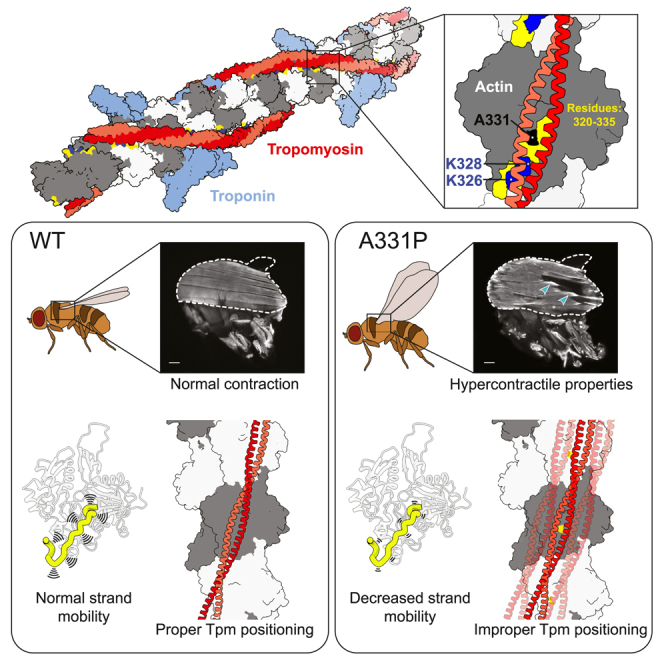

## Introduction

Myocardial force production is derived from cyclical interactions between myosin-containing thick and actin-containing thin filaments. It is regulated by mechanisms coupled to both sets of myofilaments.^1^ These mechanisms ensure rapid activation at the onset of systole and, importantly, precise inhibition of contraction at its termination. Thin filament-based regulation relies on troponin-tropomyosin (Tn-Tpm) complexes that span the length of filamentous actin (F-actin).^1^^,^^2^^,^^3^ During diastole, the inhibitory subunit of Ca^2+^-free Tn (i.e., TnI) binds to F-actin, and constrains Tpm to the B-state where it physically blocks and limits myosin attachment.^4^^,^^5^^,^^6^ Upon activation, intracellular Ca^2+^ increases and binds to Tn, resulting in an ∼10 Å azimuthal pivot of Tpm on F-actin to the C-state position. This easing of steric inhibition facilitates initial, weak binding of myosin S1 heads to the partially exposed actin-binding interface. A transition from weak-to-strong S1 cross-bridge binding further displaces Tpm to the M-state, which promotes additional S1 binding and contraction.^7^ Relaxation commences as sarcoplasmic Ca^2+^ levels decline and Ca^2+^ dissociates from Tn.^8^ Acto-myosin crossbridge cycling and force subsequently wane as F-actin attachment sites become increasingly occluded while Tpm returns to its inhibitory B-state configuration upon TnI-F-actin rebinding.

Striated muscle Tpm is a dimeric, alpha-helical, coiled coil protein consisting predominantly of seven tandem pseudo-repeating modules that bind seven successive actin protomers along F-actin.^9^^,^^10^ The dimers self-polymerize, end to end, to form a continuous strand that wraps around the F-actin helix of the thin filament. While the association of Tpm with F-actin is highly dependent upon electrostatic bonds, both short-range van der Waals (vdW) and hydrophobic forces also contribute to the total interaction energy.^9^^,^^10^^,^^11^^,^^12^^,^^13^^,^^14^ The sum of these non-bonded interactions precludes the dissociation of polymerized Tpm cables from F-actin while, nevertheless, still enabling their translocation back-and-forth over the thin filament surface.^15^^,^^16^ Changes in these non-bonded F-actin-Tpm interactions likely modulate Tpm translocation and thin filament regulation, alter contractility at the cellular and organ levels, and stimulate disease.^13^^,^^16^^,^^17^^,^^18^^,^^19^^,^^20^

Hypertrophic cardiomyopathy (HCM) is a heterogeneous disease of the heart, with a prevalence estimated at 1 in 200.^21^ Mutations in cardiac thin filament subunits, including Troponin T (TnT), TnI, Tpm and actin account for ∼5%, to possibly upwards of 30%, of all cases, with the preponderance of these lesions clustering at subunit interfaces.^22^^,^^23^ HCM is characterized by asymmetric myocardial growth, thickening of the interventricular septum and concomitant chamber reduction, disrupted Ca^2+^ homeostasis, hyperdynamic systolic contraction, and diastolic dysfunction.^21^^,^^23^^,^^24^^,^^25^ Experimentally, pathogenic mutations that increase the magnitude and/or duration of myocardial tension during systolic twitches have been shown to correlate strongly with concentric hypertrophy.^26^^,^^27^ These observations are consistent with the earliest signs and reported drivers of HCM, including hypercontraction and impaired relaxation^24^^,^^25^ which, from the perspective of the thin filament, can result from inadequate myosin blocking, implicating a compromised B-state as one potential pathological trigger.

**Figure 1:**
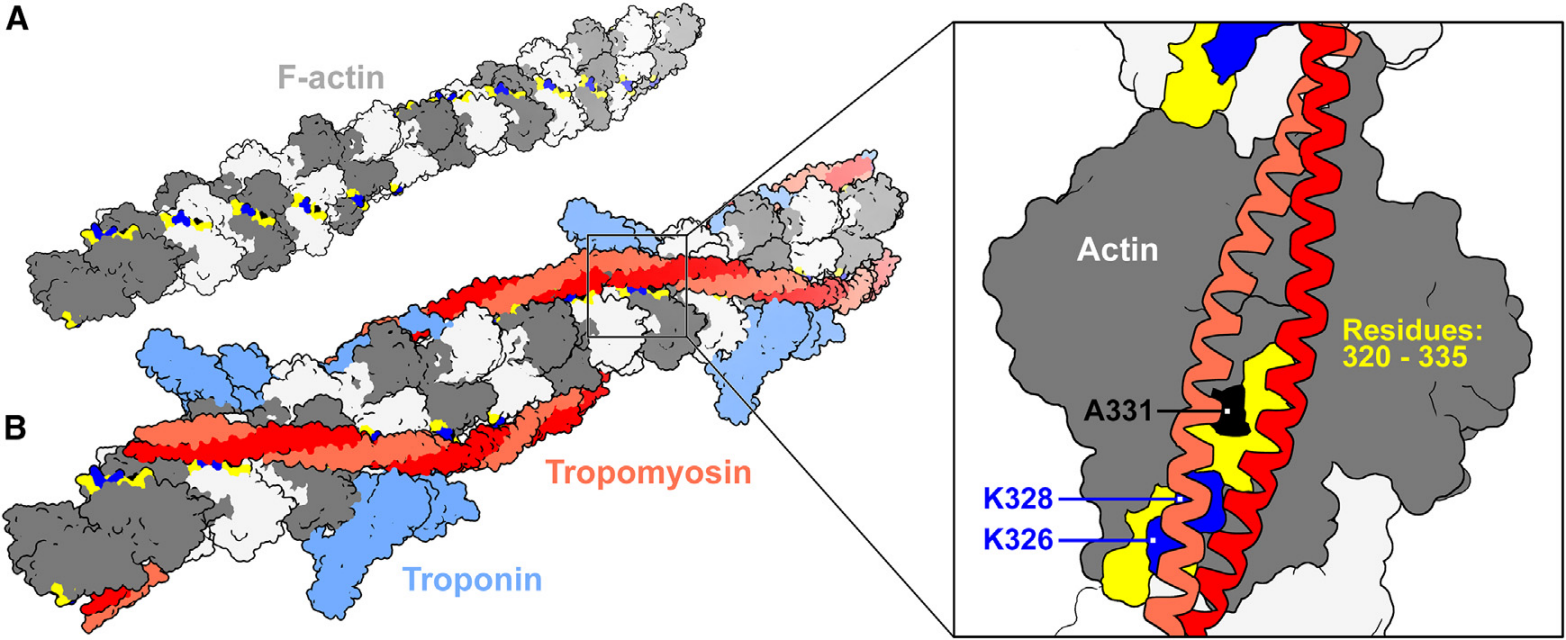
The ACTC A331P HCM mutation lies proximal to residues that form critical salt bridges with Tpm (A) F-actin in the absence of thin filament regulatory proteins. Individual actin protomers are presented in different shades of gray to help visualize helical packing. The 320–334 surface strand of each actin protomer is displayed in yellow. The loci of K326/328 are shown in blue, and A331 in black, along the surface strand. (B) Thin filament, replete with Tn (light blue) and Tpm (red/salmon) regulatory components. Inset: a single actin subunit depicting Tpm in its inhibitory, B-state position. K326/328 (blue), located along actin’s 320–334 surface strand (yellow), establish highly favorable, stabilizing electrostatic contacts with Tpm.^6^^,^^9^^,^^19^^,^^20^^,^^28^^,^^30^ These recurring salt bridges, formed between each actin protomer and every pseudo-repeat binding module along Tpm’s entire length, are required for proper B-state positioning and muscle relaxation. A331 is displayed in black. Models are based on PDB 6KN8, and rendered in ChimeraX.^34^

In addition to the well-described, canonical TnI-F-actin binding requirement, recent advances have identified additional protein-protein associations that are also essential for inhibitory Tpm positioning and establishing the B-state. These include newly predicted contacts between the N-terminus of TnT and actin, and highly favorable F-actin-Tpm electrostatic bonds.^4^^,^^6^^,^^9^^,^^19^^,^^20^^,^^28^^,^^29^^,^^30^^,^^31^^,^^32^ The latter primarily involve a series of repetitive ionic contacts between K326 and K328 of each actin protomer and every pseudo-repeat module along Tpm (Figure 1). Notably, we have established that these associations facilitate Tn-Tpm-mediated steric blocking of S1 *in silico*, *in vitro*, and *in vivo* and, therefore, contribute to proper relaxation of striated muscle.^16^^,^^19^^,^^20^^,^^28^^,^^33^ Moreover, we have shown that the formation of these critical interfacial electrostatic contacts is likely influenced by the physico-chemical nature of the entire K326/328-containing surface strand (i.e., actin residues 320–334).^20^ In fact, we found that changes in flexibility of the strand distorted the F-actin-Tpm electrostatic energy landscape and correlated with aberrant contractile inhibition and excessive force. These data suggest that a certain degree of strand mobility is necessary to facilitate surface complementarity, proper intermolecular binding, and B-state formation.

The cardiac actin (ACTC) A331P HCM mutation, initially identified ∼25 years ago,^35^ lies proximal to K326 and K328 along the 320–334 surface strand (Figure 1), and therefore may impact its role in Tpm positioning. Previous studies found that A331P ACTC mouse models were indistinguishable from control, while data generated with reconstituted bovine trabeculae suggested that the variant reduced maximum tension and prompted hypocontractility, results at odds with those found for other HCM mutations.^24^^,^^25^^,^^36^^,^^37^ These inconsistencies muddle our understanding of the pathological basis of disease. Here, we present a study that comprehensively assesses the effects of A331P actin on striated muscle, across multiple length scales and degrees of organizational complexity, from the organ through the molecular level.

Given its proximity to actin residues that participate in Tpm inhibitory positioning, we hypothesized that the mutation disrupts the Tpm B-state and acto-myosin inhibition at rest. A331P actin provoked hypercontraction in *Drosophila melanogaster* skeletal and cardiac muscle, with the myocardial effects resulting from excessive, poorly impeded, Ca^2+^-independent crossbridge cycling, during diastole. Thin filaments, reconstituted using recombinant human A331P ACTC and vertebrate Tn-Tpm displayed significantly higher *in vitro* sliding speeds exclusively under low Ca^2+^, relative to controls. While cryo-EM-based reconstructions of human wild-type and A331P ACTC filaments were virtually indistinguishable, molecular dynamics (MD) simulations, based on these models, revealed a mutation-induced decrease in protomer flexibility, particularly along the 320–334, A331P-containing surface strand. Additionally, F-actin-Tpm interaction energies, computed from simulations of filaments containing Tpm and Tn-regulatory elements, were diminished in the mutant filament. This decrease was largely attributable to weakened F-actin-Tpm vdW interactions, and was associated with a greater degree of B-state Tpm positional variability while the C-state remained mostly unperturbed. These results are consistent with a possible role for vdW forces in steric inhibition that may be disrupted due to a relatively rigid mutant 320–334 surface strand. A poorly maintained B-state could account for the impaired muscle relaxation and elevated basal tension observed among our models. Mechanistically, our findings point to an A331P ACTC-mediated gain-in-sarcomeric-function, predominantly at rest that, even in the face of potentially diminished systolic force, may be sufficient to drive hypertrophic remodeling and disease.

## Results

### A331P actin causes skeletal muscle hypercontraction in *Drosophila*

**Figure 2:**
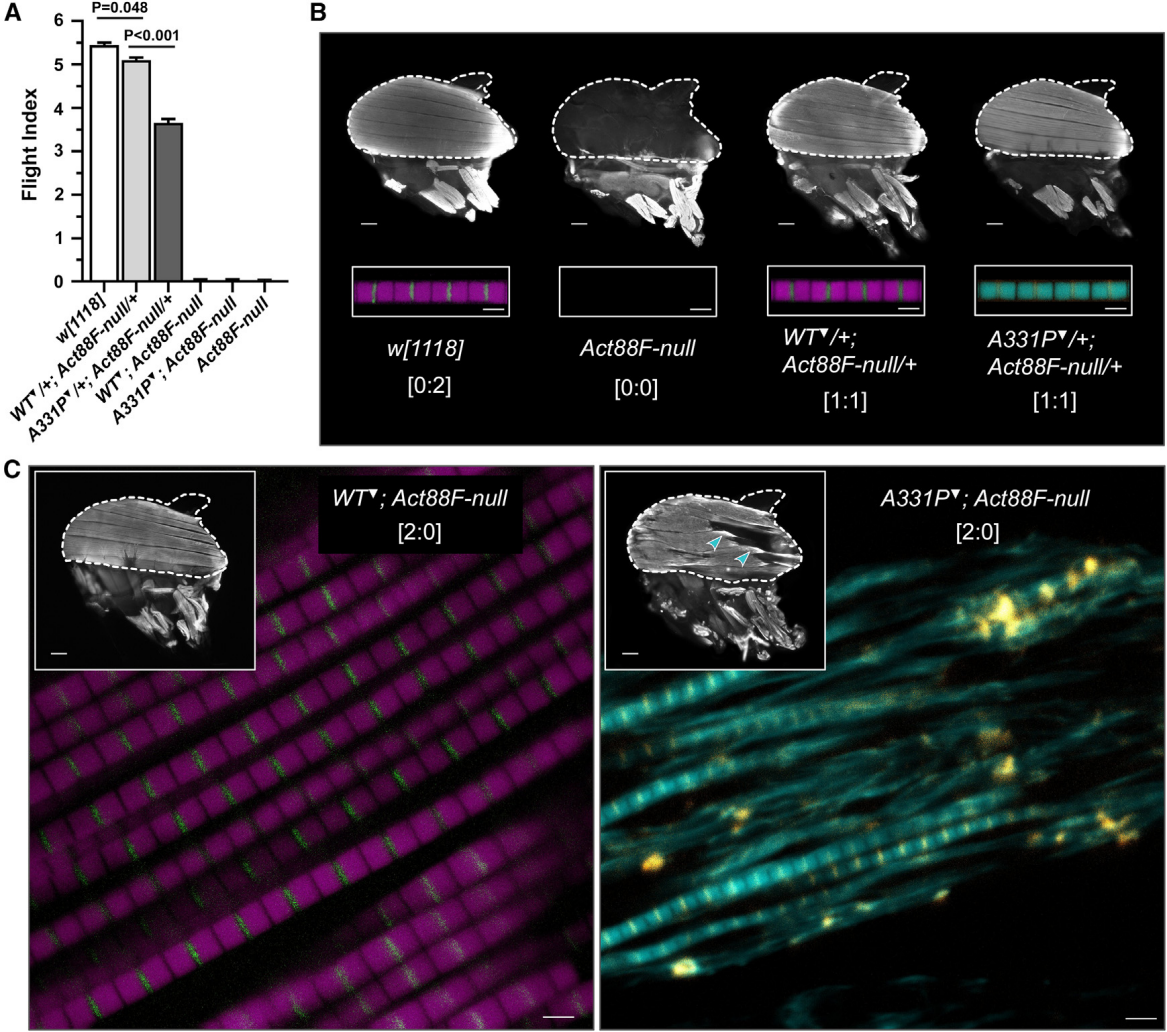
A331P actin causes *Drosophila* IFM hypercontraction and reduces thin filament length (A) Flight indices of *w[1118]* wild-type, *WT*^*▼*^*/+; Act88F-null/+* control, *A331P*^*▼*^*/+; Act88F-null/+* heterozygous mutant, *WT*^*▼*^*; Act88F-null* control, *A331P*^*▼*^*; Act88F-null* homozygous mutant, and *Act88F-null Drosophila*. Relative to *w[1118]*, *WT*^*▼*^*/+; Act88F-null/+* control *Drosophila* exhibited a slight, but significant reduction in flight ability. *A331P*^*▼*^*/+; Act88F-null/+* heterozygous mutants displayed a significantly lower flight index vs. *WT*^*▼*^*/+; Act88F-null/+* control flies. *WT*^*▼*^*; Act88F-null*, *A331P*^*▼*^*; Act88F-*null, and *Act88F-null Drosophila* were flightless. Data are presented as mean ± SEM. Significant differences in flight ability were assessed using the Kruskal–Wallis test (*n* = 173–657 flies/genotype). (B) Fluorescent micrographs of Alexa 568-phalloidin-stained dorsal longitudinal IFMs (DLMs) of two-day-old *w[1118]* wild-type, *Act88F-null*, *WT*^*▼*^*/+; Act88F-null/+* control, and *A331P*^*▼*^*/+; Act88F-null/+* heterozygous mutant *Drosophila*. The cuticle from each hemi-thorax is outlined to illustrate orientation. Numbers in brackets indicate transgenic vs. endogenous *Act88F* allele copy number. *w[1118]* wild-type, *WT*^*▼*^*/+; Act88F-null/+* control, and *A331P*^*▼*^*/+; Act88F-null/+* heterozygous mutant *Drosophila*, displayed normal DLM morphology with the six fibers spanning the length of the thorax. Scale bars = 100 μm. Insets: individual IFM myofibrils from these three genotypes appeared normal, with well-ordered, highly-uniform sarcomeres. Control and mutant thin filaments are shown in magenta and cyan respectively, while α-actinin-labelled Z-lines are displayed as green/yellow. Note the absence of F-actin fluorescence emission originating from the DLMs and myofibrils of *Act88F-null* thoraces. Scale bars = 2 μm. (C) Despite a lack of flight, *WT*^*▼*^*; Act88F-null* control flies were characterized by highly-ordered, intact, structurally sound DLMs (inset; scale bar = 100 μm), myofibrils, and sarcomeres. *A331P*^*▼*^*; Act88F-null* homozygous mutants however, exhibited hypercontracted and torn DLMs (inset, blue arrowheads; scale bar = 100 μm). *A331P*^*▼*^*; Act88F-null* IFM myofibrils were thin, damaged, disordered and frequently appeared degenerated. Moreover, individual sarcomeres, and thus thin filaments, were shorter and highly compromised. Scale bars = 2 μm.

A lack of A331P ACTC animal models, that phenocopy HCM, hinders our ability to confirm the pathogenicity and *in vivo* effects of the variant. Therefore, to first delineate if A331P actin expression is sufficient to disrupt the contractile properties and/or the histostructural features of muscle, we generated several transgenic *Drosophila* lines. Flies permit interrogation of mutant proteins in diverse striated muscles, which helps verify the variants’ pathological significance in mature tissues, that experienced native myogenesis. *Drosophila* indirect flight muscles (IFMs) consist of antagonistic dorsolongitudinal (DLM) and dorsoventral fibers that are dispensable under laboratory conditions, and are highly susceptible to sarcomeric mutation.^19^^,^^20^^,^^28^^,^^29^^,^^38^^,^^39^^,^^40^^,^^41^ *Drosophila*, like avian and mammalian species, possess six actin genes. *Act88F* encodes IFM sarcomeric actin.^38^^,^^42^ We produced flies with different copy numbers of genes encoding transgenic and/or endogenous actin by backcrossing *Act88F*^*WT*^ or *Act88F*^*A331P*^ transgenes into flightless, *Act88F*-null animals (see STAR Methods, Table 1, experimental model and study participant details). Two-day-old *w[1118]* wild-type *Drosophila* displayed a flight index (FI) of 5.45 ± 0.04, consistent with predominantly upward directed flight patterns (Figure 2A). As shown previously,^19^ age-matched *WT*^*▼*^*/+*; *Act88F-null/+* controls, expressing *Act88F*^*WT*^ from one transgenic and one endogenous wild-type allele, exhibited a slightly lower FI of 5.10 ± 0.06 relative to *w[1118]*. Two-day-old *A331P*^*▼*^*/+; Act88F-null/+* “heterozygotes” had an FI of 3.66 ± 0.09. The difference in flight ability vs. *WT*^*▼*^*/+*; *Act88F-null/+* controls was highly significant. Increasing the transgenic to endogenous *Act88F* allele number to 2:1, further hampered flight ability of both control and mutant lines (Figure S1). Nonetheless, *A331P*^*▼*^*; Act88F-null/+* (FI = 1.83 ± 0.05) performed significantly worse than their *WT*^*▼*^*; Act88F-null/+* (FI = 4.26 ± 0.08) counterparts. As seen in flies completely lacking Act88F, expression of wild-type or mutant transgenic actin, in *WT*^*▼*^*; Act88F-null* or *A331P*^*▼*^*; Act88F-null Drosophila*, resulted in a loss of flight (Figure 2A). Hence, as observed earlier^19^ and discussed below, expression of two mutant, or even of two wild-type transgenes alone, could not reestablish IFM function of *Act88F*-null flies.

**Table 1:** Genotypes of fly stocks used in this stud**y**

	Genotype	TG:Endo ratio^a^	Shorthand
1	*w[1118]*	0:2	*w[1118]*
2	*Act88F*^*KM88*^	0:0	*Act88F-null*
3	*w[∗]; P{caryP, w[+], Act88F-WT}attP40; +*	2:2	*WT*^*▼*^
4	*w[∗]; P{caryP, w[+], Act88F-A331P}attP40; +*	2:2	*A331P*^*▼*^
5	*w[∗]; P{caryP, w[+], Act88F-WT}attP40; Act88F*^*KM88*^	2:0	*WT*^*▼*^; *Act88F-null*
6	*w[∗]; P{caryP, w[+], Act88F-A331P}attP40; Act88F*^*KM88*^	2:0	*A331P*^*▼*^; *Act88F-null*
7	*w[∗]; P{caryP, w[+], Act88F-WT}attP40/+; Act88F*^*KM88*^*/+*	1:1	*WT*^*▼*^*/+*; *Act88F-null/+* (Progeny of #2 and #3)
8	*w[∗]; P{caryP, w[+], Act88F-A331P}attP40/+; Act88F*^*KM88*^*/+*	1:1	*A331P*^*▼*^*/+*; *Act88F-null/+* (Progeny of #2 and #4)
9	*w[∗]; P{caryP, w[+], Act88F-WT}attP40; Act88F*^*KM88*^*/+*	2:1	*WT*^*▼*^; *Act88F-null/+* (Progeny of #3 and #5)
10	*w[∗]; P{caryP, w[+], Act88F-A331P}attP40; Act88F*^*KM88*^*/+*	2:1	*A331P*^*▼*^; *Act88F-null/+* (Progeny of #4 and #6)
11	*y[1] w[∗]; Mi{Trojan-GAL4.0}Hand[MI04106-TG4.0]*		*Hand*^*4.2*^*-Gal4*
12	*w[∗]; P{caryP, w[+], UASp::Act57B-WT}attP40*		*UAS-Act57B*^*WT*^
13	*w[∗]; P{caryP, w[+], UASp::Act57B-A331P}attP40*		*UAS-Act57B*^*A331P*^
14	*y[1] w[∗]/w[∗]; Mi{Trojan-GAL4.0}Hand[MI04106-TG4.0]/P{caryP, w[+], UASp::Act57B-WT}attP40*		*Hand>Act57B*^*WT*^ (Progeny of #11 and #12)
15	*y[1] w[∗]/w[∗]; Mi{Trojan-GAL4.0}Hand[MI04106-TG4.0]/P{caryP, w[+], UASp::Act57B-A331P}attP40*		*Hand>Act57B*^*A331P*^ (Progeny of #11 and #13)

a TG:Endo ratio: Ratio of the number of copies of transgenic to endogenous actin alleles.

We next assessed IFM morphology from flash-frozen, Alexa 568-phalloidin-labelled hemi-thoraces to determine if A331P actin expression was associated with overt structural anomalies in the mutant fibers. At two days of age, both *WT*^*▼*^*/+; Act88F-null/+* control and *A331P*^*▼*^*/+; Act88F-null/+* flies displayed prominent, intact DLMs that spanned the thorax and were phenotypically normal when compared to *w[1118]* (Figure 2B). Tissue morphology in *WT*^*▼*^*; Act88F-null* animals was likewise normal, despite the lack of flight (Figure 2C left, inset). The DLMs of *A331P*^*▼*^*; Act88F-null Drosophila* however, which were also flightless, exhibited destructive hypercontraction with complete penetrance (Figure 2C right, inset, blue arrowheads). This phenotype results from excessive and/or poorly regulated force generation, frequently due to Tpm-based disinhibition of myosin crossbridge formation and cycling.^39^^,^^41^ Closer examination additionally revealed a thinning and streaming of mutant myofibrils, and frequent loss of the highly ordered and repetitive arrangement of sarcomeres. Sarcomere and concomitant thin filament shortening were also markedly evident.

### A331P actin causes cardiomyopathy in *Drosophila* characterized by enhanced basal contractile activity

**Figure 3:**
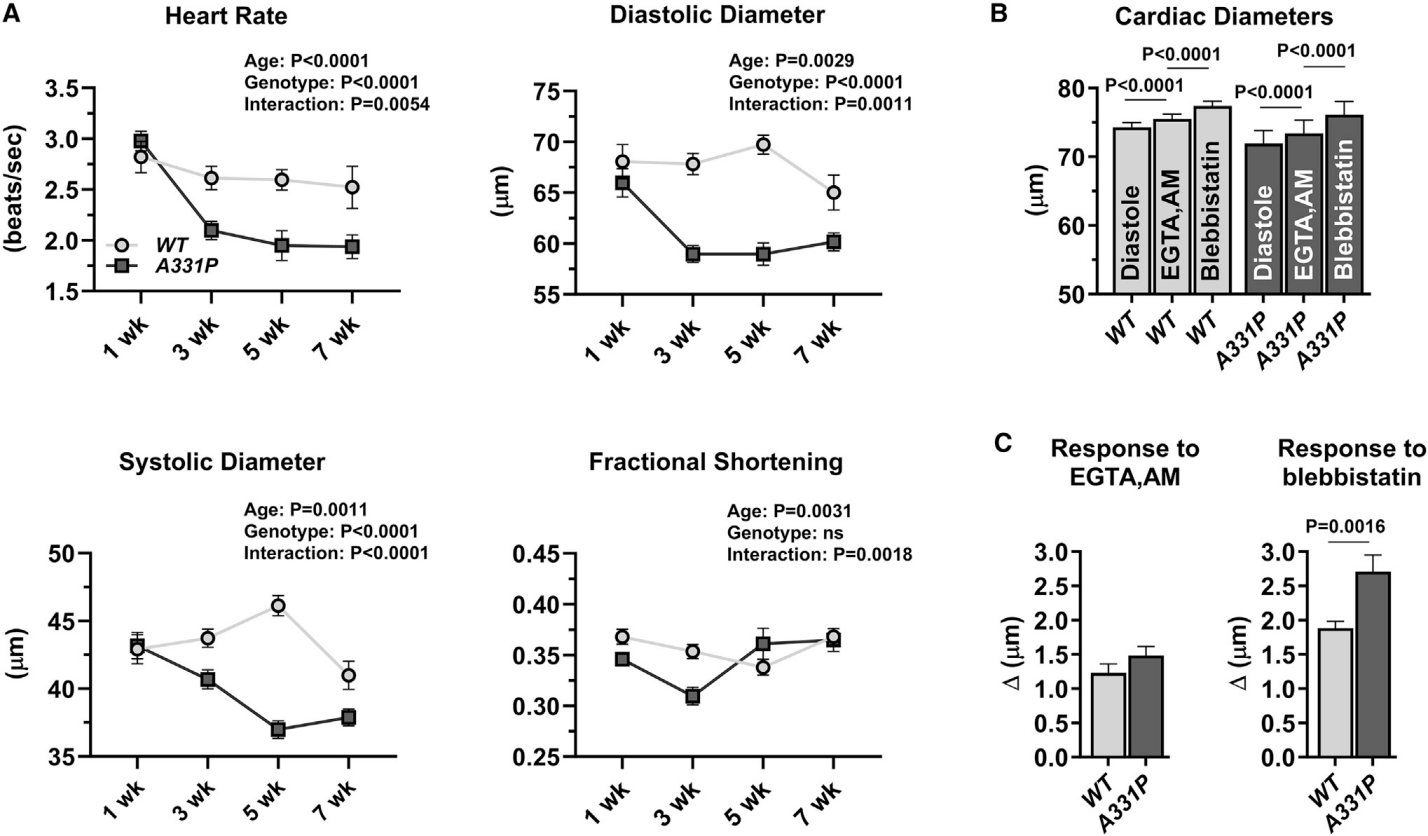
Expression of A331P actin results in restrictive physiology and impaired relaxation in *Drosophila* heart tubes (A) Heart-restricted expression of *Act57B* A331P mutant actin significantly altered several indices of cardiac function relative to wild-type *Act57B*. *WT* = *Hand>Act57B*^*WT*^; *A331P* = *Hand>Act57B*^*A331P*^. *Hand>Act57B*^*A331P*^*Drosophila* displayed significantly decreased heart rates and cardiac diameters, that worsened with age, relative to controls. Data are presented as mean ± SEM. Significant differences between genotype and age, and interaction effects, were determined using two-way ANOVAs (*n* = 20–59 flies/age/genotype). (B) Significant, stepwise increases in cardiac diameters were observed in *Hand>Act57B*^*WT*^ and *Hand>Act57B*^*A331P*^ flies following extra- and intra-cellular Ca^2+^ chelation and, again, upon exposure to blebbistatin. Data are presented as mean ± SEM. Increases in cardiac dimensions due to EGTA/EGTA,AM and to blebbistatin were evaluated using repeated measures ANOVAs followed by Tukey’s multiple comparison tests of the matched groups (*n* = 22–26 flies/genotype). (C) Left: the change in cardiac diameter in response to EGTA/EGTA,AM was similar between *Hand>Act57B*^*WT*^ and *Hand>Act57B*^*A331P*^*Drosophila*. Right: blebbistatin treatment resulted in a significantly greater degree of heart wall relaxation in *Hand*>*Act57B*^*A331P*^ relative to *Hand>Act57B*^*WT*^. Data are presented as mean ± SEM. Two-tailed unpaired t-tests were used to distinguish significant differences in cardiac diameter changes between genotypes (*n* = 22–26 flies/genotype).

The “dorsal vessel”, or fly “heart” for simplicity, is composed of a single layer of approximately 80 cardiomyocytes, which are aligned and conjoined along two opposing bilateral rows (Figure S2) to form a linear tube.^43^ *Act57B* encodes one of two cardiac sarcomeric actins.^44^^,^^45^ To determine the effects of the HCM variant on *Drosophila* dorsal vessel/heart function, *Hand*^*4.2*^*-Gal4*-expressing virgin females were crossed with males carrying either a wild-type or mutant actin transgene behind an upstream activating sequence (*UAS*). The progeny inherit both genes and express transgenic actin exclusively in the heart tube. Importantly, we previously showed that *Hand*^*4.2*^*-Gal4* drives similar levels of wild-type and mutant *UAS*-*Act57B* expression, yielding roughly 10% of total cardiac actin.^20^ Myocardial function was examined at multiple time points, using high-speed video microscopy and semi-automated optical heartbeat analysis software, to assess pathological changes with age.^46^ Myogenic heart rate decreased significantly among both genotypes over time, however, *Hand>Act57B*^*A331P*^ *Drosophila* had a more pronounced decline relative to control (Figure 3A). Cardiac tube diameters also decreased with age, with *Hand>Act57B*^*A331P*^ showing a far greater reduction in both diastolic and systolic dimensions vs. *Hand>Act57B*^*WT*^. However, because both diastolic and systolic diameters dropped at roughly the same rate for each line, significant genotypic differences in fractional shortening were not observed. Restricted heart tube diameters nonetheless jeopardize filling and stroke volume, which when combined with lower heart rates, inevitably compromise cardiac output in mutant animals relative to control. Thus, reminiscent of changes observed during human HCM, A331P actin expression prompted decreased resting chamber volumes, impaired diastolic filling, and reduced cardiac output in flies.

Given the structural properties inherent to the fly’s heart, we devised a protocol to decipher the mechanistic basis of diastolic restriction.^19^^,^^20^^,^^29^^,^^47^ Since the distance across the dorsal vessel’s lumen-forming, conjoined cardiomyocytes is directly proportional to cell length (Figure S2), which in turn is directly proportional to the degree of contractile activity, cardiac tube diameter can serve as a proxy measure of Tpm regulatory position along thin filaments and its myosin blocking capacity. Abnormal Ca^2+^ homeostasis, impaired relaxation, and diastolic dysfunction are hallmarks of HCM. To determine if disproportionate actomyosin interactions, triggered by elevated resting Ca^2+^ levels, contribute to *Hand>Act57B*^*A331P*^ diastolic restriction, we subjected beating, three-week-old control and mutant hearts to extra- and intracellular Ca^2+^ chelators, *in situ.*^19^^,^^20^^,^^29^^,^^47^ Cardiac tube contraction, in both lines, was completely halted following the addition of EGTA/EGTA-AM in excess of free Ca^2+^. Relative to those established during diastole, both *Hand>Act57B*^*WT*^ and *Hand>Act57B*^*A331P*^ hearts experienced modest, yet significant, increases in diameters (1.7% and 2.1%, respectively) upon EGTA/EGTA,AM incubation (Figure 3B). Thus, in both lines during diastole, trace amounts of free intracellular Ca^2+^ likely bind Tn and activate acto-myosin interactions to produce slightly contracted cardiomyocytes. There was no difference in the increase in cardiac diameters between *Hand*>*Act57B*^*WT*^ (Δ = 1.23 ± 0.13μm) and *Hand>Act57B*^*A331P*^ (Δ = 1.48 ± 0.13μm) upon treatment (Figure 3C, left). These results imply that during relaxation a similar amount of free Ca^2+^, within the cardiomyocytes of both genotypes, contributes equivalently to diastolic tension. Unaltered Ca^2+^ handling was also supported by qPCR analysis of mRNA abundance (Figure S3), which revealed no significant differences in transcript levels of L-type Ca^2+^ channels, ryanodine receptors, SERCA, Na^+^/Ca^2+^ exchangers, or in IP_3_ receptors between the lines. Therefore, elevated resting Ca^2+^ alone cannot account for the restricted mutant heart tubes.

Previously, we established that the subsequent exposure of EGTA/EGTA,AM-treated specimens to myosin-specific inhibitors elicits further increases in *Drosophila* cardiac dimensions.^19^^,^^20^^,^^29^^,^^47^ Consistent with our earlier studies, blebbistatin supplementation prompted additional, and statistically significant, increases in *Hand*>*Act57B*^*WT*^ (2.5%) and *Hand>Act57B*^*A331P*^ (3.7%) heart tube diameters (Figure 3B). These results indicate that, following near-complete Ca^2+^ chelation, a small population of myosin cross-bridges continues to bind and generate force along thin filaments. This is possible only if Tpm B-state-positioning is normally, to a small degree, inherently variable and the regulatory strand therefore fails to completely prevent acto-myosin cycling. To ascertain if the actin mutation potentially enhances Tpm’s B-state positional instability, and thereby increases the population of bound cross-bridges and the extent of myocyte shortening, we compared the myocardial responses to blebbistatin treatment. Myosin inhibition increased the diameter across *Hand>Act57B*^*A331P*^ heart tubes (Δ = 2.71 ± 0.24μm) significantly more compared to *Hand*>*Act57B*^*WT*^ (Δ = 1.88 ± 0.10μm) (Figure 3C, right). These data suggest that the diastolic dysfunction and restrictive physiology observed in *Hand>Act57B*^*A331P*^ *Drosophila* are independent of resting Ca^2+^ levels. Thus, the mutant myocardial phenotype likely results from excessively disinhibited cross bridge-binding to poorly blocked actin attachment sites along cardiac thin filaments, resulting in enhanced basal contractile activity and incomplete diastolic relaxation under low Ca^2+^.

Finally, we aimed to recapitulate the anomalous phenotype of enhanced contractile activity under low Ca^2+^, *in vitro*, at the molecular level. Here, using recombinant baculoviruses, human wild-type and A331P ACTC were expressed in *Sf21* insect cells and purified via gelsolin affinity chromatography.^37^ Following thin filament reconstitution with bovine ventricular Tn-Tpm, filament sliding speeds were determined under distinct steady-state Ca^2+^ conditions, in flow cells containing 125 μg/mL of rabbit skeletal heavy meromyosin (HMM), via *in vitro* motility (IVM) assays (Table 2). Interestingly, under high Ca^2+^ (i.e., pCa 5 and 4.5) both wild-type and mutant thin filaments were propelled by HMM at speeds that were not significantly different. However, at pCa 10 and 8, A331P ACTC thin filaments exhibited significantly faster sliding speeds relative to control, consistent with enhanced basal activity at the molecular scale.

**Table 2:** Sliding velocities of wild-type vs. A331P human ACTC-containing regulated and unregulated filaments

Filament type	pCa	Velocity (μm/s)	Significantly different from WT?
Recombinant hACTC^WT^ + Tn + Tpm	10	0.016 ± 0.106	
Recombinant hACTC^A331P^ + Tn + Tpm	10	0.402 ± 0.453	Yes, *p* < 0.0001
Recombinant hACTC^WT^ + Tn + Tpm	8	0.206 ± 0.655	
Recombinant hACTC^A331P^ + Tn + Tpm	8	0.653 ± 0.688	Yes, *p* < 0.0001
Recombinant hACTC^WT^ + Tn + Tpm	5	3.524 ± 1.124	
Recombinant hACTC^A331P^ + Tn + Tpm	5	3.194 ± 1.464	No, *p* = 0.0911
Recombinant hACTC^WT^ + Tn + Tpm	4.5	3.426 ± 1.220	
Recombinant hACTC^A331P^ + Tn + Tpm	4.5	3.669 ± 1.269	No, *p* = 0.1906
Recombinant hACTC^WT^	N/A	0.775 ± 0.310	
Recombinant hACTC^A331P^	N/A	0.964 ± 0.428	No, *p* = 0.3945
Recombinant hACTC^WT^ + Tpm	N/A	0.114 ± 0.130	
Recombinant hACTC^A331P^ + Tpm	N/A	0.294 ± 0.260	Yes, *p* = 0.0013

Recombinant human wild-type and A331P cardiac actins were purified following three separate *Sf21* insect cell infections, using recombinant baculoviruses. They were reconstituted into regulated thin filaments and sliding speeds determined at distinct Ca^2+^ concentrations (*n* = 90 filaments/condition), or polymerized into F-actin, differentially labeled with unique fluorophores (wild-type vs. mutant), and sliding speeds (*n* = >100 filaments/condition) assessed in the absence or presence of Tpm. Velocities are displayed as mean ± SD. Significance was determined using unpaired t-tests with Welch’s correction. *p* < 0.05 was considered significant.

IVM assays have additionally shown that, at low myosin concentrations, the addition of Tpm to F-actin, in the absence of Tn or Tn subunits, reduces filament sliding velocities.^29^^,^^48^^,^^49^^,^^50^ These observations are consistent with Tpm binding to a location along F-actin with inherent inhibitory bias. This default, azimuthal location of Tpm is dictated by the repetitive, highly favorable, interfacial electrostatic contacts between K326 and K328 of each actin subunit and every pseudo-repeat binding-module along Tpm (Figure 1), which position Tpm such that it impedes acto-myosin cycling.^4^^,^^6^^,^^9^^,^^19^^,^^20^^,^^28^^,^^29^^,^^30^^,^^32^^,^^51^ These associations are compulsory for the B-state and, thus, contribute to proper relaxation of striated muscle.^16^^,^^19^^,^^20^^,^^28^^,^^33^ Therefore, to determine if the A331P amino acid substitution potentially impairs Tpm’s intrinsic ability to block acto-myosin binding, due to Tpm mispositioning, we compared the sliding velocities of human wild-type and mutant F-actin, under sub-saturating myosin conditions (i.e., 35 μg/mL), in the absence and presence of Tpm. To facilitate resolving even minor discrepancies, we differentially labeled each recombinant F-actin type with a unique fluorophore and simultaneously assessed sliding velocities for wild-type vs. A331P filaments, directly, over the same bed of myosin, under identical experimental conditions (Table 2 and Figure S4). No significant difference was found in mean velocity between wild-type and A331P human F-actins. However, in the presence of Tpm, A331P filaments were propelled at a significantly higher average velocity relative to wildtype F-actin-Tpm. These data imply that Tpm is inherently less inhibitory along filaments comprising the variant, and more easily displaced by myosin, compared to controls.

### Cryo-EM-based reconstructions reveal negligible structural differences between human wild-type and A331P ACTC filaments

**Figure 4:**
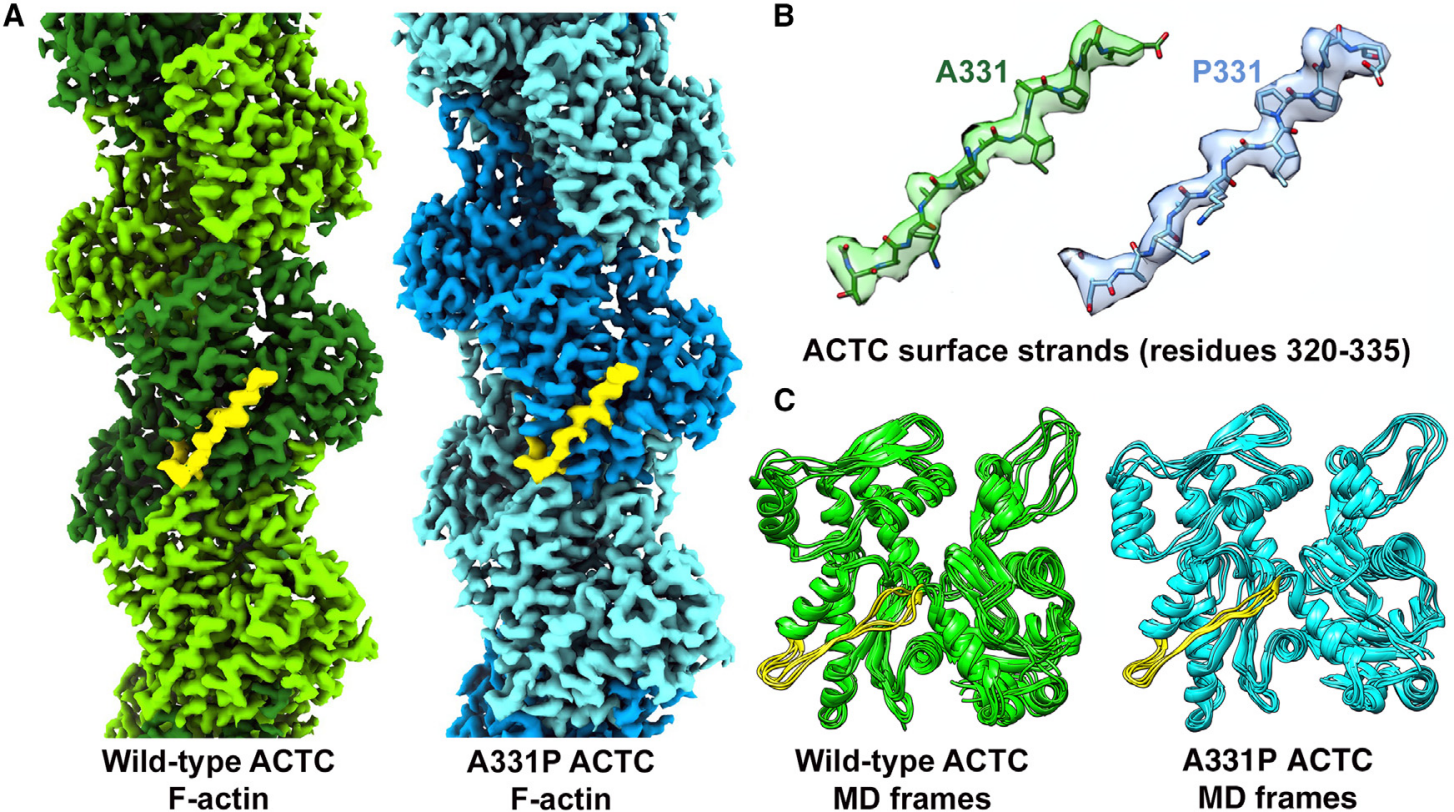
Cryo-EM reconstruction and MD analysis of human wild-type and mutant ACTC filaments reveal decreased A331P actin flexibility (A) Final cryo-EM maps for wild-type (green) and A331P (blue) filaments. Adjacent actin subunits are shaded differently in order to display the filament assembly and protomer arrangement. Density corresponding to the surface strands (residues 320–334) is rendered in yellow in the central subunits to highlight the general locus of residue 331. (B) Atomic models of the wild-type (green) and A331P (blue) mutant surface strands, spanning residues 320–335, are shown within their respective cryo-EM densities. Both maps fit well into the cryo-EM densities. No overt structural disparities were detected between the wild-type and mutant surface strands. (C) MD analyses of human wild-type and A331P ACTC. MD simulations were run on periodic wild-type and A331P ACTC filaments until the RMSD to the starting coordinates of the backbone atoms stabilized around a single value. Each actin monomer in the system was then superimposed on a central actin fitted using all actin backbone atoms. To compare the dynamic motions of the surface strand containing the A331P mutation, 14,000 or 18,369 aligned actin monomers from wild-type and A331P ACTC simulations, respectively, were then separated into clusters based on RMSD of the alpha carbons of residues 320–335. Shown are the representative structures of the 5 most-populated clusters from these analyses. Relative to wild-type, mutant A331P actin exhibited a drastic reduction in motion along the surface strand spanning residues 320–334.

Our results from diverse fly models and reconstituted vertebrate thin filaments, all suggest that the A331P HCM mutation impairs Tpm-based steric blocking of myosin binding at rest, leading to enhanced basal tension. Given its role in inhibitory Tpm positioning and B-state formation, we were particularly interested in the structural consequences of the mutation on the ACTC 320–334, K326/328-containing strand. We therefore carried out cryo-EM single particle analysis of human wild-type and A331P ACTC filaments to directly resolve potential molecular aberrations in the surface strand that may account for the elevated diastolic contractile activity. Both wild-type and mutant F-actins exhibited similar overall features in cryo-EM micrographs, consistent with the mutation preserving filament formation function (Figure S5A). Using the helical processing workflow in RELION, we resolved actin structure and connectivity in wild-type and A331P filaments at up to 3.5 Å resolution, enabling us to build atomic models of the complexes (Figures 4A, 4B, and S5B). Overall, both structures were very similar, exhibiting an alpha carbon root-mean-square deviation (RMSD) of 0.536 Å (Figure S6) between the two. Direct comparison of the atomic models of residues 320–335 from wild-type and mutant ACTCs surprisingly illustrated that the backbones of their surface strands were nearly superimposable (Figure 4B). Thus, cryo-EM reconstruction alone did not resolve major differences in the orientation of the ACTC 320–334 strand or in its individual constituent amino acids’ positions. We therefore conclude that the changes observed in our A331P-expressing animal models and in mutant ACTC filament motility parameters were not due to underlying static changes in actin structure detectable by cryo-EM, but alternatively may be attributed to changes in protein dynamics.

### The A331P mutation reduces actin flexibility and attractive forces between the ACTC 320–334 surface strand and tropomyosin

Our MD simulations from earlier work suggested that reduced flexibility of actin subregions, including the 320–334 surface strand, could disrupt the formation of interfacial F-actin-Tpm electrostatic contacts and the azimuthal stability of inhibitory Tpm positioning.^20^ Therefore, to investigate potential A331P-induced changes in protein dynamics, as well as possible structural alterations not readily resolved in our F-actin reconstructions, we performed MD simulations of wild-type and A331P ACTCs using models derived directly from our cryo-EM-based maps (Figure 4A). 50 ns MD simulations were run in explicit solvent boxes, which incorporated boundary conditions to emulate infinitely long filaments. The filaments contained wild-type or A331P protomers, to model F-actin and thin filament backbones. Consistent with our cryo-EM results, analysis of alpha carbon RMSD values for averaged MD protomers, following their initial alignment, showed no major discrepancies between wild-type and A331P structures (Figure S6). However, while mutant A331P ACTC protomers exhibited dynamic motion similar to wild-type overall, notable differences were seen among residues corresponding to the most dynamic subregions of actin, including the N- and C-termini, the D loop, the subdomain-4 linker, as well as the actin surface strand containing residues 320–334 (Figures 4C and S6 and S7). On average, the strand for the mutant actin was less flexible. To visualize this reduced motion, data for the central actin protomers in the model were extracted from representative F-actin MD frames by cluster analysis, superimposed, and compared (Figure 4C). The observed mutation-induced reduction in 320–334 surface strand mobility was further corroborated by decreased root-mean-square-fluctuations (RMSF), particularly in the vicinity of proline 333 of A331P ACTC (Figure S7), which fluctuated ∼0.6 Å less about its mean position compared to that of control. Thus, our F-actin MD results suggest that wild-type subunits, and importantly regions of the 320–334 surface strand, appear to exhibit greater mobility than the A331P mutant.

**Figure 5:**
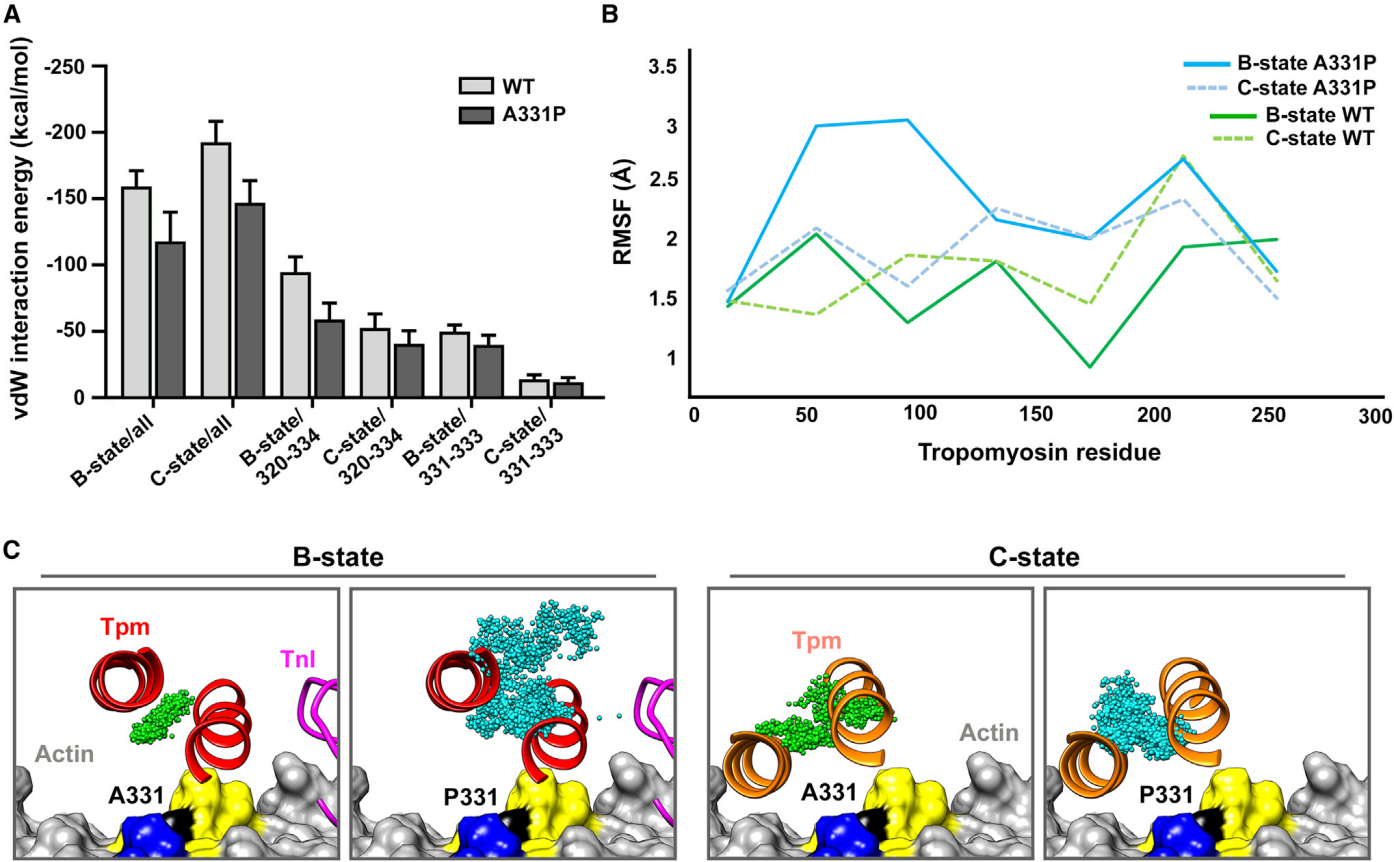
The A331P mutation decreases attractive F-actin-Tpm vdW forces and results in greater Tpm inhibitory positional variance (A) vdW interaction energy between Tpm (in the B-state or C-state) and ACTC F-actin (including all residues, residues 320–334, or just 331–334) was measured over the last 20–30 ns of MD simulation time. Bar graphs represent the average values ±SD. All energetic differences in vdW forces between wild-type actin and Tpm vs. A331P actin and Tpm were highly significant (*p* < 0.0001). Significance was assessed via unpaired t-tests (*n* = 135–200 total frames/genotype). (B) RMSF was calculated over the same MD simulation time frames as in (A) for the superhelix of Tpm as determined by Twister, in either the B- or C-state position along wild-type (500 frames, green) or A331P mutant (1000 frames, cyan) thin filament models. Prior to calculation, frames were aligned to the actin underlying the Tpm superhelical residue. Note, the greater degree of fluctuation of B-state Tpm along A331P vs. wild-type thin filaments, whilst the C-state RMSF values were considerably less disparate between the two models. (C) Cut away view showing Tpm (red/salmon ribbons) near residue 90 in the B-state (left) and C-state (right) configurations relative to actin (gray surface with residue 331 in black, K326/328 in blue, and P333 in yellow) and the TnI peptide (magenta ribbons). The distribution of individual Tpm superhelical positions from (B) at Tpm residue 90, during the last 10 ns of the MD simulation, are overlaid and depicted as small spheres from the wild-type (green) and A331P (cyan) thin filament simulations. 1000 frames for each are shown, revealing the dynamic movement of the Tpm coiled coil in the vicinity of the A331P mutation site. Each frame examined was aligned to the initial actin coordinates underlying Tpm residue 90. This was done to account for any movements of the actin monomers during the simulation and to superimpose the results from the two B- or C-state Tpm dimers in the system. The Tpm superhelix position along wild-type thin filaments varied little in the B-state, whereas the same region of Tpm along A331P mutant filaments showed a broad positional distribution. The C-state superhelical positions at Tpm residue 90 were similar for both wild-type and A331P thin filaments. Thus, particular Tpm pseudo-repeats along the mutant filament are predicted to be less effective at blocking myosin binding sites in the B-state.

We speculated that by restricting 320–334 strand dynamics, the mutation disrupts formation of the B- and possibly C-states, and/or the well-controlled regulatory switching between them. Specifically, we hypothesized that the mutation may hamper Tpm’s ability to adequately block basal actomyosin associations, leading to impaired contractile inhibition at rest, and therefore leading to pathology. To test this hypothesis, 40–50 ns MD simulations were performed on wild-type and A331P filaments in explicit solvent as above. However, here, each long pitch helix of either wild-type or mutant F-actin was decorated with a pair of Tpm dimers and residues 89–151 of TnT covering the head-to-tail junction to strengthen the end-to-end overlap of successive Tpms. One long pitch actin helix was built with Tpm in its C-state configuration, whereas the other was built with Tpm in its B-state configuration. The latter also included residues 135–210 of TnI in its actin-bound conformation to incorporate key interactions that help stabilize the B-state. By running the system with independent B- and C-state Tpm conformations on either side of the same filament, average mutation-induced positional changes could be directly addressed in a single simulation for both states. Once convergence of RMSD values of Tpm relative to the starting structure was established in each regulatory state, electrostatic and vdW interaction energies between Tpm and actin, were determined (Figures 5 and S8–S16).

The results of the simulations showed reduced interactions of Tpm with A331P compared to wild-type actin. The mutant filaments exhibited a marked reduction in the vdW Tpm-actin interaction energy (B-state, 26%; C-state, 24%) vs. wild-type (Figure 5A), while the electrostatic interactions differed marginally in both the B- (4%) and C- (5%) states (Figures S8–S16). These A331P-associated reductions are consistent with decreased Tpm-actin affinity. Calculating the interaction energy between Tpm and actin residues 320–334 showed that most of the mutation-induced loss in interaction energy in the B-state model resulted from altered Tpm interactions with the ACTC surface strand (Figure 5A). Additional analysis of Tpm-actin interactions was performed by determining the superhelical axis positions of Tpm during the MD simulation, as measured by the program Twister.^52^ The positions were calculated at Tpm residues near amino acid 331 of actin in each pseudo-repeating binding module (i.e., at Tpm residues 13, 51, 90, 128, 168, 208, and 246) after alignment of the underlying ACTC monomer for each residue (Figures 5B and S10–S16). Comparison of the RMSF values for Tpm at residues 51, 90, 128, 168, and 208 showed increased fluctuation along the mutant F-actin backbone compared to Tpm along wild-type filaments for the B-state (Figures 5B, 5C, and S10–S16), particularly at residues 51 and 90 (0.96 Å and 1.76 Å, respectively). Measuring the Tpm superhelix-filament axis distances at residues 51 and 90 showed that the A331P mutant actin surface was farther from Tpm than that of wild-type actin (42.0 Å vs. 39.6 Å for residue 51, and 41.0 Å vs. 37.6 Å for residue 90, respectively). Thus, with increased fluctuations and distances from the F-actin surface, it is not surprising that both electrostatic and vdW interaction energies were reduced in A331P B-state filaments. These changes likely diminish the steric blocking ability of Tpm along mutant filaments, potentially allowing for myosin binding even under relaxing conditions. We also observed differences between the C-state superhelical positions, but these were relatively minor compared to the B-state differences (Figures 5B, 5C, and S10–S16). However, even minor disturbances to the C-state, combined with B-state destabilization, could enhance contractile activity at rest and may explain the hypercontraction and diastolic dysfunction in our mutant fly models, as well as heightened A331P thin filament motility under low Ca^2+^, *in vitro*.

## Discussion

HCM is a genetically and clinically heterogeneous disorder, predominantly caused by mutations in genes encoding sarcomeric proteins.^21^^,^^23^^,^^24^^,^^25^ Disease hallmarks include thickened, hypercontractile myocardium, diastolic dysfunction, and impaired relaxation. The ACTC A331P HCM-causing mutation has been the subject of multiple investigations since its initial identification over two decades ago.^35^^,^^36^^,^^37^^,^^53^^,^^54^ One study found that A331P ACTC mouse models displayed no significant differences in cardiac systolic function, wall thickness, and left ventricular dimension relative to wild-type littermates.^36^ A second study reported that bovine trabeculae, reconstituted with thin filaments composed of recombinant human A331P ACTC, exhibited reduced maximum tension and hypocontractility.^37^ These results contrast those stemming from other HCM mutations, which routinely engender a gain-in-sarcomeric function, and cellular- and chamber-level hypercontractility and, thus, they challenge current pathogenic paradigms.^21^^,^^23^^,^^24^^,^^25^ However, under baseline conditions, transgenic mice often fail to faithfully reproduce HCM phenotypes, and following thin filament extraction and reconstitution in permeabilized bovine trabeculae, Bai et al.^37^ provided no evidence of ultra-structurally-preserved sarcomeres or crystalline-like arrays of interdigitating myofilaments. Thus, damaged, poorly restored, and/or misaligned macromolecular assemblies of thick and mutant thin filaments, as well as defective F-actin-myosin binding protein-C interactions within the reconstituted sarcomeres, may influence, confound, and/or complicate data acquisition and interpretation.^37^^,^^54^

To further scrutinize A331P ACTC-mediated HCM pathogenesis, we employed a multidisciplinary approach. We hypothesized that the variant disrupts the Tpm B-state and acto-myosin inhibition at rest, leading to impaired relaxation and diastolic dysfunction, early drivers of HCM in humans.^24^^,^^25^ To first validate the pathogenicity of A331P actin, and its potential to provoke HCM hallmarks in distinct striated muscles, we built several transgenic fly lines. Our *Drosophila* models benefit from reduced genetic redundancy and physiological complexity, which can help illuminate myopathic processes and resolve genotype-phenotype associations.^19^^,^^20^^,^^29^^,^^47^ Moreover, thin filament-based regulation of striated muscle contraction is conserved, and the fly dorsal vessel is characterized by a proteomic biosignature and physiological properties that are clearly myocardial in nature.^39^^,^^40^^,^^45^^,^^55^ Pathologically, as observed in higher organisms, the *Drosophila* heart also distinctly remodels in response to a gain- vs. loss-of-sarcomeric function.^19^^,^^20^^,^^29^^,^^40^^,^^47^ Nonetheless, it is important to emphasize obvious differences between the cardiovascular systems of insects and higher vertebrates, which in flies consists of a single-chambered tubular organ and an open-circulatory system, operating under low hydrostatic pressure.^56^^,^^57^ The four-chambered human heart pumps oxygen-rich blood, which is essential to meet high metabolic demands and for viability, through a closed, high-pressure, circulatory system. The dorsal vessel is required for transporting nutrients and signaling molecules, but not oxygen, and can thus be severely compromised without being lethal. Therefore, the *Drosophila* heart represents an attractive system for modeling human cardiac diseases including cardiomyopathy, however, as with any animal model, evolutionary distance and fundamental cardiovascular differences may affect the translational impact of certain data acquired in insects.

Here, we first assessed flight in animals that expressed varying copy numbers of transgenic and endogenous *Act88F*. Flight ability was inversely related to both wildtype and mutant transgene copy number (relative to endogenous *Act88F*) with *Act88F*^*A331P*^-expressing flies however, invariably performing worse than corresponding controls (Figure 2A). Because the *attP40* transgene insertion locus lies upstream or within the first intron of specific *msp-300* isoforms,^58^ the slight reduction in *WT*^*▼*^*/+; Act88F-null/+* flight relative to *w[1118]* implies the landing site itself may serve as an insertional mutation that modestly impairs IFM performance in adults.^59^ Flight was nevertheless retained (albeit reduced) in most transgenic controls, and since the genotypes of all flies being compared were identical, except for a single nucleotide of the integrated construct, the exaggerated impairment observed among mutants is directly attributable to the A331P actin mutation. The inability of two mutant, or even of two wild-type transgenes to reestablish IFM function in *Act88F*-null flies, is likely related to aberrant regulation of transgene expression and stoichiometric protein imbalances.^19^^,^^60^ The correct stoichiometry of sarcomeric elements, especially actin as it is the most stoichiometrically abundant myofibrillar protein, is critical for proper integrity and function of the flight musculature.^61^ Thus, the negative impact of protein imbalances on the IFM is likely heightened as the proportion of poorly transcriptionally regulated *Act88F* transgenes per genotype increases.

*Act88F* A331P actin, in the absence of wild-type protein, caused IFM hypercontraction (Figure 2C). Thin filament length is a key determinant of sarcomeric force production.^62^ Previously, recombinant human A331P ACTC was shown to polymerize faster than wild-type ACTC, *in vitro,*^37^ which could increase filament length, *in vivo. A331P*^*▼*^*; Act88F-null* IFM thin filaments, however, were exceedingly short relative to control (Figure 2C). This indicates that IFM hypercontraction was not caused by increased thin filament length and resultant elevated force, but rather was likely due to disrupted Tpm-based inhibition of force production, as previously observed.^19^^,^^20^^,^^28^^,^^39^^,^^40^^,^^41^ When expressed in the *Drosophila* heart, *Act57B* A331P actin reduced cardiac tube diameter (i.e., cardiomyocyte length) and output (Figure 3). These findings are consistent with hypercontractility, impaired relaxation and diastolic dysfunction that, in patients, precede hypertrophy.^24^^,^^25^ In our mutant heart model, elevated diastolic Ca^2+^ and/or poor Tpm B-state positioning potentially stimulated the excessive cardiomyocyte shortening observed at rest. However, measurement of dorsal vessel diameters following sequential treatment of fully intact fly hearts with EGTA/EGTA,AM, and then blebbistatin, revealed that the mutant’s cardiac restriction was unlikely due to altered Ca^2+^-handling, but rather caused by poorly blocked myosin-binding sites, leading to a disproportionately greater number of actively cycling cross-bridges during diastole (Figures 3B and 3C). This abnormal phenotype, of enhanced basal contractile activity at the cellular-/organ-level, was also demonstrated at the single molecule level via assessing *in vitro* sliding properties of thin filaments reconstituted from recombinant human ACTC and bovine ventricular Tn-Tpm (Table 2). No differences in myosin-driven sliding speeds were observed between the filaments under high Ca^2+^ conditions. However, reconstituted thin filaments containing the mutant A331P ACTC F-actin backbone were propelled by myosin at significantly higher speeds at low Ca^2+^ relative to wildtype. Therefore, together, these data point to disrupted resting thin-filament regulation as the underlying stimulant of A331P actin-mediated pathogenesis.

Efficient and effective thin filament-based regulation of acto-myosin cycling and contraction requires the reversible translocation of Tpm among the well-described B-, C-, and M-state positions.^2^^,^^3^^,^^6^^,^^14^^,^^15^^,^^16^ The B-state represents a thin filament molecular configuration that is compulsory for contractile inhibition and proper relaxation during diastole. It is established not only by Tn-F-actin binding, but also by numerous contacts between every actin subunit and Tpm.^4^^,^^6^^,^^9^^,^^19^^,^^20^^,^^28^^,^^29^^,^^30^^,^^31^^,^^32^ Notably, these include favorable and indispensable electrostatic associations between K326 and K328, residing along actin’s 320–334 surface strand, and each Tpm pseudo-repeat binding module.^6^^,^^14^^,^^15^^,^^16^^,^^19^^,^^20^^,^^28^ Given the locus of the HCM-causing ACTC A331P substitution, we anticipated that the mutation would distort the K326/328-containing surface strand and thereby compromise the formation of essential B-state stabilizing electrostatic contacts.

To test this, we performed cryo-EM reconstruction of F-actins composed exclusively of human recombinant wild-type or A331P ACTC. Surprisingly, as also recently noted by Huang et al.,^63^ we found that the two F-actin structures were virtually indistinguishable (Figures 4A and 4B). The residue-level perturbations mentioned in Huang et al. are quite small and may reflect changes in side-chain rotamer assignments, without necessarily truly involving large alterations in side chain positions. Thus, we regard differences between the wild-type and mutant actins as more likely due to dynamic effects, where the main actin chains are very similar on average, but can transiently adopt unique conformations. Resolving such differences, which are poorly detected in static cryo-EM-derived models, therefore demands the use of MD simulations.

50 ns simulations of our EM-based F-actin models revealed that parts of the 320–334 surface strand were more mobile in wild-type vs. mutant protomers (Figures 4C and S7). These results are reminiscent of our earlier findings, which illustrated that the M305L ACTC mutation likewise reduced actin’s flexibility, including the stretch of residues containing K326, K328, and A331.^20^ However, unlike the dampened mobility caused by the M305L substitution, which reduced the variant’s electrostatic bonding energy with Tpm, MD on A331P thin filament models, replete with Tn-Tpm regulatory components, showed that the reduced surface strand flexibility caused by the A331P mutation was predominantly associated with diminished vdW Tpm-actin interaction forces (Figures 5A, S8, and S9). We therefore posited that as with electrostatic bonds, altering non-polar, interfacial F-actin-Tpm contacts may similarly influence Tpm regulatory activity. We observed considerably higher regional RMSF values for certain Tpm pseudo-repeats in the B-state along A331P filaments, compared to wild-type, while the C-state remained mostly unperturbed (Figures 5B, 5C, S8, and S10–S16). Thus, a certain degree of strand mobility may optimize structural matching of complementary F-actin-Tpm surface contours to facilitate the formation of intermolecular electrostatic as well as vdW bonds and, consequently, the inhibitory B-state.^20^ These findings imply that an A331P-mediated increase in Tpm positional variability, due to diminished vdW contacts with the mutant actin’s 320–334 surface strand, may impair Tpm-based steric blocking and permit enhanced myosin binding at rest.

While these *in silico* findings corroborate our multiplexed IVM results, which resolved higher A331P F-actin-Tpm sliding speeds relative to wild-type (Table 2 and Figure S4), suggesting that the amino acid substitution directly hampers inhibitory Tpm positioning, they do not exclude the potential impact of the mutation on Tn’s contribution to the B-state. Our MD analyses, however, also permitted calculation of interaction energies between TnI and actin, which were reduced in mutant filaments. Residues 165–210 of TnI make extensive contacts with both Tpm and actin in the B-state model, presumably stabilizing Tpm in a blocking configuration. The electrostatic interaction energy between TnI residues 165–210 and A331P actin was largely decreased in the B-state simulation (−205.3 kcal/mol in wild-type vs. 7.1 kcal/mol in A331P). A potential source of the reduced electrostatic energy is unfavorable charge interactions between the C-terminus of TnI and the variant, possibly due to mutation-induced propagated effects extending to the actin D loop. Thus, while the MD data suggest that the Tpm-TnI complex forms on A331P mutant actin, it is potentially not bound as tightly compared to wild-type. It also remains tempting to consider additional allosteric effects stemming from the A331P substitution on the D loop and on the C-terminus of actin, which both displayed enhanced RMSFs in mutant actin MD simulations relative to control (Figure S7). These differences may impact putative B-state stabilizing, N-terminal TnT-actin associations that have been proposed to occur at low Ca^2+^ between the “TnT1-loop” and the D loop and C-terminus of neighboring actin monomers.^32^ However, because Tn is tethered to Tpm, greater azimuthal variability in Tpm B-state positioning will yield erratic Tn positioning, potentially hindering TnT1-and/or TnI-F-actin binding. Thus, individually or in sum, these molecular insults are expected to reduce the effectiveness of steric blocking, leading to disinhibition at rest and enhanced contractile activity.

Previous reports have discussed the possible contribution of vdW forces to F-actin-Tpm binding, including how Tpm mutations impact its flexibility as well as its interactions with and positioning on F-actin.^11^^,^^12^^,^^13^^,^^18^ Here, we provide evidence that actin mutations may similarly reduce these short-range forces locally and elicit a greater degree of Tpm B-state positional instability, underscoring possible effects of vdW forces in normal inhibitory Tpm positioning. The ACTC A331P HCM mutation seemingly weakens some of these F-actin-Tpm intermolecular associations which, subsequently, may perturb thin filament-based inhibition of diastolic acto-myosin activity. The variant marginally impacted C-state positioning of Tpm *in silico* and did not affect sliding speeds of reconstituted thin filaments at high Ca^2+^, *in vitro*, or myocardial contractility (i.e., fractional shortening), *in vivo*. Hyperdynamic systolic contraction, elevated force production, and/or prolonged periods of systolic tension strongly correlate with the development of pathological cardiac hypertrophy. However, our findings imply that altering the B-state and elevating basal tension throughout diastole may likewise be sufficient to independently cause hypertrophic remodeling, even if maximum systolic tension remains unchanged or is potentially reduced. Collectively, our multidisciplinary approach that combined *in vivo*, *in vitro*, and molecular-level modeling further emphasizes the role of impaired relaxation in HCM pathogenesis.

### Limitations of the study

(1) Our study relied on *Drosophila* models to confirm HCM-like myopathic responses, *in vivo*, to A331P mutant actin. Thus, the unique physiological properties and myocardial demands of flies must be considered as they may impact direct translational relevance for human HCM.
(2) *Drosophila* cardiac analysis was performed exclusively on female flies owing to larger body sizes and heart tube dimensions, relative to males. Thus, the influence of insect sex on A331P actin-associated cardiomyopathy was not investigated and limits generalizability of the results.
(3) Additionally, proper post-translational processing of recombinant human ACTCs was not confirmed. Sarcomeric actins, expressed via the baculoviruses/*Sf21* platform, are modified differently from those expressed in muscle. A lack of critical post-translational modifications, including N-terminal processing and methylation, may influence certain molecular properties. Nonetheless, both control and mutant ACTCs were generated under identical conditions, were likely processed equally post-translationally, and are thus directly comparable.
(4) Moreover, the lack of overt differences between our wild-type and A331P human F-actin reconstructions does not rule out the presence of minor structural perturbations that, potentially, might be overlooked at current resolution or by the current methodology.
(5) Finally, our work does not provide the relative contributions of electrostatic vs. vdW interactions to the energetics of F-actin-Tpm binding.

## Resource availability

### Lead contact

Further information and requests for resources and reagents should be directed to and will be fulfilled by the lead contact, Dr. Anthony Cammarato (acammar3@jhmi.edu).

### Materials availability

All unique/stable reagents generated in this study are available from the lead contact upon reasonable request.

### Data and code availability

• The authors declare that data supporting the findings of this study are available within the paper (and its supplementary information files) or are available from the corresponding authors upon reasonable request.
• This paper does not report original code.
• Any additional information required to reanalyze the data reported in this paper is available from the lead contact upon request.

## Acknowledgments

These studies were supported by a Heart and Stroke Foundation of Canada Grant-in-Aid G-18-0020424 (J.F.D.), by an American Heart Association/ DC Women's Board grant 22POS915659 (A.J.F.), and by 10.13039/100000002National Institutes of Health (NIH) grants HL036153 (W.L.) and HL124091 (A.C.).

## Author contributions

Conceptualization, A.C. and J.F.D.; methodology, A.C., J.F.D., M.H.D., W.L., and M.J.R.; investigation, M.H.D., E.D., M.J.R., M.C.V., A.M., K.C., A.J.F., and D.S.; writing – original draft, M.H.D., M.J.R., M.C.V., A.M., K.C., A.J.F., D.S., W.L., J.F.D., and A.C.; writing – review and editing, M.H.D., M.J.R., M.C.V., A.M., K.C., A.J.F., D.S., W.L., J.F.D., and A.C.; funding acquisition, A.C., J.F.D., and W.L.; resources, A.C., J.F.D., and W.L.; supervision, A.C., J.F.D., W.L., and M.J.R.

## Declaration of interests

The authors declare no competing interests.

## STAR★Methods

### Key resources table

**Table:** 

REAGENT or RESOURCE	SOURCE	IDENTIFIER
**Antibodies**		

Anti-α-Actinin	Sigma-Aldrich	Cat# A7732; RRID: AB_2221571
Goat anti-Mouse IgG Recombinant Secondary Antibody, Alexa Fluor 488	Thermo fisher	Cat# A28175; RRID: AB_2536161

**Bacterial and virus strains**		

Recombinant baculovirus	Bai et al., 2014^37^	

**Chemicals, peptides, and recombinant proteins**		

Alexa Fluor 568 Phalloidin	Thermo fisher	Cat# A12380
EGTA-AM	AAT Boquest	Cat# 99590-86-0
Blebbistatin	Cayman Chemical	Cat# 13186
WT-ACTC, A331P-ACTC	This paper	

**Critical commercial assays**		

QuikChange II XL by site-directed mutagenesis kit	Agilent Technologies	Cat# 200522
Quick-RNA microprep kit	Zymo Research Corp	Cat# 1050
QuantiTect Reverse Transcription Kit	Qiagen Inc.	Cat# 205311
TaqMan Fast Advanced Master Mix for qPCR	Thermo fisher	Cat# 4444556
Ni-NTA Spin Columns	Qiagen	Cat# 31014
DEAE Sepharose Fast Flow	GE Healthcare	Cat# 17070901

**Deposited data**		

EM density map, WT actin	This paper	EMD-44154
EM density map, A331P actin	This paper	EMD-44153
WT structure	This paper	9B3R
A331P structure	This paper	9B3Q

**Experimental models: Cell lines**		

Sf21 cells	Thermo Fisher	Cat# 11497013

**Experimental models: Organisms/strains**		

w[1118]	Genetic Services, Inc	
w[∗]; P{caryP, w[+], Act88F-WT}attP40; +	This paper	
w[∗]; P{caryP, w[+], Act88F-A331P}attP40; +	This paper	
w[∗]; P{caryP, w[+], Act88F-WT}attP40; Act88FKM88	This paper	
w[∗]; P{caryP, w[+], Act88F-A331P}attP40; Act88FKM88	This paper	
w[∗]; P{caryP, w[+], UASp::Act57B-WT}attP40	This paper	
w[∗]; P{caryP, w[+], UASp::Act57B-A331P}attP40	This paper	
*y[1] w[∗]; Mi{Trojan-GAL4.0}Hand[MI04106-TG4.0]*	Viswanathan et al., 2017^19^	

**Oligonucleotides**		

Act88F^A331P^ forward: ATCAAGATCATTC CGCCACCCGAGAGG	This paper	
Act88F^A331P^ reverse: CCTCTCGGGTGG CGGAATGATCTTGAT	This paper	
Act57B^A331P^ forward: ATCAAGATCATTC CTCCCCCAGAGCGC	This paper	
Act57B^A331P^ reverse: GCGCTCTGGGGG AGGAATGATCTTGAT	This paper	

**Recombinant DNA**		

*pUAS.Act57B*^*WT*^	Viswanathan et al., 2015^28^	
*pUAS.Act57B*^*A331P*^	This paper	
pattB *Act88F*^*WT*^	Viswanathan et al., 2017^19^	
pattB *Act88F*^*A331P*^	This paper	

**Software and algorithms**		

ImageJ		http://rsb.info.nih.gov/ij/
HCImage Live software	HCImage	https://hcimage.com/hcimage-overview/hcimage-live/
RELION 3.1.1	Zivanov et al., 2018^64^	www3.mrc-lmb.cam.ac.uk
SerialEM	Mastronarde, 2005^65^	bio3d.colorado.edu/SerialEM: www.nexperion.net/serialem
ChimeraX	Pettersen et al., 2021^66^	www.cgl.ucsf.edu/chimerax
Phenix	Liebschner et al., 2019^67^	phenix-online.org/
Coot	Emsley et al., 2004^68^	www2.mrc-lmb.cam.ac.uk/personal/pemsley/coot/
VMD	Humphrey et al., 1996^69^	www.ks.uiuc.edu/Research/vmd/

**Other**		

EVOS FL cell imaging system	Life Technologies	
Leica TCS SPE RGBV confocal microscope	Leica microsystems	
Hamamatsu Orca-Flash 2.8 digital camera	Hamamatsu	
Leica DM5000B DIC microscope	Leica microsystems	
Bio-Rad CFX96 Real-Time PCR Detection System	BioRad	
Zeiss Axiovert A.1 inverted microscope	Carl Zeiss	
Quantifoil 2/2 200 mesh grids	Quantifoil	
Vitrobot Mark IV	Thermo fisher	
ThermoFisher Scientific Titan Krios Electron Microscope	Thermo fisher	
Gatan K3 Direct Electron Detector	Thermo fisher	

### Experimental model and study participant details

#### Transgenes and transgenic *Drosophila melanogaster*

*Act88F*^*WT*^ (encoding UniProt ID: P83967) in the *pattB* vector, and *UAS-Act57B*^*WT*^ (encoding UniProt ID: P53501) housed in *pUASTattB*,^19^ were mutated to generate *Act88F*^*A331P*^ and *UAS-Act57B*^*A331P*^ constructs respectively, using the QuikChange II XL site-directed mutagenesis kit (Agilent Technologies) and custom primers:

Act88F^A331P^ (+) primer – 5′ ATCAAGATCATTCCGCCACCCGAGAGG 3′

Act88F^A331P^ (-) primer – 5′ CCTCTCGGGTGGCGGAATGATCTTGAT 3′

Act57B^A331P^ (+) primer – 5′ ATCAAGATCATTCCTCCCCCAGAGCGC 3′

Act57B^A331P^ (-) primer – 5′ GCGCTCTGGGGGAGGAATGATCTTGAT 3′

Transgenic *Drosophila* were generated as described in Viswanathan et al.^19^^,^^28^ The PhiC31 integrase system was employed to ensure all transgenes were inserted into an identical, predetermined location (*attp40*) on chromosome two.^70^ Because the transgenes’ cytolocation is defined, and all constructs integrated into the same locus, any phenotypic differences in control vs. mutant flies were directly attributable to the A331P mutation.

#### *Drosophila* stocks and husbandry

*Drosophila* stocks used in this study are listed in Table 1. Flies were raised and crosses carried out at 25°C on standard cornmeal-yeast-sucrose-agar medium. *Act88F* transgenic *Drosophila*, designated *WT*^▼^ or *A331P*^▼^, possess two endogenous *Act88F* genes on the third chromosome, and two transgenic copies (*Act88F*^*WT*^ or *Act88F*^*A331P*^) inserted into the second. To generate animals with one transgenic copy and one endogenous *Act88F* gene, *WT*^*▼*^ or *A331P*^*▼*^ were crossed with *Act88F*^*KM88*^ (a viable *Act88F-null* line) to yield *WT*^▼^/+; *Act88F-null*/+ and heterozygous *A331P*^▼^/+; *Act88F-null*/+ animals. Employing standard mating schemes, publicly available balancer stocks, our transgenic lines, and *Act88F*-nulls, we also produced *WT*^▼^; *Act88F-null*/+ and *A331P*^▼^; *Act88F-null*/+ flies. Subsequent crosses established stable lines homozygous for transgenic actin, devoid of endogenous *Act88F*, referred to as *WT*^▼^; *Act88F-null* and *A331P*^▼^; *Act88F-null*.

The *Gal4-UAS* system was utilized to achieve heart-restricted transgene expression.^71^ Female *Hand*^*4.2*^*-Gal4* flies (expressing *S. cerevisiae Gal4 -* UniProt ID: P04386, under the control of the *Drosophila* cardiac promotor *Hand* - UniProt ID: Q9VL05) were crossed with males containing the *UAS-Act57B*^*WT/A331P*^ constructs for cardiac specific expression of transgenic actin.

#### Recombinant human ACTC

Human recombinant wild-type and A331P ACTCs (UniProt ID: P68032) were expressed in *Sf*21 cells with recombinant baculoviruses, produced using the *pAcUW2Bmod* plasmid.^72^ The actins were purified via affinity chromatography,^73^ binding actin proteins in cell lysates to His-tagged gelsolin G4–6 in the presence of Ca^2+^, and eluting the ACTC proteins in a buffer containing EGTA (ethylene glycol- bis(b-aminoethyl ether)-N,N,N=,N=-tetraacetic acid). Briefly, cells expressing ACTC proteins were disrupted by vortexing with glass beads in lysis buffer containing protease inhibitors and Ca^2+^ to which His-tagged G4-6 was added to bind the actin. The His-tagged G4-6:actin complexes were bound to a Ni-NTA column and the actin released with a buffer containing EGTA and magnesium. ACTC-containing fractions were dialyzed into a storage buffer, kept on ice and used within one week of purification. SDS-PAGE was used to verify the presence of pure ACTC and the progress of purification.

#### *In vitro* motility assays

*In vitro* motility (IVM) assays were performed with rabbit skeletal muscle HMM (UniProt ID: Q28641) and thin filaments reconstituted using purified recombinant human cardiac actin and bovine ventricular Tn-Tpm (UniProt IDs: P08057, P63315, P13789, and Q5KR48), or with full-length, rabbit skeletal myosin and recombinant human F-actin ± bovine Tpm. Bovine cardiac actin (UniProt ID: Q3ZC07) was used to non-reversibly block inactive myosin adhered to the motility flow cell surface.

### Method details

#### Flight tests

Flight tests were performed on two-day-old male and female *Drosophila* as described earlier.^74^ Flies (*n* =173-657), were released into the center of a plexiglass chamber and flight direction tracked (scored 6 for upward, 4 for horizontal, 2 for downward, or 0 for no flight).

#### Indirect flight muscle and myofibril imaging

IFM and myofibril imaging of two-day-old male and female *Drosophila* were performed as described previously.^19^^,^^28^^,^^29^ In brief, paraformaldehyde fixed fly thoraces were flash frozen and bisected along the sagittal plane using a sharp razor blade. Hemi-thoraces were stained with primary mouse anti-α-actinin and secondary anti-mouse Alexa488-cojugated antibodies along with Alexa568-phalloidin, rinsed and the DLMs imaged on an EVOS® FL cell imaging system (Life Technologies) at 4X magnification. For imaging IFM myofibrils, DLMs were carefully removed from the hemi-thoraces, and myofibrils gently teased apart with fine forceps and mounted in Vectashield directly between a glass slide and a coverslip. The samples were imaged on a Leica TCS SPE RGBV confocal microscope at 100X magnification and captured at 3X zoom.

#### *Drosophila in situ* cardiac analysis

One-, three-, five-, and seven-week-old, female *Hand-Gal4 > UAS-Act57B*^*WT*^ or *UAS-Act57B*^*A331P*^ *Drosophila* hearts were exposed under artificial hemolymph (AH).^19^^,^^40^^,^^75^ Female flies were selected for cardiac studies due to sex-related differences in body size and cardiac dimensions.^41^ High-speed videos (∼130 fps) of actively beating hearts were recorded using a Hamamatsu Orca-Flash 2.8 digital camera on a Leica DM5000B DIC microscope fitted with a 10X immersion lens (0.30 N.A.). Indices of cardiac performance were extracted using the Semi-automated Optical Heartbeat Analysis (SOHA) program.^19^^,^^40^^,^^46^

#### Measurement of EGTA/EGTA,AM- and blebbistatin-induced changes in cardiac dimensions

*In situ* semi-intact hearts of three-week-old *Hand > UAS-Act57B*^*WT*^ or *Act57B*^*A331P*^ females (*n* = 31 and 22, respectively) were imaged and filmed as described by Viswanathan et al.^19^^,^^41^ using a 20X (0.50 N.A.) immersion lens. Beating hearts were recorded for 10 sec at different focal depths to distinctly resolve edges along the length of each tube. Hearts were subsequently incubated in AH supplemented with 10 mM EGTA and 100 μM EGTA,AM (AAT Bioquest), with the latter being a cell-permeant EGTA precursor that can be passively loaded into cells to generate intracellular EGTA, for 30 min to chelate extra and intracellular Ca^2+^. The motionless hearts were recorded as before, and then incubated for another 30 mins after addition of 100 μM blebbistatin (Cayman chemical). The hearts were re-imaged for a final time. Videos were analyzed using HCImage Live software and three diameter measurements were made at identical locations along each heart from images obtained during diastole and the two treatment conditions, and then averaged.

#### Quantitative polymerase chain reaction

Total RNA was isolated from three-week-old *Hand-Gal4>UAS-Act57B*^*WT*^ and *Hand-Gal4>UAS-Act57B*^*A331P*^ dissected hearts using the Quick-RNA microprep kit (Zymo Research Corp.). 10 ng of total RNA was used in the reverse transcription reaction to generate cDNA using the Qiagen QuantiTect Reverse Transcription Kit (Qiagen Inc.). Quantitative polymerase chain reaction was performed on a Bio-Rad CFX96 Real-Time PCR Detection System. Taqman primers (ThermoFisher Scientific) targeting universally-transcribed regions of the L-type Ca^2+^ channel (*Ca-α1D*; Dm01807733), ryanodine receptor (*RyR*; Dm01842311), sarcoplasmic reticulum Ca^2+^-ATPase (*SERCA*; Dm01820194), Na/Ca exchanger (*Calx*; Dm02136145), inositol-3-phosphate receptor (*Itp-r83A*; Dm02147941) and GAPDH (*Gapdh1*; Dm01843827) were employed. Four biological replicates (15 hearts per sample) per gene, with three technical replicates of each sample were performed and averaged.

#### *In vitro* motility assays

Standard *in vitro* motility (IVM) assays were performed as described previously with minor modifications.^19^^,^^76^ Briefly, flow cells were created by adhering nitrocellulose-coated glass coverslips to microscope slides with double sided tape. HMM was produced from full-length myosin, purified from rabbit soleus muscle. To remove any inactive S1 heads, HMM was incubated with F-actin, followed by centrifugation and addition of ATP to release functional HMM. The process was repeated three times to obtain active HMM. IVM was performed by first binding 125 μg/mL of active HMM to the nitrocellulose flow cell. The surface was subsequently blocked with 1 mg/ml BSA, followed by addition of 10,000x diluted rhodamine-phalloidin-labeled F-actin, 300 nM each of Tn and Tpm, and finally a motility buffer of a particular pCa. Videos were captured for 45 sec each using a digital video camera mounted on a Zeiss Axiovert A.1 inverted microscope (Carl Zeiss AG, Oberkochen, Germany) using a Texas red filter. Three videos were typically collected for each of the pCa values.

To quantitate the velocity of motile filaments at pCa 4.5, 5, 8, and 10, videos were imported into ImageJ and contrast corrected. Up to ten medium-sized filaments were chosen based on directed movement; if fewer than ten filaments were motile in a given video, only those that showed movement were chosen. The distance traveled by a single filament was assessed by measuring the start and end point of a filament using the line tool, over a period of ten frames. Velocity data was obtained using:

$$V\;\left(\frac{\mu m}{s}\right)=\frac{\left(\frac{D}{F_{E}-F_{s}}\right)\times 89.46\;\frac{nm}{pixel}\times 2.48\;\frac{frames}{s}}{1000\;\frac{nm}{\mu m}}$$

where *D* is the distance traveled in pixels, *F*_*E*_ is the end frame number and *F*_*S*_ is the start frame number.

Human wild-type and A331P F-actin motility was measured, in the presence and absence of Tpm, via a multiplexed *in vitro* motility assay using full-length, rabbit skeletal myosin at a sub-saturating concentration of 35 μg/mL. Multiplexing (i.e., simultaneous recording of differentially-labeled actin filaments) minimizes variability introduced by differences in microenvironments within a given flow cell and helps resolve the most minor molecular discrepancies between filament types.

Here, full-length, active myosin was allowed to bind to a nitrocellulose-coated cover slip, in a flow cell, for 1 min. The surface was subsequently blocked with 1 mg/ml BSA. Unlabeled bovine cardiac F-actin was introduced to the flow cell to non-reversibly block any remaining inactive myosin now adsorbed to the motility surface. ∼5 nM Alexa488-phalloidin-labeled wild-type and ∼5 nM Alexa568-phalloidin-labeled A331P ACTC were added to the flow cell to bind to the enzymatically active myosin. Finally, motility buffer (25 mM KCl, 4 mM MgCl_2_, 1mM EGTA, 25 mM imidazole, 10 mM DTT, 1 mM ATP, 2 mM dextrose, 17 units/ml glucose oxidase, 125 units/ml catalase, and 0.5% methyl cellulose; pH 7.2) was added and the displacement of the different actin filaments was concurrently visualized in their respective channels. To assess the effects of the binding of Tpm on motility of the F-actins, 300nM bovine cardiac Tpm in actin buffer (25 mM KCl, 4 mM MgCl_2_, 1 mM EGTA, and 25 mM imidazole; pH 7.2) was added to the flow cells post introduction of the phalloidin-labelled F-actins, and incubated for 3 min to promote F-actin-Tpm binding. F-actin motility was analyzed in the presence of motility buffer containing 140 nM Tpm. Time lapse images were obtained at 10 fps on an Olympus IX81 spinning disk confocal microscope with two iXon Ultra EMCCD cameras facilitating dual color imaging, simultaneously.

The resultant tiff images were processed in ImageJ for background correction and to enhance contrast for better F-actin visibility and detection. The TrackMate plugin^77^ was used to track and obtain the X-Y coordinates of each mobile/ immobile filament. A MATLAB script was used to apply the method described by Duno-Miranda et al^78^ to calculate the Mean Squared Displacement (MSD) of each filament across a range of time intervals such that

$$MSD\left(n\Delta t\right)=\frac{1}{N-n}\;\sum_{i=1}^{N-n}\left[{\left(x_{i+n}-x_{i}\right)}^{2}+{\left(y_{i+n}-y_{i}\right)}^{2}\right],n=1,\ldots ,N/4$$

where *N* is the total number of frames measured and *n* is the number of frames in a given interval. In brief, velocity was calculated as the slope of the MSD vs time interval (*n*Δt) relationship, and the slope of the log(MSD) vs log(*n*Δt) relationship provided the diffusive exponent (α), where α > 1 indicates directed motion. Thresholding α at 1 filtered out all non-mobile filaments allowing the measurement of velocities of only mobile filaments. This marks a single experiment. At least 3 such experiments were performed with 5 ROIs imaged per flow cell chamber, with at least 20 filaments/ROI. It is noteworthy that each flow cell was partitioned into two separate chambers using a strip of double-stick tape for parallel assessment of the F-actins in the presence and absence of Tpm to further nullify variability.

#### Cryo-EM data collection

Wild-type and mutant human actin samples were prepared at the Johns Hopkins Beckman Cryo-EM Center under the following conditions. Quantifoil 2/2 grids were glow discharged for 30s with a current of 15 mAmps using a Pelco Easiglow. The grids were allowed to rest at least 15 min in atmosphere (18^o^C and 60% humidity). 4 μLs of sample was applied to a grid, blotted, and then plunge frozen in liquid ethane using a Vitrobot Mark IV (Thermo Fisher Scientific). The Vitrobot was set to 4^o^C and 100% humidity. 3 grids each were prepared for A331P and wild-type F-actin. The blot times varied from 2.5s to 4.5s.

Data were collected at the Johns Hopkins Beckman Cryo-EM Center on a Thermo Fisher Titan Krios G3i set to 22500x nominal magnification and 300 kV with a Cs of 2.7. A Gatan K3 direct detector was used to collect 1594 A331P images and 5035 wild-type images in super resolution mode under the following conditions: 4.0s exposure time, 60 electrons/Å2 total dose, 15 electrons/Å2/s, 100 total frames resulting in 0.6 electrons per frame, and a nominal defocus varying between 0.8 and 3.0 μm. This pixel size was 0.529 Å/pixel. SerialEM software was used for data collection.^65^

#### Image processing and 3D reconstruction

For both the wild-type and A331P mutant filaments, image reconstruction was carried out using RELION 3.1.1. Micrographs were first motion corrected using MotionCor2^79^ indicating a binning of 2x and the contrast transfer function (CTF) was estimated using CtfFind4.1.13.^80^ After the preprocessing steps, approximately 250 filaments were manually picked in the RELION GUI and divided into 300 Å (280 pixels) overlapping segments with an inter-box distance of 27.5 Å. The manually picked segments were extracted and subjected to 2D classification to generate templates for autopicking. Autopicking procedures in RELION were optimized on a test set of 10 micrographs and then performed over the full datasets. In total, 1,291,946 segments were picked for the wild-type dataset and 234,263 segments were picked for the A331P dataset. Following autopicking, the segments were binned 3x (new pixel size of 4.232 Å) during extraction and run through four iterations of 2D classification to discard auto-picked false positives, damaged, and ice-contaminated segments. After 2D classification, the segments were re-extracted at the original 1.058 Å pixel size for further curation in 3D classification. In total, the final particle stacks used for refinement after classification were 133,304 for wild-type and 140,732 for the A331P mutant. Refinements of both filaments were completed in the same manner. In the first refinement, a 105 Å-diameter featureless cylinder was used as a reference. After the initial refinements, two rounds of CTF refinement paired with Bayesian polishing yielded final maps with resolutions at 3.5 Å (wild-type) and 3.6 Å (A331P). To generate the final sharpened maps, the RELION post-processing step was used, utilizing a mask that extended to 40% of the filament length. Final, improved maps to aid in model building were then generated using the PHENIX density modification procedure.^67^

#### Model building and refinement

To generate the initial wild-type actin model, an individual actin subunit was extracted from PBD: 7UTL and the human sequence was incorporated using SWISS-MODEL.^81^^,^^82^ The A331P actin was created by manually incorporating proline at position 331 in Coot.^83^ After initial models were created, 3 subunits were fit into the final map using the ChimeraX^34^ fit in map tool. After initial fitting, both models were subjected to rounds of manual rebuilding in Coot^83^ and real-space refinement in Phenix.^67^^,^^84^ Secondary structure, rotamer, and Ramachandran restraints were used to produce the best geometries. Final models were validated in Phenix and model statistics are provided in Table S1.

#### Molecular dynamics simulations of wild-type and A331P ACTC

To test the dynamics of human wild-type and A331P ACTC filaments, we initially performed simulations of bare F-actins. Single protomers from each cryo-EM-derived structure were expanded using F-actin symmetry to create a 28-monomer filament. The extension allowed the periodic boundary conditions used in explicit solvent simulations to also extend the polymer along the z-axis in an essentially infinite filament. After the system was solvated, it was neutralized by addition of sodium chloride atoms to a final concentration of 0.15 M and magnesium chloride to a concentration of 3 mM.

This system was then minimized in NAMD^85^ first by fixing all protein atoms to relax the solvent molecules and then in a constrained minimization of all atoms to their starting coordinates with slow release of these constraints in 5 stages, with 2000 steps of conjugate gradient minimization each. The system was then heated at constant volume to 300 K with constraints to the minimized coordinates. These constraints were then removed in stages, over the next 1 ns of simulation time, under constant pressure (1 atm). Production runs were carried out over the next 30-40 ns.

In addition to testing the dynamics of bare F-actins, we also ran simulations in the presence of regulatory components. Here, published thin filament models in low and high Ca^2+^ (PDB: 7UTL, 7UTI respectively) were extended using filament symmetry to comprise a total of 8 Tpm chains (residues 1-284), 28 actin monomers, and 4 total troponin complexes.^14^ As with bare F-actin, the extension of this system allowed the periodic boundary conditions to also extend the actin-Tpm filament to create an essentially infinite filament. The high and low Ca^2+^ models were both included in a single filament with one of each model included on opposite sides of the long-pitch helix of F-actin. Once constructed, the actin monomers in the PDB coordinates were replaced with the wild-type or A331P actin structure as determined by cryo-EM, and the troponin chains were removed with the exception of troponin T residues 89-151 and troponin I residues 137-210 (low Ca^2+^ state only). A single side chain in the A331P actin system was moved to relieve a poor contact formed during building that would not be resolved by minimization. When solvating the system, four additional actin monomers and Tpm chains were added to either end of the filament to explicitly define the periodic copies used in later calculations. This step prevented clashes between the solvent molecules added in VMD^69^ and periodic actin and Tpm residues from the other end of the model system. Finally, the system was solvated and neutralized by the addition of sodium chloride atoms to 0.15 M and magnesium chloride to 3 mM.

After the system was built, it was minimized in NAMD^85^ following the same strategy as outlined above for the bare F-actin simulations. Once again, production runs were executed over the next 30-40 ns. Analysis of the interaction energy between Tpm and actin was performed using the NAMDEnergy plugin in VMD. Determination of the superhelical axis of the Tpm coiled coils on each frame of the simulations was performed using the program TWISTER.^52^ The RMSF of the resulting superhelical positions was then calculated in VMD.^69^

### Quantification and statistical analyses

All statistical analyses were performed using GraphPad Prism 10 software. Data are presented as mean ± SEM or SD (as indicated in Figure legends). *n* values and definitions are provided in Figure legends. Significant differences in flight ability, among > 2 genotypes, were assessed using the Kruskal–Wallis test. Large sample sizes alleviated concerns related to flight score heterogeneity. Significant differences in cardiac parameters between genotype and age, and interaction effects, were evaluated using two-way ANOVAs. Here, when measured values were not normally distributed, data were logarithmically transformed before significance was assessed. The effects of EGTA/ EGTA,AM and blebbistatin treatment on cardiac dimensions were evaluated using repeated measures ANOVAs followed by Tukey’s multiple comparison tests of the matched groups. Unpaired t-tests were then used to distinguish significant differences in the cardiac responses to EGTA, EGTA.AM and blebbistatin treatment between the genotypes. Significant differences in *Drosophila* cardiac Ca^2+^-handling gene expression, *in vitro* sliding speeds, and computationally derived F-actin-Tpm vdW interaction energies were likewise determined using unpaired t-tests (with Welch’s correction, where noted in legends), and flight ability between 2 groups, via the Mann-Whitney U test.

## Supplemental information

Document S1. Figures S1–S16, Table S1, and DataS1
