# Supplementary material for: The hypertrophic cardiomyopathy-associated A331P actin variant enhances basal contractile activity and elicits resting muscle dysfunction
Source: iScience. 2025 Jan 16;28(2):111816. doi: 10.1016/j.isci.2025.111816 (PMC11841076; doi:10.1016/j.isci.2025.111816)
Supplement: Document S1. Figures S1–S16, Table S1, and DataS1 [file mmc1.pdf]

iScience, Volume *28*

Supplemental information

The hypertrophic cardiomyopathy-associated A331P actin variant enhances basal contractile activity and elicits resting muscle dysfunction

Matthew H. Doran, Michael J. Rynkiewicz, Evan Despond, Meera C. Viswanathan, Aditi Madan, Kripa Chitre, Axel J. Fenwick, Duncan Sousa, William Lehman, John F. Dawson, and Anthony Cammarato

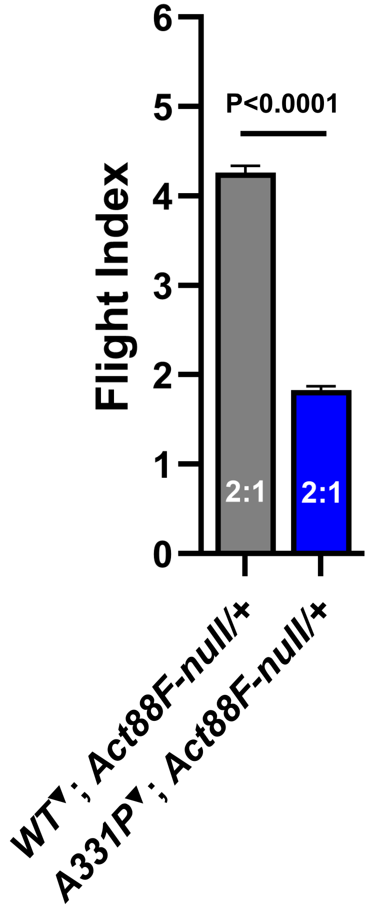

Supplementary Figure 1: Flight indices of transgenic *Drosophila*. *A331P▼/+; Act88F-null/+* heterozygous animals (with one transgene and one endogenous *Act88F* allele) displayed a significantly lower flight index vs. *WT▼/+; Act88F-null/+* flies (Figure 2A). Increasing the transgenic:endogenous allele number to 2:1 further depressed flight among both lines, with *A331P▼; Act88F-null/+* exhibiting significantly worse flight ability when compared to *WT▼; Act88F-null/+*. Data are presented as mean ± SEM. Significant differences in flight ability were determined by the Mann–Whitney U test (n=386-459 flies/genotype).

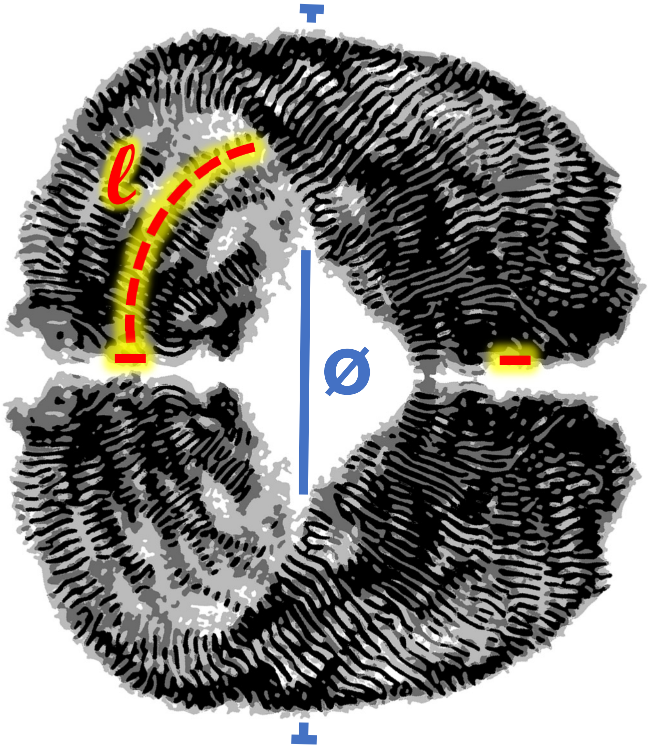

Supplementary Figure 2: The fly "heart" is composed of a single layer of conjoined cardiomyocytes that form a linear tube coined the "dorsal vessel". Given the structural properties inherent to the fly's heart, we devised a protocol to decipher the mechanistic basis of diastolic restriction. Since the distance across the dorsal vessel's, lumen-forming, conjoined cardiomyocytes (ø) is directly proportional to cell length (l), which in turn is directly proportional to the degree of contractile activity, cardiac tube diameter can serve as a proxy measure of Tpm regulatory position along thin filaments and its myosin blocking capacity. Image modified from https://doi.org/10.1016/j.yjmcc.2018.04.010.

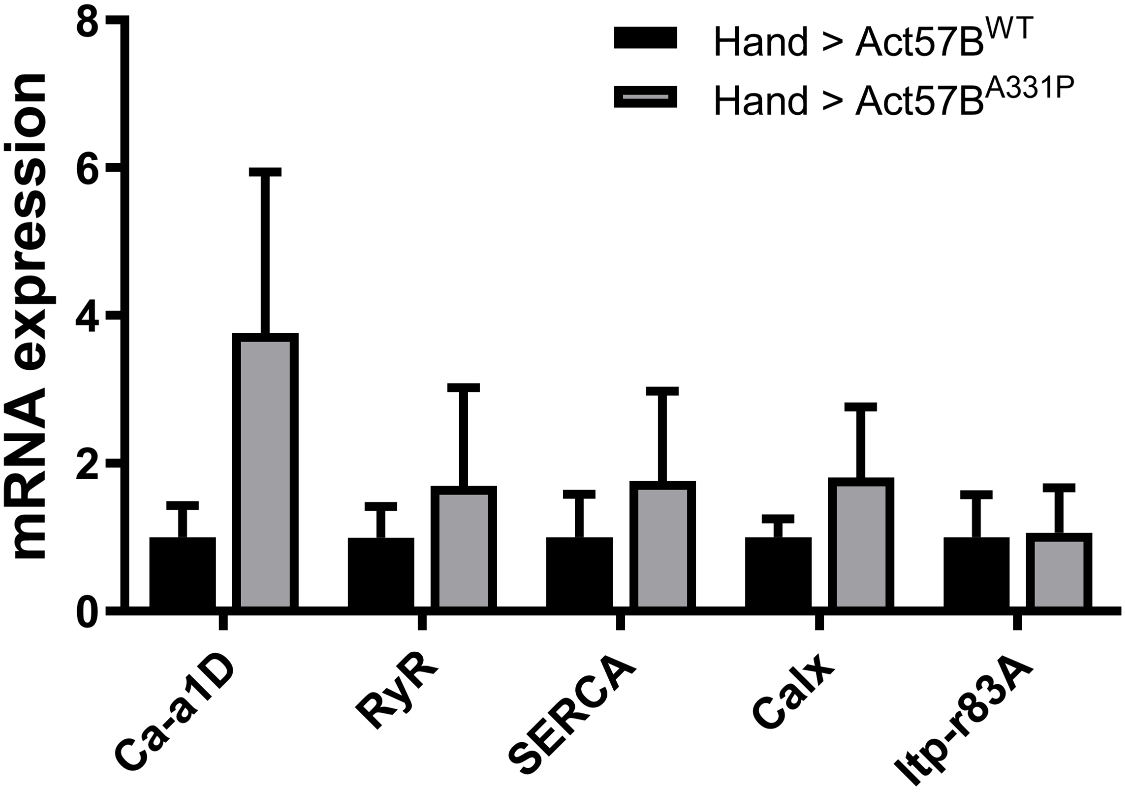

Supplementary Figure 3: A331P actin does not alter Ca^{2+}-handling gene expression. Quantitative polymerase chain reaction measurements of L-type Ca^{2+} channel, ryanodine receptor, sarcoplasmic reticulum Ca^{2+}-ATPase, Na/Ca exchanger, and inositol-3-phosphate receptor transcript levels, in hearts of *Hand-Gal4*>UAS-*Act57B*^{A331P} relative to *Hand-Gal4*>UAS-*Act57B*^{WT} control. Data are presented as mean ± SEM. Two-tailed unpaired t-tests revealed no significant differences in the expression of Ca^{2+}-handling genes between *Hand-Gal4*>UAS-*Act57B*^{WT} and *Hand-Gal4*>UAS-*Act57B*^{A331P} hearts. Transcript levels displayed are normalized to GAPDH transcript amounts for four biological replicates (15 hearts per biological replicate) with three technical replicates each.

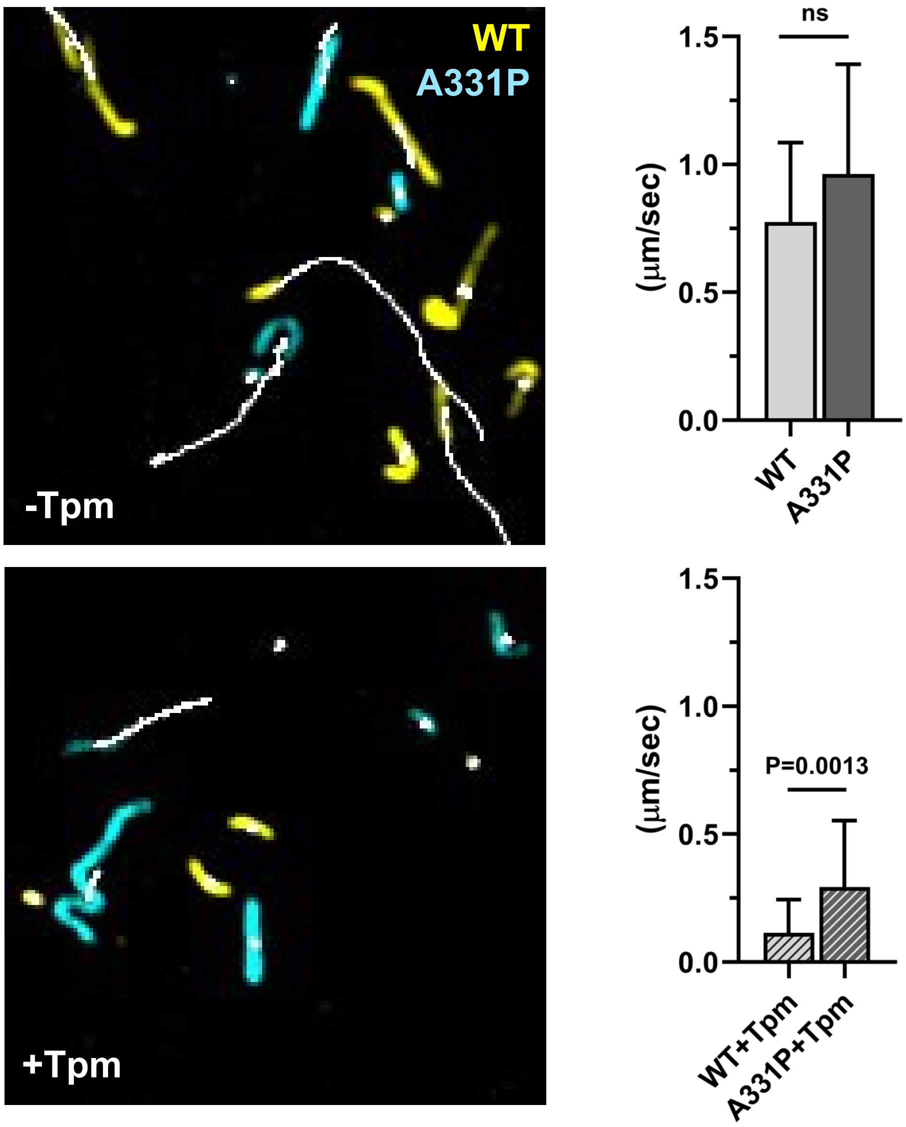

Supplementary Figure 4: Multiplex *in vitro* motility results. To determine if the A331P mutation impairs Tpm's intrinsic ability to block acto-myosin binding, we compared sliding velocities of human wild-type and mutant F-actin, under sub-saturating myosin conditions (i.e., 35 μg/ml), ±Tpm. To facilitate resolving even minor discrepancies, we differentially labelled each recombinant F-actin type with a unique fluorophore and simultaneously assessed sliding velocities for wild-type (yellow) vs. A331P (cyan) filaments, directly, over the same bed of myosin (also see Table 1). Filament sliding was more pronounced in the absence of Tpm (Top, left) than in its presence (Bottom, left) as illustrated by filament motion tracks (white). No significant difference was found in mean filament sliding velocity between wild-type and A331P human F-actins (Top, right). However, in the presence of Tpm, A331P filaments were propelled at a significantly higher average velocity relative to wildtype F-actin-Tpm (Bottom, right). Data are presented as mean ± SD. Significance was assessed via unpaired t-tests (n ≥ 100 filaments/condition).

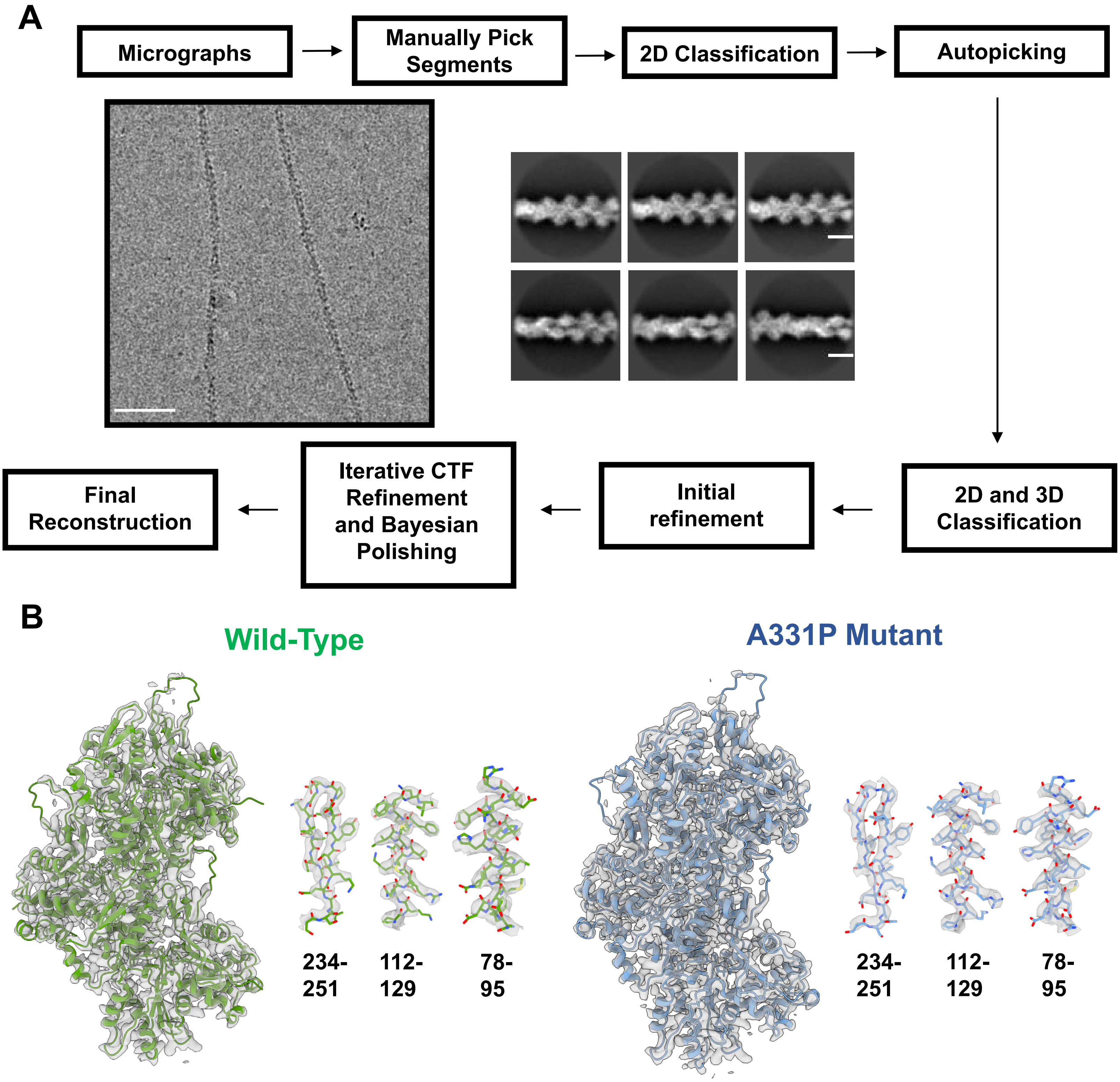

Supplementary Figure 5: A. Chart outlining the workflow that led to the cryo-EM reconstructions of recombinant human cardiac actin, which is described in detail in the Methods section in the main text. Representative micrograph shows the ultrastructure of the actin filaments. Both the WT and mutant filaments displayed similar overall structure. Reference-free 2D classes of the filaments reached resolutions sufficient to identify secondary structure, and are displayed beside the micrograph. **B.** Representative cryo-EM map-to-model fits. The final cryo-EM densities are displayed as transparent, while the fitted atomic models are shown as cartoons. Both reconstructions have adequate resolution to reliably place side-chains.

A

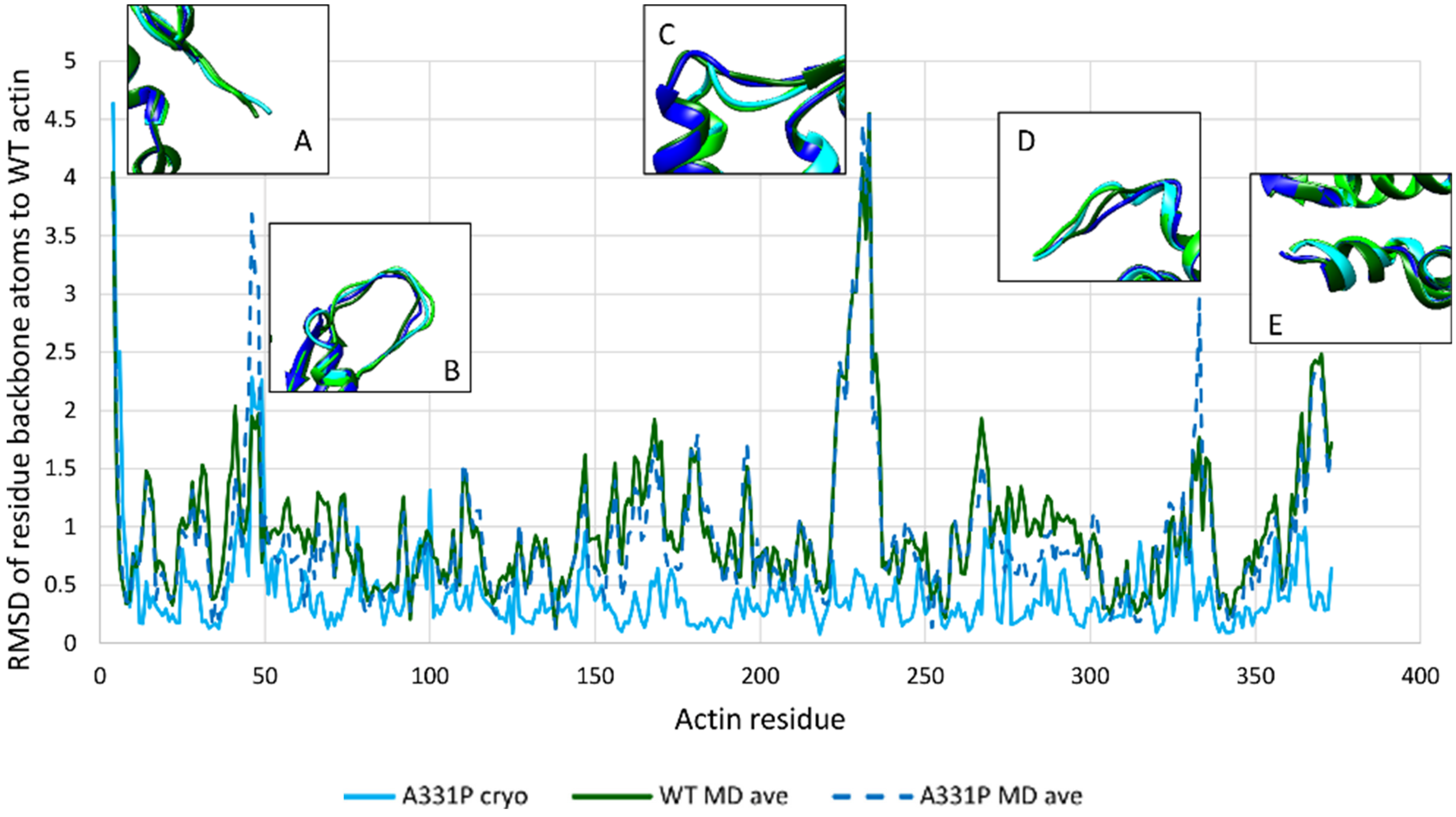

B

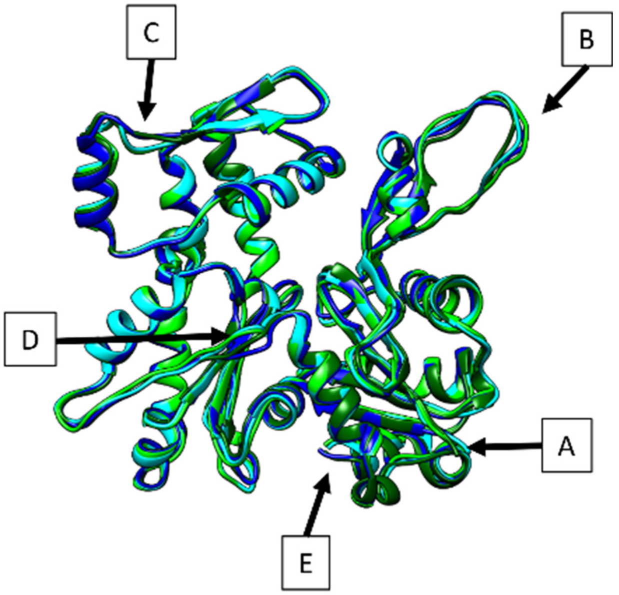

Supplementary Figure 6: A. Analysis of residue-by-residue RMSD values, comparing wild-type to A331P cryo-EM-derived protomer structures (cyan line) and wild-type cryo-EM- to average MD-generated wild-type (dark green line) and MD-generated A331P (blue, dotted line) actin models. Structures were first aligned to the cryo-EM-derived wild-type model using all backbone atoms. RMSD values were then calculated and graphed on a per residue basis to illustrate conservation of structure in both cryo-EM and MD. Insets show local actin structure where the RMSD between the wild-type and MD models diverge at the N-terminus (A), D-loop (B), subdomain-4 linker (C), 320-334 surface strand (D), and C-terminus (E). **B.** Ribbon diagrams revealing the loci of the insets in panel A, in the context of an actin monomer. Wild-type actin, from the cryo-EM-derived model, is displayed in pale green, while other models are colored as in panel A.

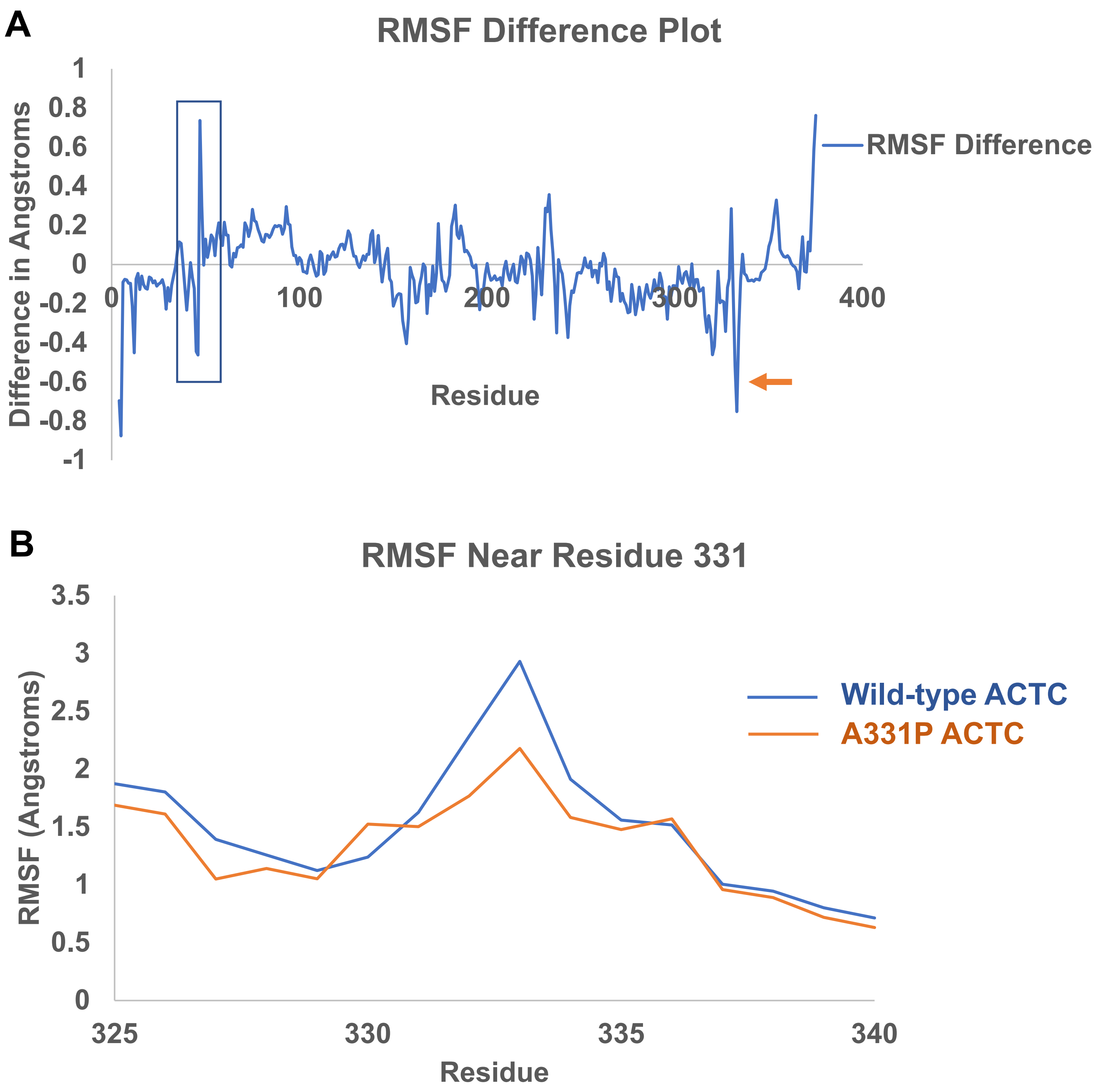

Supplementary Figure 7: A. RMSF difference plot (A331P RMSF - WT RMSF) from the actin-alone MD simulations (Figure 4C) shows the regions of the two actins that have divergent mobility. Although there are several regions that diverge, the largest differences come along the 325-334 surface strand close to the A331P mutational locus (orange arrow), and the D-loop (encased by a rectangle). **B.** Enlarged representation of RMSF values calculated around residue 331 for wild-type (blue) and A331P (orange) actins over the course of the actin-alone simulation (Figure 4C). Note that although the surrounding residues appear unchanged, the region around position P331 exhibits a significant dip in fluctuation.

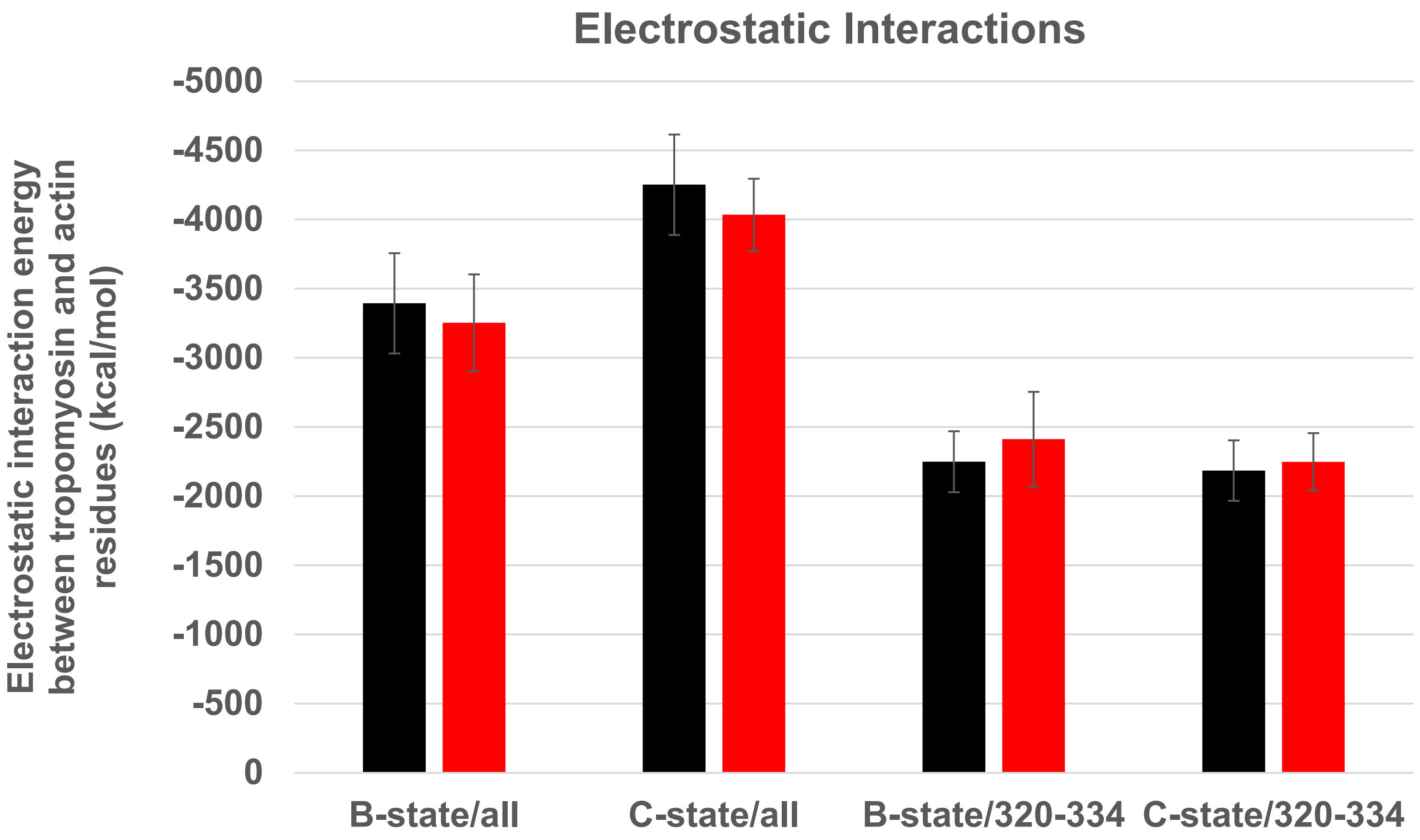

Supplementary Figure 8: Electrostatic interaction energy between Tpm (B-state or C-state) and wild-type (black) or A331P (red) F-actin (including all residues or residues 320-334) was measured over the final 20-30 ns of MD simulation time (135-200 frames in total). The bar graphs represent the average values ± SD.

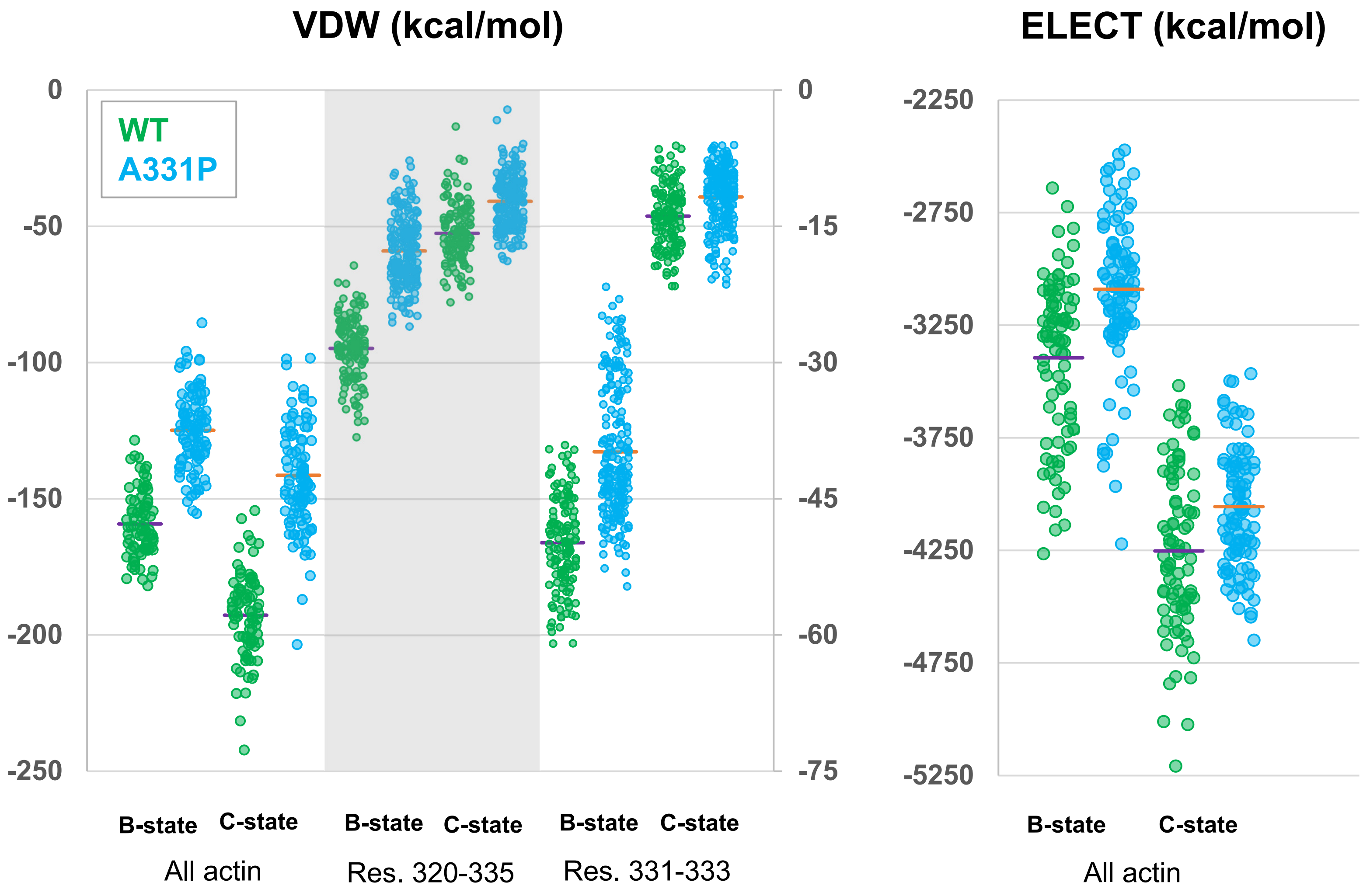

Supplementary Figure 9: Jitter plots of van der Waal's (left) and electrostatic (right) interaction energy between Tpm and actin. Individual values for wild-type (WT; green) and A331P (cyan) are plotted for 100 frames, showing the distribution of values over the last 20 ns of MD simulation. The average values are shown with purple (WT) and orange (A331P) lines. van der Waal's interactions are considered for Tpm and the entire actin molecule, as well as the smaller fragments indicated. The left VDW y-axis corresponds to the energy values (kcal/mol) plotted for "All actin" and Res. 320-335, while the right y-axis corresponds to the values for Res. 331-333 only. The van der Waal's interactions are reduced in the A331P mutant simulations.

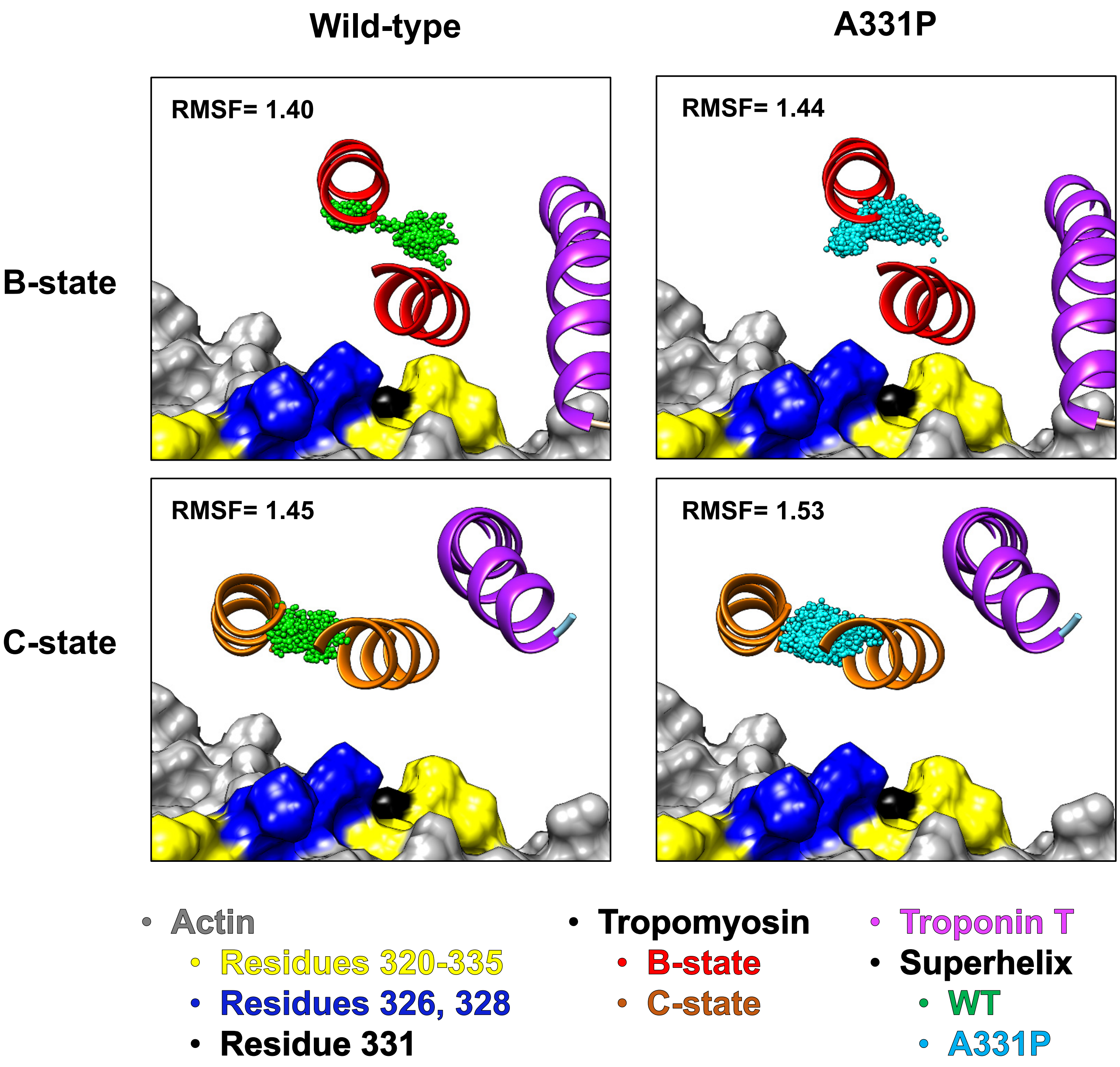

Supplementary Figure 10: PR1 Tpm superhelix positions, Tpm-Leu13. Shown is the distribution of the superhelix positions at Tpm residue 13 during the last 10 ns of MD simulation. 1000 frames for wild-type (green dots) and A331P (cyan dots) are shown, revealing the dynamic movement of the Tpm coiled coil in the vicinity of the A331P mutation site. Actin is rendered as a surface colored blue (residues Lys326 and Lys328), yellow (Pro333), black (Ala331 or Pro331), and gray (all other actin residues). Tpm in its initial position is shown as red (B-state) and orange (C-state) ribbons. TnT is shown as a purple ribbon. Each frame examined was aligned to the initial actin coordinates underlying the Tpm residue of interest. This was done to account for any movements of the actin monomers during the simulation and to superimpose the results from the two B- or C-state Tpm dimers in the system.

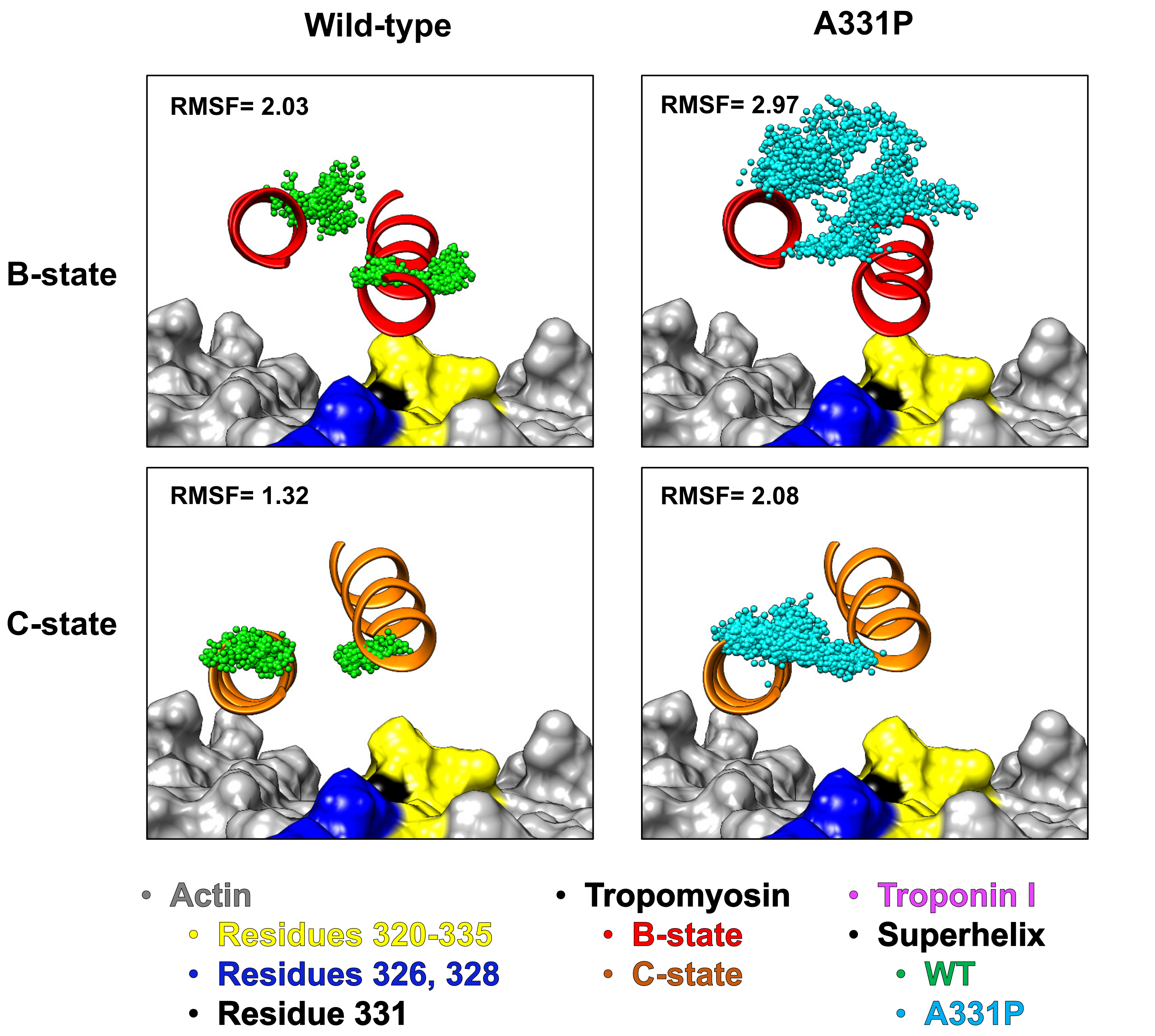

Supplementary Figure 11: PR2 Tpm superhelix positions, Tpm-Leu51. Shown is the distribution of the superhelix positions at Tpm residue 51 during the last 10 ns of MD simulation. 1000 frames for wild-type (green dots) and A331P (cyan dots) are shown, revealing the dynamic movement of the Tpm coiled coil in the vicinity of the A331P mutation site. Actin is rendered as a surface colored blue (residues Lys326 and Lys328), yellow (Pro333), black (Ala331 or Pro331), and gray (all other actin residues). Tpm in its initial position is shown as red (B-state) and orange (C-state) ribbons. TnI is shown as a magenta ribbon. Each frame examined was aligned to the initial actin coordinates underlying the Tpm residue of interest. This was done to account for any movements of the actin monomers during the simulation and to superimpose the results from the two B- or C-state Tpm dimers in the system.

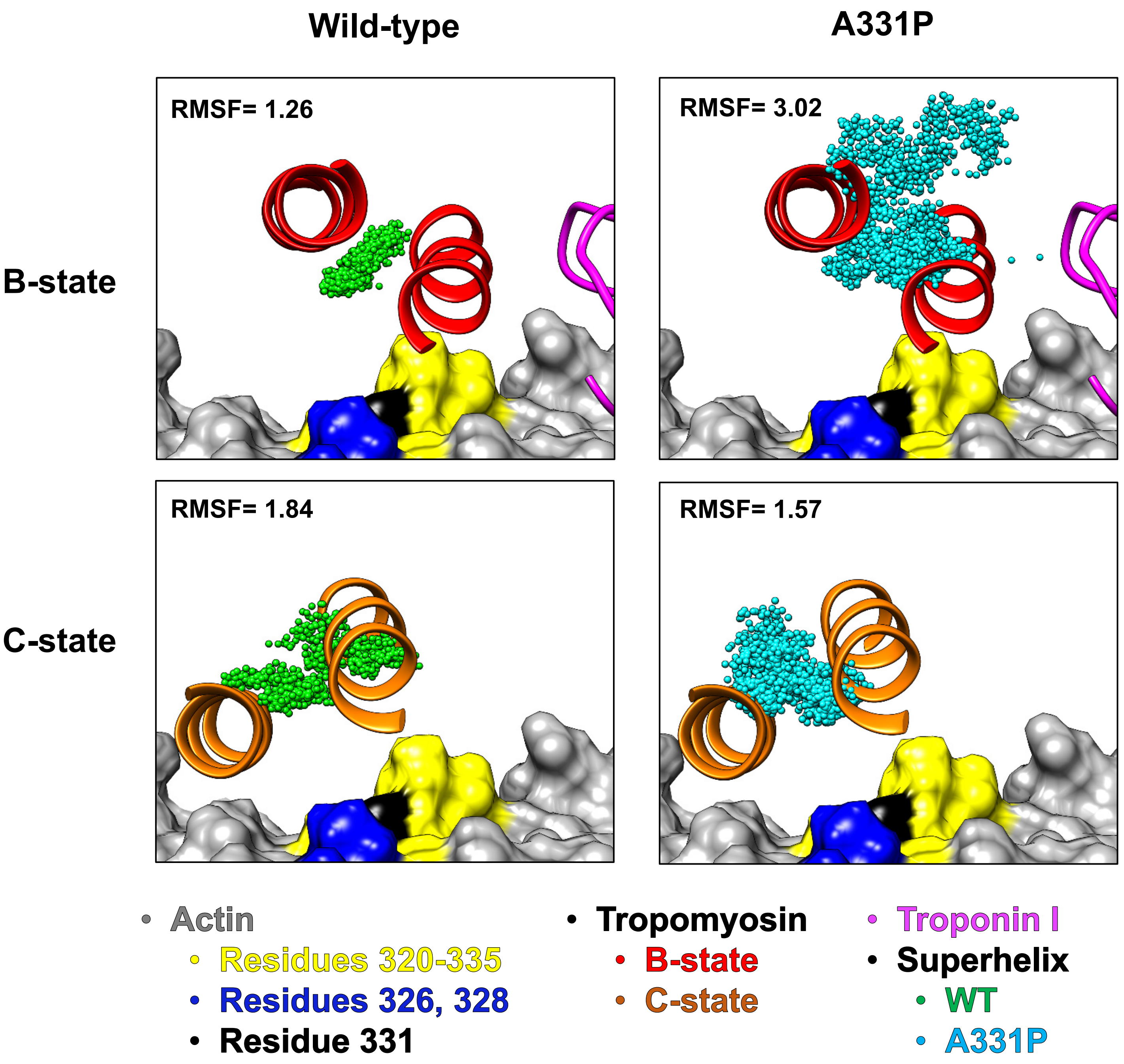

Supplementary Figure 12: PR3 Tpm superhelix positions, Tpm-Leu90. Shown is the distribution of the superhelix positions at Tpm residue 90 during the last 10 ns of MD simulation. 1000 frames for wild-type (green dots) and A331P (cyan dots) are shown, revealing the dynamic movement of the Tpm coiled coil in the vicinity of the A331P mutation site. Actin is rendered as a surface colored blue (residues Lys326 and Lys328), yellow (Pro333), black (Ala331 or Pro331), and gray (all other actin residues). Tpm in its initial position is shown as red (B-state) and orange (C-state) ribbons. TnI is shown as a magenta ribbon. Each frame examined was aligned to the initial actin coordinates underlying the Tpm residue of interest. This was done to account for any movements of the actin monomers during the simulation and to superimpose the results from the two B- or C-state Tpm dimers in the system.

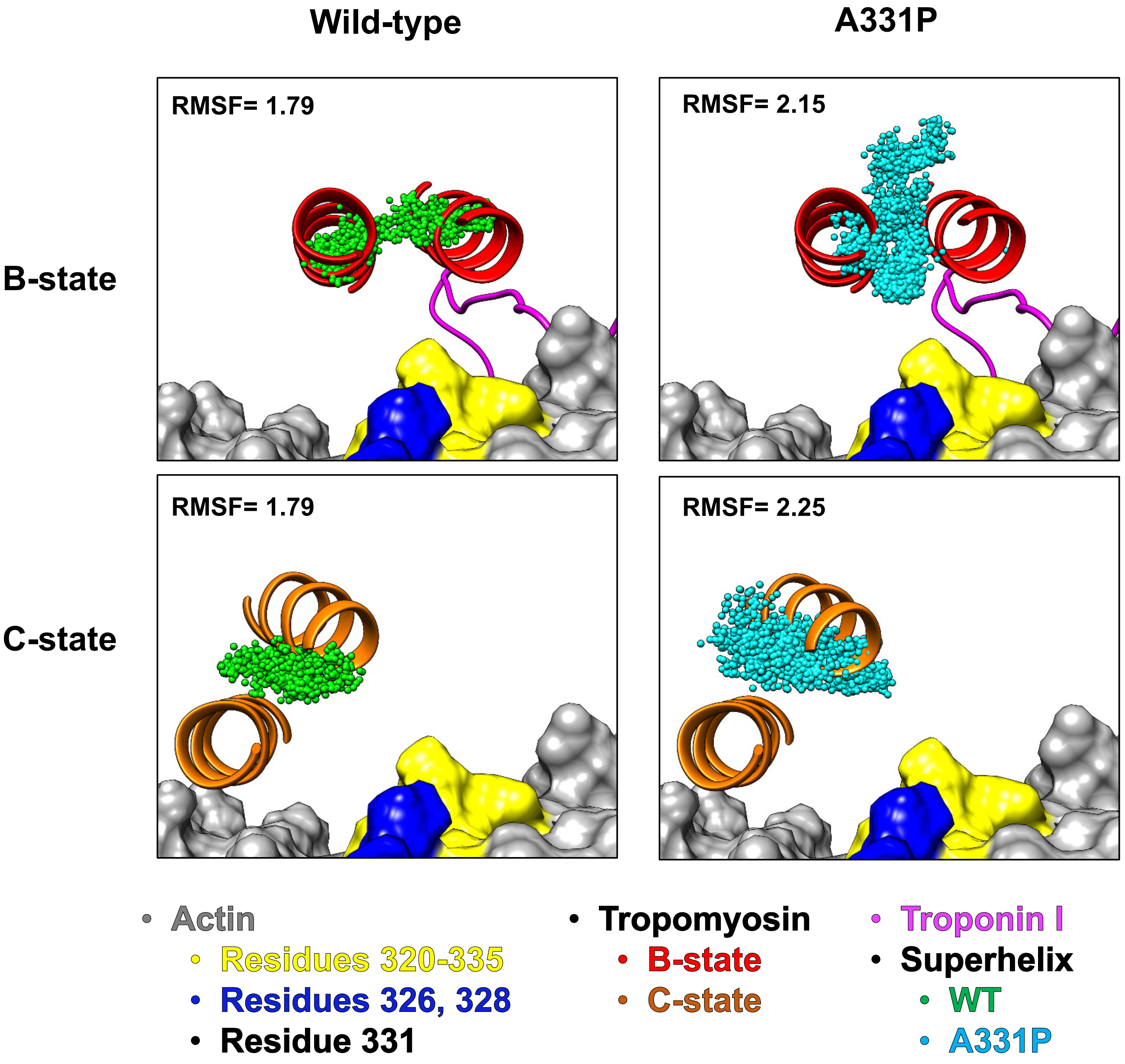

Supplementary Figure 13: PR4 Tpm superhelix positions, Tpm-Leu128. Shown is the distribution of the superhelix positions at Tpm residue 128 during the last 10 ns of MD simulation. 1000 frames for wild-type (green dots) and A331P (cyan dots) are shown, revealing the dynamic movement of the Tpm coiled coil in the vicinity of the A331P mutation site. Actin is rendered as a surface colored blue (residues Lys326 and Lys328), yellow (Pro333), black (Ala331 or Pro331), and gray (all other actin residues). Tpm in its initial position is shown as red (B-state) and orange (C-state) ribbons. TnI is shown as a magenta ribbon. Each frame examined was aligned to the initial actin coordinates underlying the Tpm residue of interest. This was done to account for any movements of the actin monomers during the simulation and to superimpose the results from the two B- or C-state Tpm dimers in the system.

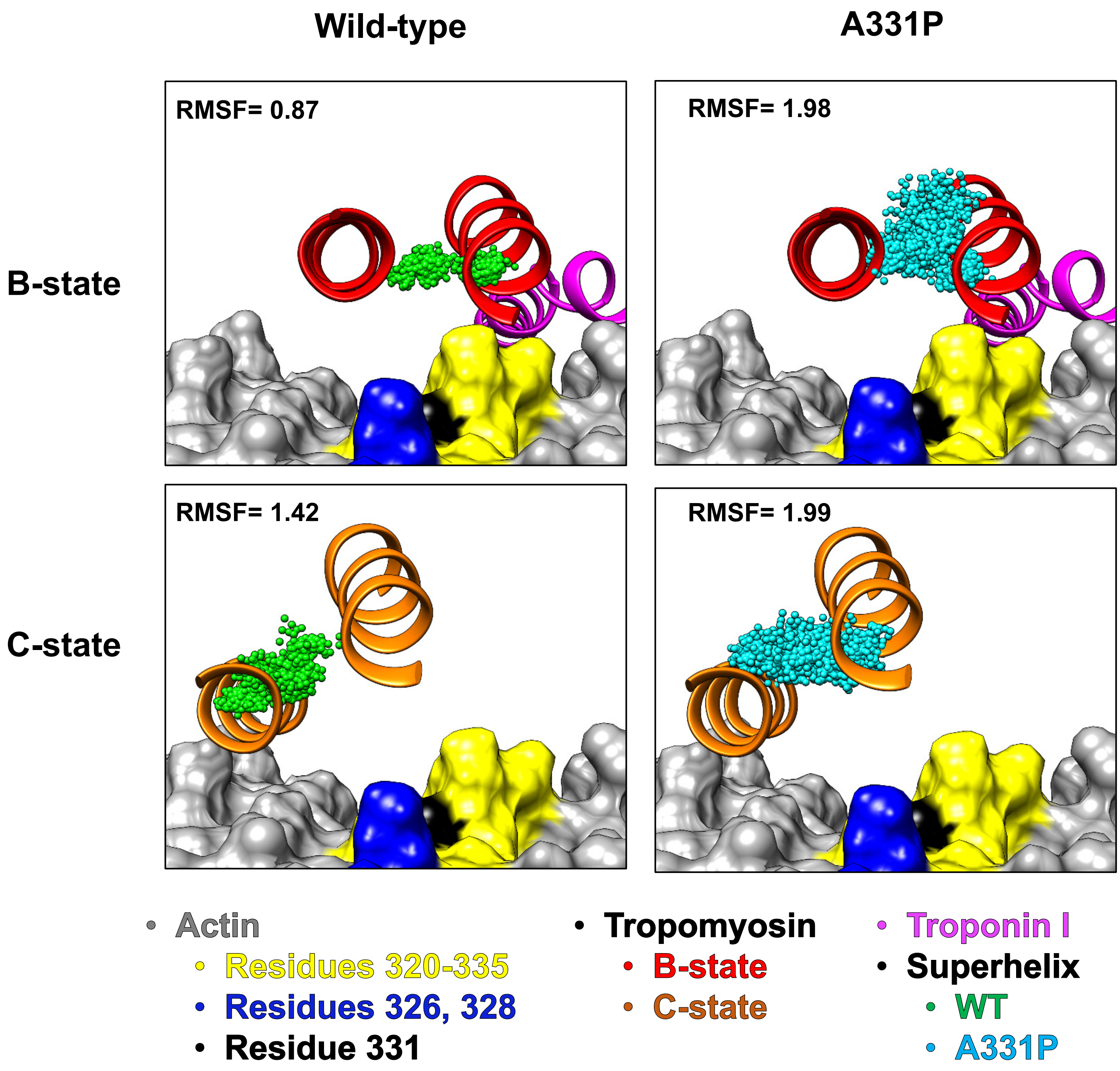

Supplementary Figure 14: PR5 Tpm superhelix positions, Tpm-Leu168. Shown is the distribution of the superhelix positions at Tpm residue 168 during the last 10 ns of MD simulation. 1000 frames for wild-type (green dots) and A331P (cyan dots) are shown, revealing the dynamic movement of the Tpm coiled coil in the vicinity of the A331P mutation site. Actin is rendered as a surface colored blue (residues Lys326 and Lys328), yellow (Pro333), black (Ala331 or Pro331), and gray (all other actin residues). Tpm in its initial position is shown as red (B-state) and orange (C-state) ribbons. TnI is shown as a magenta ribbon. Each frame examined was aligned to the initial actin coordinates underlying the Tpm residue of interest. This was done to account for any movements of the actin monomers during the simulation and to superimpose the results from the two B- or C-state Tpm dimers in the system.

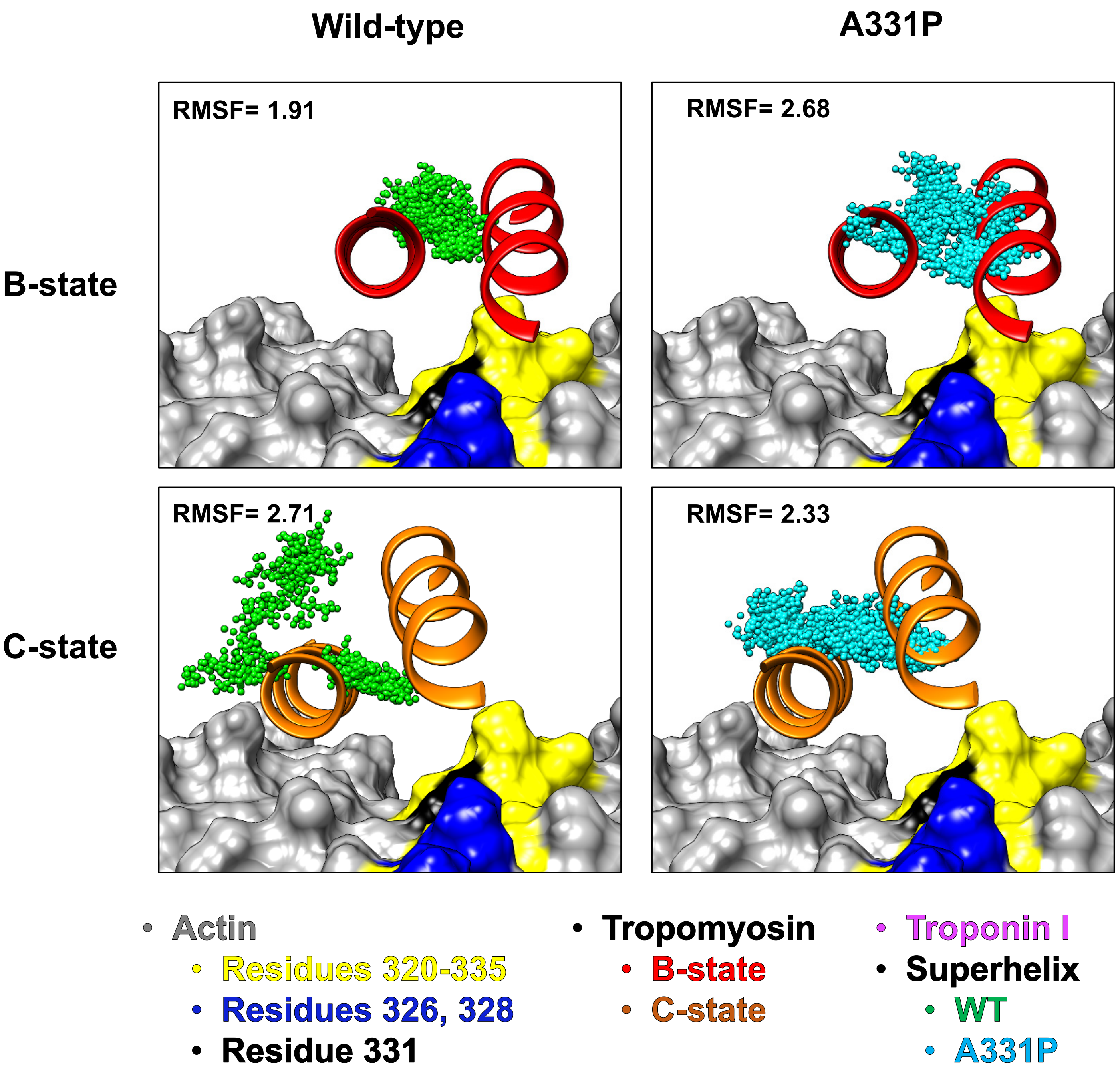

Supplementary Figure 15: PR6 Tpm superhelix positions, Tpm-Leu208. Shown is the distribution of the superhelix positions at Tpm residue 208 during the last 10 ns of MD simulation. 1000 frames for wild-type (green dots) and A331P (cyan dots) are shown, revealing the dynamic movement of the Tpm coiled coil in the vicinity of the A331P mutation site. Actin is rendered as a surface colored blue (residues Lys326 and Lys328), yellow (Pro333), black (Ala331 or Pro331), and gray (all other actin residues). Tpm in its initial position is shown as red (B-state) and orange (C-state) ribbons. TnI is shown as a magenta ribbon. Each frame examined was aligned to the initial actin coordinates underlying the Tpm residue of interest. This was done to account for any movements of the actin monomers during the simulation and to superimpose the results from the two B- or C-state Tpm dimers in the system.

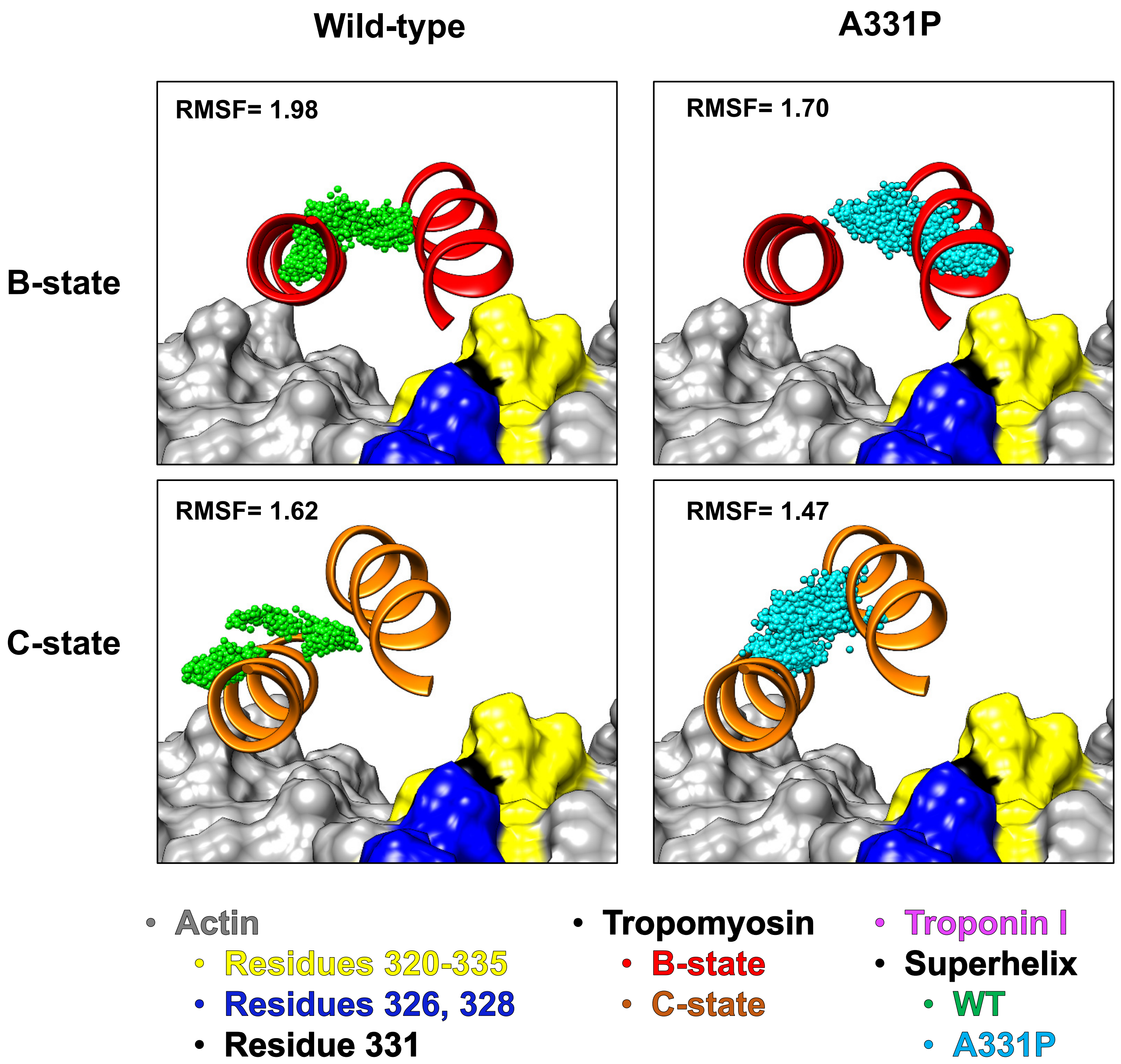

Supplementary Figure 16: PR7 Tpm superhelix positions, Tpm-Val246. Shown is the distribution of the superhelix positions at Tpm residue 246 during the last 10 ns of MD simulation. 1000 frames for wild-type (green dots) and A331P (cyan dots) are shown, revealing the dynamic movement of the Tpm coiled coil in the vicinity of the A331P mutation site. Actin is rendered as a surface colored blue (residues Lys326 and Lys328), yellow (Pro333), black (Ala331 or Pro331), and gray (all other actin residues). Tpm in its initial position is shown as red (B-state) and orange (C-state) ribbons. TnI is shown as a magenta ribbon. Each frame examined was aligned to the initial actin coordinates underlying the Tpm residue of interest. This was done to account for any movements of the actin monomers during the simulation and to superimpose the results from the two B- or C-state Tpm dimers in the system.

Cryo-EM data collection, refinement and validation statistics

	Actin WT (9B3R)	Actin A331P (9B3Q)
**Data collection and processing**		
Nominal Magnification	22500x	22500x
Voltage (kV)	300	300
Electron exposure (e–/Å^2)	60	60
Defocus range (μm)	-0.8 ~ -3	-0.8 ~ -3
Pixel size (Å)	0.529	0.529
Symmetry imposed	Helical Symmetry	Helical Symmetry
Initial segment images (no.)	1,291,946	234,263
Final segment images (no.)	133,304	140,732
Map resolution (Å)<br>FSC threshold	3.5<br>.143	3.6<br>.143
**Refinement**		
Initial model used (PDB code)	7UTL	7UTL
Model resolution (Å)<br>FSC threshold	3.6<br>0.5	3.5<br>0.5
Model resolution range (Å)	3.1-3.5	3.0-3.4
Map sharpening *B* factor (Å^2)	-138.17	-52.13
Model composition<br>Non-hydrogen atoms<br>Protein residues<br>Ligands	 8808<br>1116<br>5	 8814<br>1116<br>5
B factors (Å^2)<br>Protein<br>Ligand	 43.05<br>36.88	 59.87<br>53.86
R.m.s. deviations<br>Bond lengths (Å)<br>Bond angles (°)	 .004<br>1.015	 .006<br>1.101
Validation<br>MolProbity score<br>Clashscore<br>Poor rotamers (%)	 1.65<br>7.04<br>0.32	 1.90<br>9.49<br>1.27
Ramachandran plot<br>Favored (%)<br>Allowed (%)<br>Disallowed (%)	 96.19<br>3.81<br>0.00	 95.46<br>4.36<br>0.18

Supplementary Table 1: Statistics for data collection, data processing, model refinement, and validation.

Data S1: PDB validation reports for wild-type and mutant human cardiac F-actin, related to Figure 4.

Full wwPDB EM Validation Report (i)

May 21, 2024 – 06:14 PM EDT

PDB ID	:	9B3R
EMDB ID	:	EMD-44154
Title	:	The structure of human cardiac F-actin
Authors	:	Doran, M.H.; Sousa, D.; Rynkiewicz, M.J.; Lehman, W.; Cammarato, A.
Deposited on	:	2024-03-20
Resolution	:	3.50 Å(reported)

This is a Full wwPDB EM Validation Report for a publicly released PDB entry.

We welcome your comments at *validation@mail.wwpdb.org*
A user guide is available at
https://www.wwpdb.org/validation/2017/EMValidationReportHelp
with specific help available everywhere you see the (i) symbol.

The types of validation reports are described at
http://www.wwpdb.org/validation/2017/FAQs#types.

The following versions of software and data (see references (i)) were used in the production of this report:

EMDB validation analysis	:	0.0.1.dev92
Mogul	:	1.8.5 (274361), CSD as541be (2020)
MolProbity	:	4.02b-467
buster-report	:	1.1.7 (2018)
Percentile statistics	:	20191225.v01 (using entries in the PDB archive December 25th 2019)
MapQ	:	1.9.13
Ideal geometry (proteins)	:	Engh & Huber (2001)
Ideal geometry (DNA, RNA)	:	Parkinson et al. (1996)
Validation Pipeline (wwPDB-VP)	:	2.36.2

1 Overall quality at a glance (i)

The following experimental techniques were used to determine the structure:
ELECTRON MICROSCOPY

The reported resolution of this entry is 3.50 Å.

Percentile scores (ranging between 0-100) for global validation metrics of the entry are shown in the following graphic. The table shows the number of entries on which the scores are based.

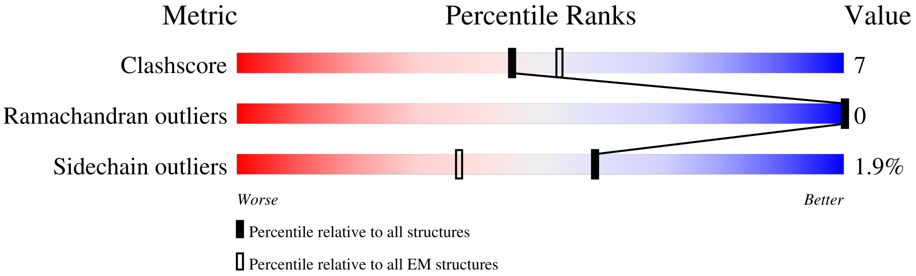

Metric	Whole archive (#Entries)	EM structures (#Entries)
Clashscore	158937	4297
Ramachandran outliers	154571	4023
Sidechain outliers	154315	3826

The table below summarises the geometric issues observed across the polymeric chains and their fit to the map. The red, orange, yellow and green segments of the bar indicate the fraction of residues that contain outliers for >=3, 2, 1 and 0 types of geometric quality criteria respectively. A grey segment represents the fraction of residues that are not modelled. The numeric value for each fraction is indicated below the corresponding segment, with a dot representing fractions <=5% The upper red bar (where present) indicates the fraction of residues that have poor fit to the EM map (all-atom inclusion < 40%). The numeric value is given above the bar.

Mol	Chain	Length	Quality of chain
1	A	377	• 81% 18% •
1	D	377	• 79% 20% •
1	E	377	• 79% 19% ••

2 Entry composition (i)

There are 3 unique types of molecules in this entry. The entry contains 8808 atoms, of which 0 are hydrogens and 0 are deuteriums.

In the tables below, the AltConf column contains the number of residues with at least one atom in alternate conformation and the Trace column contains the number of residues modelled with at most 2 atoms.

- Molecule 1 is a protein called Actin, alpha cardiac muscle 1.

Mol	Chain	Residues	Atoms					AltConf	Trace
			Total	C	N	O	S		
1	A	372	2908	1842	490	556	20	0	0
1	D	372	2908	1842	490	556	20	0	0
1	E	372	2908	1842	490	556	20	0	0

- Molecule 2 is MAGNESIUM ION (three-letter code: MG) (formula: Mg).

Mol	Chain	Residues	Atoms		AltConf
			Total	Mg	
2	A	1	1	1	0
2	D	1	1	1	0
2	E	1	1	1	0

- Molecule 3 is ADENOSINE-5'-DIPHOSPHATE (three-letter code: ADP) (formula: $C_{10}H_{15}N_5O_{10}P_2$).

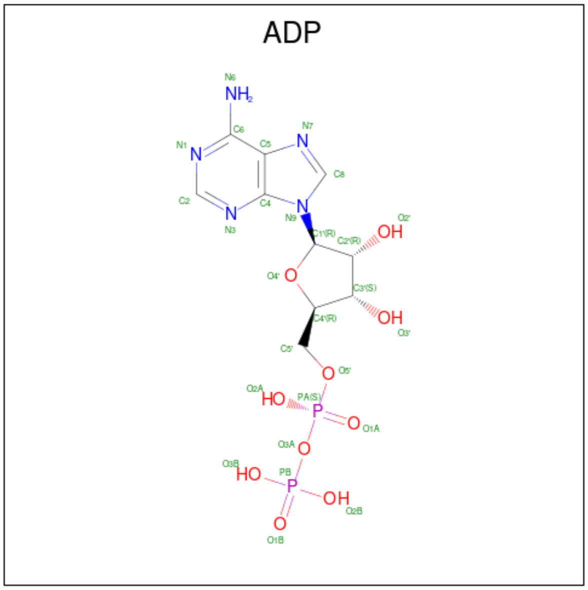

Mol	Chain	Residues	Atoms	AltConf
3	A	1	Total C N O P<br>27 10 5 10 2	0
3	D	1	Total C N O P<br>27 10 5 10 2	0
3	E	1	Total C N O P<br>27 10 5 10 2	0

3 Residue-property plots

These plots are drawn for all protein, RNA, DNA and oligosaccharide chains in the entry. The first graphic for a chain summarises the proportions of the various outlier classes displayed in the second graphic. The second graphic shows the sequence view annotated by issues in geometry and atom inclusion in map density. Residues are color-coded according to the number of geometric quality criteria for which they contain at least one outlier: green = 0, yellow = 1, orange = 2 and red = 3 or more. A red diamond above a residue indicates a poor fit to the EM map for this residue (all-atom inclusion < 40%). Stretches of 2 or more consecutive residues without any outlier are shown as a green connector. Residues present in the sample, but not in the model, are shown in grey.

- Molecule 1: Actin, alpha cardiac muscle 1

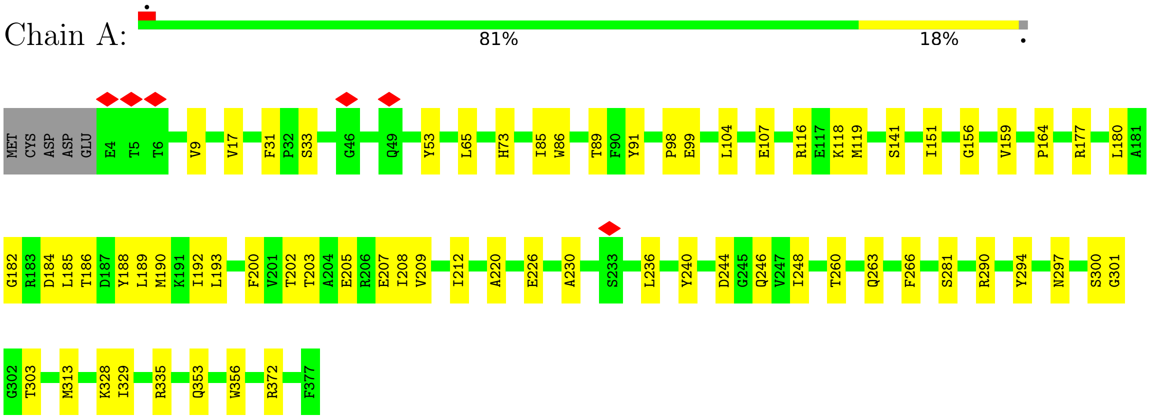

- Molecule 1: Actin, alpha cardiac muscle 1

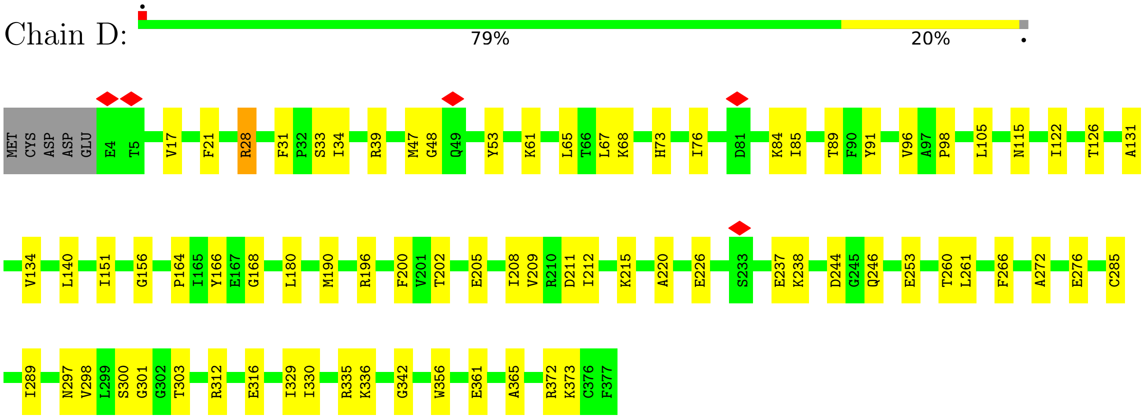

- Molecule 1: Actin, alpha cardiac muscle 1

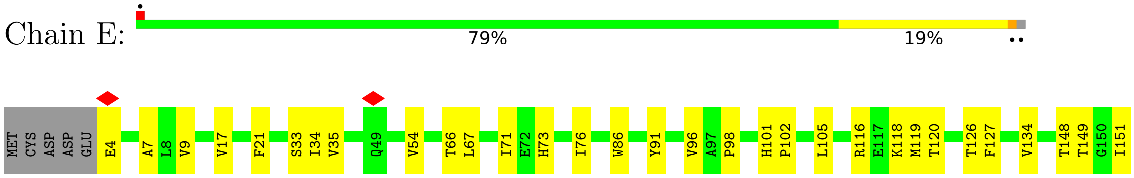

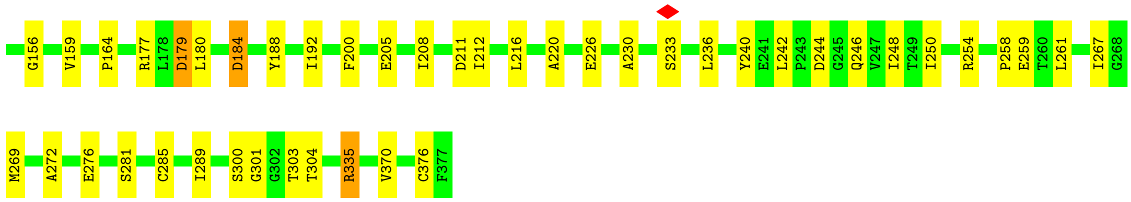
G156
V159
P164
R177
L178
D179
L180
D184
Y188
I192
F200
E205
I208
D211
I212
L216
A220
E226
A230
S233
L236
Y240
E241
L242
P243
D244
G245
Q246
V247
I248
T249
I250
R254
P258
E259
T260
L261
I267
G268
M269
A272
E276
S281
C285
I289
S300
G301
G302
T303
T304
R335
V370
C376
F377

4 Experimental information

Property	Value	Source
EM reconstruction method	HELICAL	Depositor
Imposed symmetry	HELICAL, twist=-166.48°, rise=27.93 Å, axial sym=C1	Depositor
Number of segments used	133289	Depositor
Resolution determination method	FSC 0.143 CUT-OFF	Depositor
CTF correction method	PHASE FLIPPING AND AMPLITUDE CORRECTION	Depositor
Microscope	FEI TITAN KRIOS	Depositor
Voltage (kV)	300	Depositor
Electron dose $(e^{-}/\text{Å}^{2})$	60	Depositor
Minimum defocus (nm)	600	Depositor
Maximum defocus (nm)	8000	Depositor
Magnification	Not provided	
Image detector	GATAN K3 (6k x 4k)	Depositor
Maximum map value	2.300	Depositor
Minimum map value	-1.431	Depositor
Average map value	0.000	Depositor
Map value standard deviation	0.202	Depositor
Recommended contour level	0.16	Depositor
Map size (Å)	100.509995, 106.857994, 185.15	wwPDB
Map dimensions	175, 101, 95	wwPDB
Map angles (°)	90.0, 90.0, 90.0	wwPDB
Pixel spacing (Å)	1.058, 1.058, 1.058	Depositor

5 Model quality (i)

5.1 Standard geometry (i)

Bond lengths and bond angles in the following residue types are not validated in this section: MG, HIC, ADP

The Z score for a bond length (or angle) is the number of standard deviations the observed value is removed from the expected value. A bond length (or angle) with $|Z| > 5$ is considered an outlier worth inspection. RMSZ is the root-mean-square of all Z scores of the bond lengths (or angles).

Mol	Chain	Bond lengths		Bond angles	
		RMSZ	#|Z| >5	RMSZ	#|Z| >5
1	A	0.25	0/2958	0.48	0/4005
1	D	0.24	0/2958	0.48	0/4005
1	E	0.25	0/2958	0.51	2/4005 (0.0%)
All	All	0.25	0/8874	0.49	2/12015 (0.0%)

There are no bond length outliers.

All (2) bond angle outliers are listed below:

Mol	Chain	Res	Type	Atoms	Z	Observed(^{o})	Ideal(^{o})
1	E	258	PRO	CA-N-CD	-7.39	101.16	111.50
1	E	179	ASP	CB-CG-OD2	5.02	122.82	118.30

There are no chirality outliers.

There are no planarity outliers.

5.2 Too-close contacts (i)

In the following table, the Non-H and H(model) columns list the number of non-hydrogen atoms and hydrogen atoms in the chain respectively. The H(added) column lists the number of hydrogen atoms added and optimized by MolProbity. The Clashes column lists the number of clashes within the asymmetric unit, whereas Symm-Clashes lists symmetry-related clashes.

Mol	Chain	Non-H	H(model)	H(added)	Clashes	Symm-Clashes
1	A	2908	0	2878	33	0
1	D	2908	0	2878	41	0
1	E	2908	0	2878	43	0
2	A	1	0	0	0	0
2	D	1	0	0	0	0
2	E	1	0	0	0	0

Continued on next page...

Continued from previous page...

Mol	Chain	Non-H	H(model)	H(added)	Clashes	Symm-Clashes
3	A	27	0	12	0	0
3	D	27	0	12	0	0
3	E	27	0	12	0	0
All	All	8808	0	8670	117	0

The all-atom clashscore is defined as the number of clashes found per 1000 atoms (including hydrogen atoms). The all-atom clashscore for this structure is 7.

All (117) close contacts within the same asymmetric unit are listed below, sorted by their clash magnitude.

Atom-1	Atom-2	Interatomic distance (Å)	Clash overlap (Å)
1:D:31:PHE:HZ	1:D:89:THR:HG22	1.55	0.71
1:D:361:GLU:OE2	1:D:373:LYS:NZ	2.24	0.69
1:A:53:TYR:HD2	1:A:65:LEU:HD21	1.59	0.68
1:D:17:VAL:HG23	1:D:33:SER:HB2	1.76	0.68
1:D:21:PHE:HZ	1:D:96:VAL:HG21	1.57	0.67
1:E:200:PHE:HB3	1:E:205:GLU:HB3	1.78	0.66
1:A:202:THR:OG1	1:A:205:GLU:OE1	2.14	0.65
1:A:17:VAL:HG23	1:A:33:SER:HB2	1.79	0.63
1:E:116:ARG:O	1:E:120:THR:HG23	1.99	0.62
1:D:85:ILE:O	1:D:89:THR:HG23	2.01	0.61
1:D:202:THR:OG1	1:D:205:GLU:OE1	2.18	0.61
1:E:216:LEU:HD22	1:E:250:ILE:HD11	1.83	0.61
1:E:151:ILE:HG22	1:E:164:PRO:HA	1.82	0.60
1:E:17:VAL:HG23	1:E:33:SER:HB2	1.83	0.59
1:E:148:THR:HG23	1:E:149:THR:HG23	1.85	0.59
1:E:35:VAL:HG22	1:E:54:VAL:HG12	1.84	0.58
1:D:180:LEU:HD21	1:D:261:LEU:HA	1.86	0.58
1:E:156:GLY:O	1:E:303:THR:OG1	2.21	0.58
1:A:86:TRP:HH2	1:A:119:MET:HG2	1.69	0.58
1:E:4:GLU:HG2	1:E:101:HIS:CD2	2.38	0.57
1:D:200:PHE:HB3	1:D:205:GLU:HB3	1.85	0.57
1:A:107:GLU:OE2	1:A:116:ARG:NH1	2.38	0.57
1:A:200:PHE:HB3	1:A:205:GLU:HB3	1.88	0.56
1:D:47:MET:CE	1:D:48:GLY:H	2.19	0.56
1:D:312:ARG:NH1	1:D:316:GLU:OE2	2.39	0.55
1:A:151:ILE:HG22	1:A:164:PRO:HA	1.89	0.55
1:A:240:TYR:HB3	1:A:248:ILE:HG23	1.89	0.54
1:D:260:THR:HG22	1:D:266:PHE:HB2	1.89	0.54
1:A:9:VAL:HG23	1:A:104:LEU:HB3	1.89	0.54
1:E:242:LEU:HD13	1:E:246:GLN:HB2	1.88	0.54

Continued on next page...

Continued from previous page...

Atom-1	Atom-2	Interatomic distance (Å)	Clash overlap (Å)
1:E:105:LEU:HB2	1:E:134:VAL:HG12	1.89	0.54
1:D:298:VAL:HG22	1:D:330:ILE:HD12	1.89	0.54
1:E:180:LEU:HB2	1:E:269:MET:HE1	1.90	0.53
1:A:220:ALA:HB1	1:A:226:GLU:HG3	1.91	0.53
1:E:21:PHE:HZ	1:E:96:VAL:HG21	1.74	0.53
1:D:272:ALA:HB1	1:D:276:GLU:HB3	1.89	0.53
1:A:159:VAL:HG11	1:A:177:ARG:HE	1.73	0.52
1:E:244:ASP:OD2	1:E:246:GLN:NE2	2.42	0.52
1:D:285:CYS:HB3	1:D:289:ILE:HD11	1.92	0.52
1:D:34:ILE:HD12	1:D:67:LEU:HD13	1.92	0.51
1:A:190:MET:HG3	1:A:209:VAL:HG11	1.93	0.51
1:E:180:LEU:HD21	1:E:261:LEU:HD23	1.93	0.51
1:E:159:VAL:HG11	1:E:177:ARG:HH21	1.75	0.50
1:D:211:ASP:OD1	1:D:215:LYS:NZ	2.44	0.50
1:D:53:TYR:HD2	1:D:65:LEU:HD11	1.75	0.50
1:D:91:TYR:HE1	1:D:98:PRO:HD3	1.76	0.50
1:A:244:ASP:OD2	1:A:246:GLN:NE2	2.45	0.49
1:D:220:ALA:HB1	1:D:226:GLU:HG3	1.94	0.49
1:D:156:GLY:O	1:D:303:THR:OG1	2.30	0.48
1:A:260:THR:HG22	1:A:266:PHE:HB2	1.95	0.48
1:E:9:VAL:HG23	1:E:9:VAL:O	2.14	0.48
1:E:240:TYR:HB3	1:E:248:ILE:HG23	1.95	0.48
1:A:290:ARG:O	1:A:294:TYR:HD1	1.95	0.48
1:D:151:ILE:HG22	1:D:164:PRO:HA	1.96	0.48
1:E:180:LEU:HD12	1:E:267:ILE:HD11	1.95	0.48
1:E:208:ILE:O	1:E:212:ILE:HG22	2.14	0.48
1:D:47:MET:HE3	1:D:48:GLY:H	1.77	0.47
1:D:105:LEU:HB2	1:D:134:VAL:HG12	1.96	0.47
1:E:126:THR:HG23	1:E:127:PHE:CD1	2.50	0.46
1:D:208:ILE:O	1:D:212:ILE:HG13	2.15	0.46
1:D:297:ASN:HB2	1:D:329:ILE:HA	1.97	0.46
1:A:182:GLY:O	1:A:186:THR:HG23	2.16	0.46
1:E:304:THR:O	1:E:335:ARG:NH1	2.49	0.46
1:E:259:GLU:O	1:E:259:GLU:HG3	2.14	0.46
1:E:216:LEU:HD23	1:E:216:LEU:O	2.16	0.46
1:A:31:PHE:HZ	1:A:89:THR:HG22	1.81	0.45
1:A:156:GLY:O	1:A:303:THR:OG1	2.33	0.45
1:E:285:CYS:HB3	1:E:289:ILE:HD11	1.98	0.45
1:D:196:ARG:NH1	1:D:253:GLU:OE1	2.49	0.45
1:E:7:ALA:HB2	1:E:102:PRO:HG2	1.98	0.45
1:E:188:TYR:O	1:E:192:ILE:HG23	2.16	0.45

Continued on next page...

Continued from previous page...

Atom-1	Atom-2	Interatomic distance (Å)	Clash overlap (Å)
1:E:91:TYR:HE1	1:E:98:PRO:HD3	1.81	0.45
1:A:297:ASN:HB2	1:A:329:ILE:HA	1.98	0.45
1:E:21:PHE:CZ	1:E:96:VAL:HG21	2.52	0.45
1:A:208:ILE:O	1:A:212:ILE:HG13	2.17	0.44
1:A:91:TYR:HE1	1:A:98:PRO:HD3	1.81	0.44
1:A:118:LYS:HB3	1:A:118:LYS:HE3	1.65	0.44
1:D:190:MET:HG2	1:D:209:VAL:HG21	2.00	0.44
1:E:230:ALA:HB2	1:E:236:LEU:HD12	1.99	0.44
1:A:188:TYR:O	1:A:192:ILE:HG23	2.18	0.44
1:A:353:GLN:HA	1:A:356:TRP:HE1	1.83	0.43
1:E:86:TRP:HH2	1:E:119:MET:HG2	1.83	0.43
1:A:53:TYR:CD2	1:A:65:LEU:HD21	2.46	0.43
1:E:216:LEU:HD23	1:E:254:ARG:HG2	2.00	0.43
1:E:300:SER:OG	1:E:301:GLY:N	2.52	0.43
1:A:185:LEU:HD23	1:A:185:LEU:HA	1.81	0.43
1:D:131:ALA:HB1	1:D:356:TRP:HB3	1.99	0.43
1:A:203:THR:O	1:A:207:GLU:HG2	2.18	0.43
1:E:71:ILE:HG12	1:E:76:ILE:HG13	2.00	0.43
1:E:105:LEU:HD13	1:E:119:MET:HE3	2.01	0.43
1:E:220:ALA:HB1	1:E:226:GLU:HG3	2.01	0.43
1:E:118:LYS:HB3	1:E:118:LYS:HE3	1.88	0.42
1:A:189:LEU:O	1:A:193:LEU:HB2	2.19	0.42
1:E:34:ILE:HD12	1:E:67:LEU:HD13	2.00	0.42
1:E:184:ASP:N	1:E:184:ASP:OD1	2.51	0.42
1:D:31:PHE:CZ	1:D:89:THR:HG22	2.45	0.42
1:D:84:LYS:HB3	1:D:84:LYS:HE3	1.82	0.42
1:D:237:GLU:O	1:D:238:LYS:HD3	2.20	0.42
1:E:230:ALA:O	1:E:233:SER:OG	2.30	0.42
1:A:180:LEU:HD23	1:A:180:LEU:HA	1.76	0.42
1:D:300:SER:OG	1:D:301:GLY:N	2.53	0.41
1:D:21:PHE:HD2	1:D:28:ARG:HH11	1.68	0.41
1:D:122:ILE:O	1:D:126:THR:HG22	2.19	0.41
1:E:272:ALA:HB1	1:E:276:GLU:HB3	2.02	0.41
1:D:61:LYS:HE2	1:D:61:LYS:HB3	1.83	0.41
1:E:370:VAL:O	1:E:376:CYS:HB2	2.19	0.41
1:A:230:ALA:HB2	1:A:236:LEU:HD12	2.02	0.41
1:D:140:LEU:O	1:D:342:GLY:HA3	2.21	0.41
1:E:242:LEU:H	1:E:242:LEU:HD12	1.86	0.41
1:A:85:ILE:O	1:A:89:THR:HG23	2.21	0.41
1:A:98:PRO:HD2	1:A:99:GLU:OE1	2.21	0.40
1:D:336:LYS:HB3	1:D:336:LYS:HE3	1.90	0.40

Continued on next page...

Continued from previous page...

Atom-1	Atom-2	Interatomic distance (Å)	Clash overlap (Å)
1:A:300:SER:OG	1:A:301:GLY:N	2.54	0.40
1:D:76:ILE:HD13	1:D:115:ASN:HD21	1.87	0.40
1:D:166:TYR:O	1:D:168:GLY:N	2.54	0.40
1:D:365:ALA:HB1	1:D:372:ARG:HH12	1.87	0.40
1:D:244:ASP:OD2	1:D:246:GLN:NE2	2.54	0.40

There are no symmetry-related clashes.

5.3 Torsion angles (i)

5.3.1 Protein backbone (i)

In the following table, the Percentiles column shows the percent Ramachandran outliers of the chain as a percentile score with respect to all PDB entries followed by that with respect to all EM entries.

The Analysed column shows the number of residues for which the backbone conformation was analysed, and the total number of residues.

Mol	Chain	Analysed	Favoured	Allowed	Outliers	Percentiles	
1	A	369/377 (98%)	354 (96%)	15 (4%)	0	100	100
1	D	369/377 (98%)	356 (96%)	13 (4%)	0	100	100
1	E	369/377 (98%)	355 (96%)	14 (4%)	0	100	100
All	All	1107/1131 (98%)	1065 (96%)	42 (4%)	0	100	100

There are no Ramachandran outliers to report.

5.3.2 Protein sidechains (i)

In the following table, the Percentiles column shows the percent sidechain outliers of the chain as a percentile score with respect to all PDB entries followed by that with respect to all EM entries.

The Analysed column shows the number of residues for which the sidechain conformation was analysed, and the total number of residues.

Mol	Chain	Analysed	Rotameric	Outliers	Percentiles	
1	A	314/319 (98%)	306 (98%)	8 (2%)	47	75
1	D	314/319 (98%)	310 (99%)	4 (1%)	69	86

Continued on next page...

Continued from previous page...

Mol	Chain	Analysed	Rotameric	Outliers	Percentiles	
1	E	314/319 (98%)	308 (98%)	6 (2%)	57	80
All	All	942/957 (98%)	924 (98%)	18 (2%)	59	80

All (18) residues with a non-rotameric sidechain are listed below:

Mol	Chain	Res	Type
1	A	141	SER
1	A	184	ASP
1	A	263	GLN
1	A	281	SER
1	A	313	MET
1	A	328	LYS
1	A	335	ARG
1	A	372	ARG
1	D	28	ARG
1	D	39	ARG
1	D	68	LYS
1	D	335	ARG
1	E	66	THR
1	E	179	ASP
1	E	184	ASP
1	E	211	ASP
1	E	281	SER
1	E	335	ARG

Sometimes sidechains can be flipped to improve hydrogen bonding and reduce clashes. All (2) such sidechains are listed below:

Mol	Chain	Res	Type
1	A	12	ASN
1	E	101	HIS

5.3.3 RNA ⓘ

There are no RNA molecules in this entry.

5.4 Non-standard residues in protein, DNA, RNA chains ⓘ

3 non-standard protein/DNA/RNA residues are modelled in this entry.

In the following table, the Counts columns list the number of bonds (or angles) for which Mogul

statistics could be retrieved, the number of bonds (or angles) that are observed in the model and the number of bonds (or angles) that are defined in the Chemical Component Dictionary. The Link column lists molecule types, if any, to which the group is linked. The Z score for a bond length (or angle) is the number of standard deviations the observed value is removed from the expected value. A bond length (or angle) with $|Z| > 2$ is considered an outlier worth inspection. RMSZ is the root-mean-square of all Z scores of the bond lengths (or angles).

Mol	Type	Chain	Res	Link	Bond lengths			Bond angles		
					Counts	RMSZ	$\#\|Z\|>2$	Counts	RMSZ	$\#\|Z\|>2$
1	HIC	A	73	1	8,11,12	1.64	2 (25%)	6,14,16	1.34	1 (16%)
1	HIC	E	73	1	8,11,12	1.66	2 (25%)	6,14,16	1.29	1 (16%)
1	HIC	D	73	1	8,11,12	1.65	2 (25%)	6,14,16	1.36	1 (16%)

In the following table, the Chirals column lists the number of chiral outliers, the number of chiral centers analysed, the number of these observed in the model and the number defined in the Chemical Component Dictionary. Similar counts are reported in the Torsion and Rings columns. '-' means no outliers of that kind were identified.

Mol	Type	Chain	Res	Link	Chirals	Torsions	Rings
1	HIC	A	73	1	-	2/5/6/8	0/1/1/1
1	HIC	E	73	1	-	2/5/6/8	0/1/1/1
1	HIC	D	73	1	-	2/5/6/8	0/1/1/1

All (6) bond length outliers are listed below:

Mol	Chain	Res	Type	Atoms	Z	Observed(Å)	Ideal(Å)
1	E	73	HIC	CD2-CG	3.66	1.41	1.36
1	D	73	HIC	CD2-CG	3.63	1.41	1.36
1	A	73	HIC	CD2-CG	3.60	1.41	1.36
1	D	73	HIC	CZ-NE2	-2.06	1.42	1.48
1	A	73	HIC	CZ-NE2	-2.03	1.42	1.48
1	E	73	HIC	CZ-NE2	-2.00	1.42	1.48

All (3) bond angle outliers are listed below:

Mol	Chain	Res	Type	Atoms	Z	Observed(^{o})	Ideal(^{o})
1	D	73	HIC	CB-CA-C	-2.58	106.64	111.47
1	A	73	HIC	CB-CA-C	-2.56	106.68	111.47
1	E	73	HIC	CB-CA-C	-2.42	106.93	111.47

There are no chirality outliers.

All (6) torsion outliers are listed below:

Mol	Chain	Res	Type	Atoms
1	A	73	HIC	CA-CB-CG-ND1
1	A	73	HIC	CA-CB-CG-CD2
1	D	73	HIC	CA-CB-CG-ND1
1	D	73	HIC	CA-CB-CG-CD2
1	E	73	HIC	CA-CB-CG-ND1
1	E	73	HIC	CA-CB-CG-CD2

There are no ring outliers.

No monomer is involved in short contacts.

5.5 Carbohydrates (i)

There are no monosaccharides in this entry.

5.6 Ligand geometry (i)

Of 6 ligands modelled in this entry, 3 are monoatomic - leaving 3 for Mogul analysis.

In the following table, the Counts columns list the number of bonds (or angles) for which Mogul statistics could be retrieved, the number of bonds (or angles) that are observed in the model and the number of bonds (or angles) that are defined in the Chemical Component Dictionary. The Link column lists molecule types, if any, to which the group is linked. The Z score for a bond length (or angle) is the number of standard deviations the observed value is removed from the expected value. A bond length (or angle) with $|Z| > 2$ is considered an outlier worth inspection. RMSZ is the root-mean-square of all Z scores of the bond lengths (or angles).

Mol	Type	Chain	Res	Link	Bond lengths			Bond angles		
					Counts	RMSZ	$\#\|Z\| > 2$	Counts	RMSZ	$\#\|Z\| > 2$
3	ADP	A	402	2	24,29,29	0.94	1 (4%)	29,45,45	1.51	4 (13%)
3	ADP	D	402	2	24,29,29	0.95	1 (4%)	29,45,45	1.50	4 (13%)
3	ADP	E	402	2	24,29,29	0.95	1 (4%)	29,45,45	1.50	4 (13%)

In the following table, the Chirals column lists the number of chiral outliers, the number of chiral centers analysed, the number of these observed in the model and the number defined in the Chemical Component Dictionary. Similar counts are reported in the Torsion and Rings columns. '-' means no outliers of that kind were identified.

Mol	Type	Chain	Res	Link	Chirals	Torsions	Rings
3	ADP	A	402	2	-	2/12/32/32	0/3/3/3
3	ADP	D	402	2	-	2/12/32/32	0/3/3/3
3	ADP	E	402	2	-	2/12/32/32	0/3/3/3

All (3) bond length outliers are listed below:

Mol	Chain	Res	Type	Atoms	Z	Observed(Å)	Ideal(Å)
3	D	402	ADP	C5-C4	2.46	1.47	1.40
3	A	402	ADP	C5-C4	2.46	1.47	1.40
3	E	402	ADP	C5-C4	2.44	1.47	1.40

All (12) bond angle outliers are listed below:

Mol	Chain	Res	Type	Atoms	Z	Observed(^{o})	Ideal(^{o})
3	E	402	ADP	PA-O3A-PB	-3.97	119.21	132.83
3	A	402	ADP	PA-O3A-PB	-3.91	119.42	132.83
3	D	402	ADP	PA-O3A-PB	-3.89	119.47	132.83
3	A	402	ADP	C3'-C2'-C1'	3.36	106.04	100.98
3	D	402	ADP	C3'-C2'-C1'	3.28	105.91	100.98
3	E	402	ADP	C3'-C2'-C1'	3.19	105.78	100.98
3	A	402	ADP	N3-C2-N1	-3.17	123.73	128.68
3	E	402	ADP	N3-C2-N1	-3.13	123.79	128.68
3	D	402	ADP	N3-C2-N1	-3.09	123.85	128.68
3	E	402	ADP	C4-C5-N7	-2.62	106.67	109.40
3	A	402	ADP	C4-C5-N7	-2.61	106.68	109.40
3	D	402	ADP	C4-C5-N7	-2.59	106.70	109.40

There are no chirality outliers.

All (6) torsion outliers are listed below:

Mol	Chain	Res	Type	Atoms
3	A	402	ADP	C5'-O5'-PA-O2A
3	A	402	ADP	C5'-O5'-PA-O3A
3	D	402	ADP	C5'-O5'-PA-O2A
3	D	402	ADP	C5'-O5'-PA-O3A
3	E	402	ADP	C5'-O5'-PA-O2A
3	E	402	ADP	C5'-O5'-PA-O3A

There are no ring outliers.

No monomer is involved in short contacts.

The following is a two-dimensional graphical depiction of Mogul quality analysis of bond lengths, bond angles, torsion angles, and ring geometry for all instances of the Ligand of Interest. In addition, ligands with molecular weight > 250 and outliers as shown on the validation Tables will also be included. For torsion angles, if less then 5% of the Mogul distribution of torsion angles is within 10 degrees of the torsion angle in question, then that torsion angle is considered an outlier. Any bond that is central to one or more torsion angles identified as an outlier by Mogul will be highlighted in the graph. For rings, the root-mean-square deviation (RMSD) between the ring

in question and similar rings identified by Mogul is calculated over all ring torsion angles. If the average RMSD is greater than 60 degrees and the minimal RMSD between the ring in question and any Mogul-identified rings is also greater than 60 degrees, then that ring is considered an outlier. The outliers are highlighted in purple. The color gray indicates Mogul did not find sufficient equivalents in the CSD to analyse the geometry.

Ligand ADP A 402

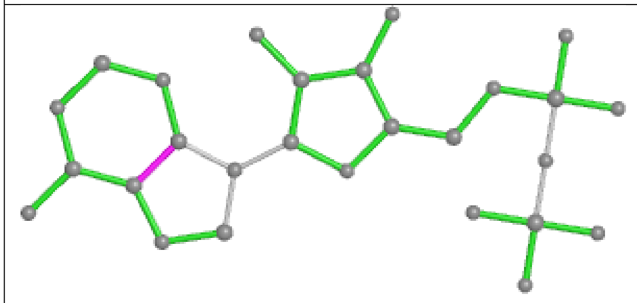

Bond lengths

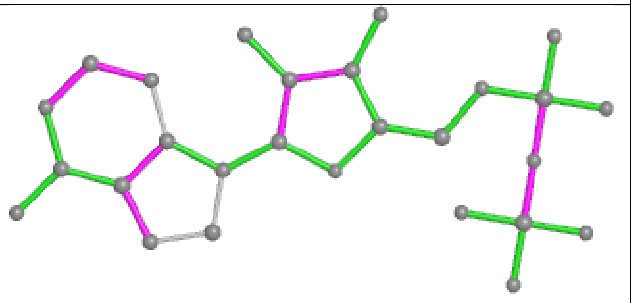

Bond angles

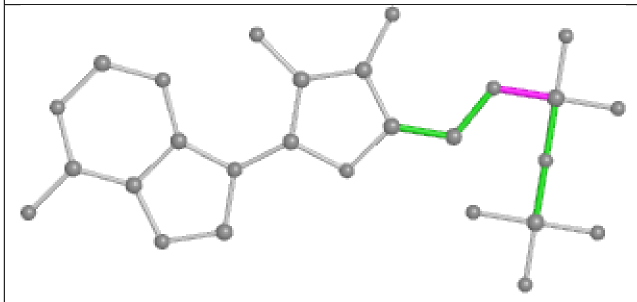

Torsions

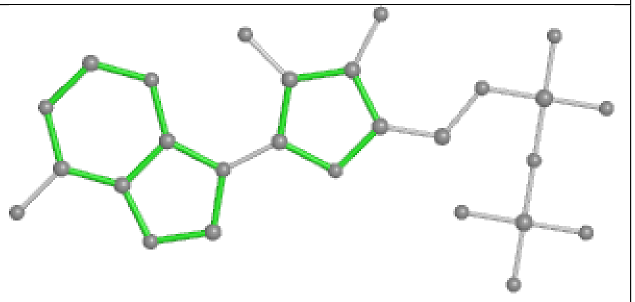

Rings

Ligand ADP D 402

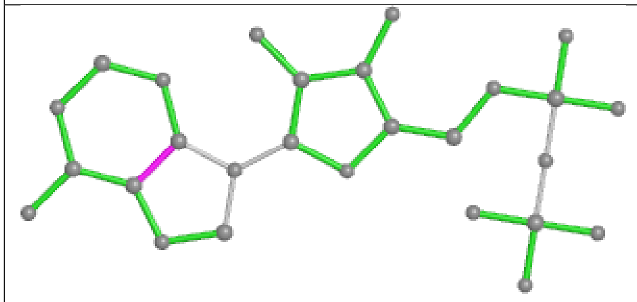

Bond lengths

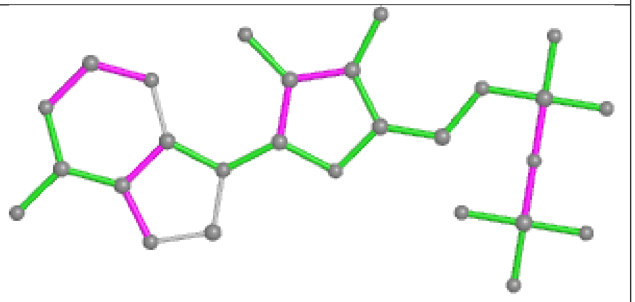

Bond angles

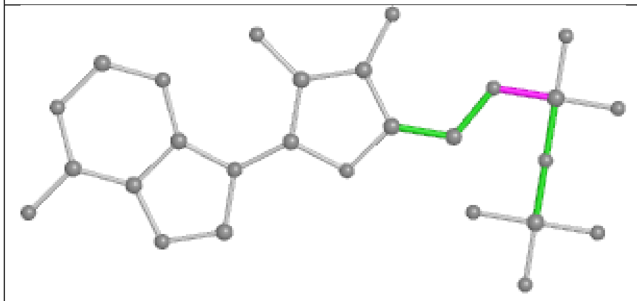

Torsions

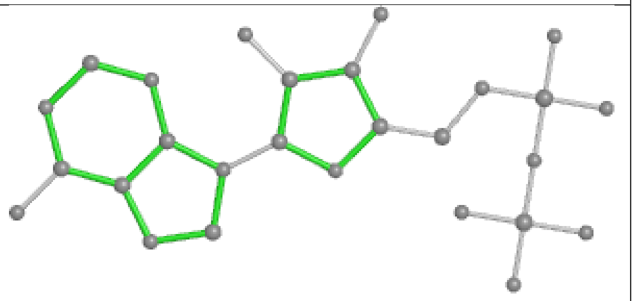

Rings

Ligand ADP E 402

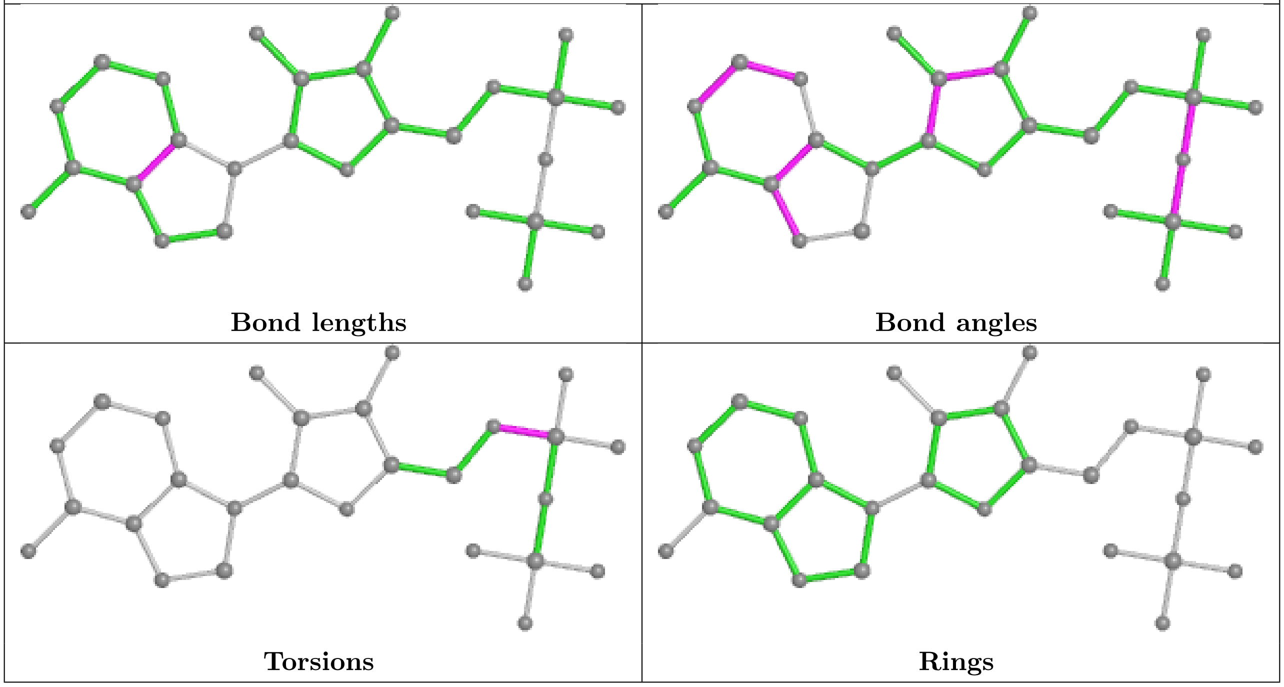

5.7 Other polymers (i)

There are no such residues in this entry.

5.8 Polymer linkage issues (i)

There are no chain breaks in this entry.

6 Map visualisation (i)

This section contains visualisations of the EMDB entry EMD-44154. These allow visual inspection of the internal detail of the map and identification of artifacts.

Images derived from a raw map, generated by summing the deposited half-maps, are presented below the corresponding image components of the primary map to allow further visual inspection and comparison with those of the primary map.

6.1 Orthogonal projections (i)

6.1.1 Primary map

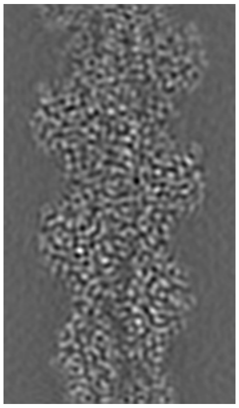
X

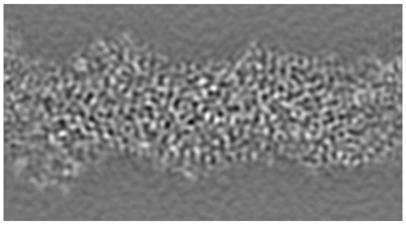
Y

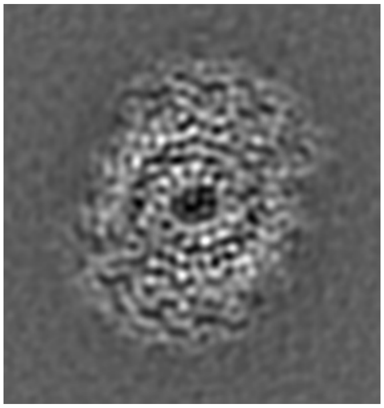
Z

6.1.2 Raw map

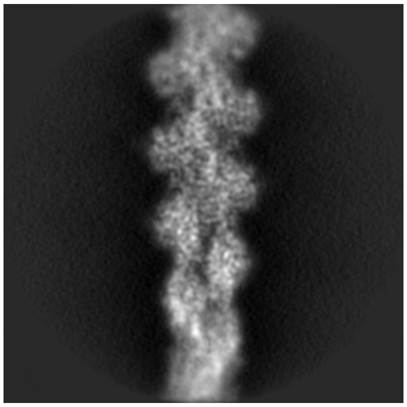
X

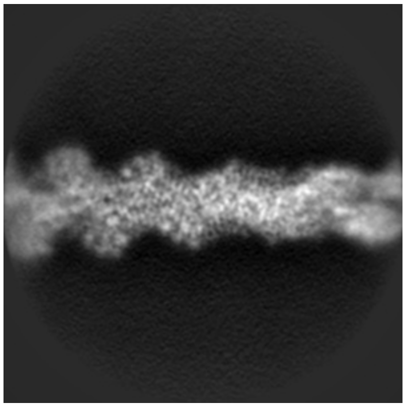
Y

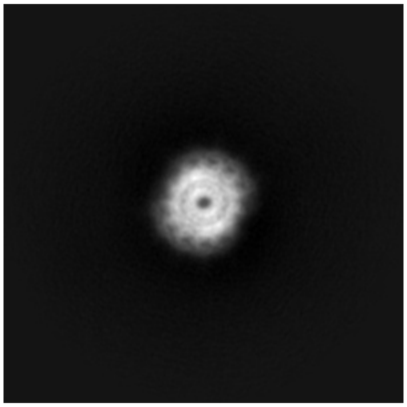
Z

The images above show the map projected in three orthogonal directions.

6.2 Central slices (i)

6.2.1 Primary map

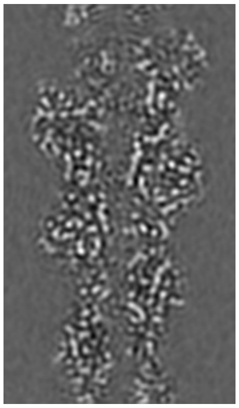

X Index: 47

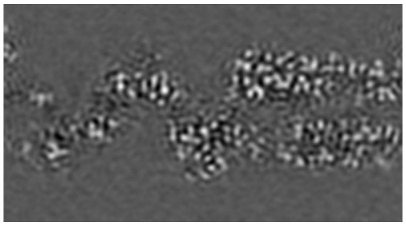

Y Index: 50

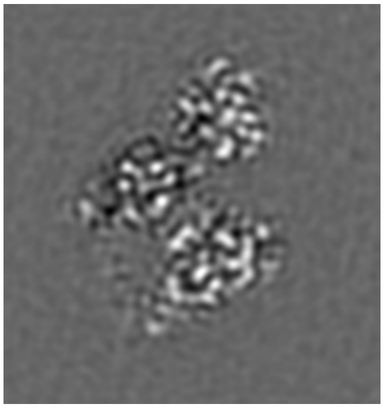

Z Index: 87

6.2.2 Raw map

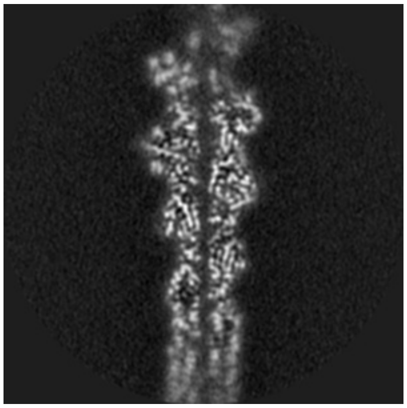

X Index: 140

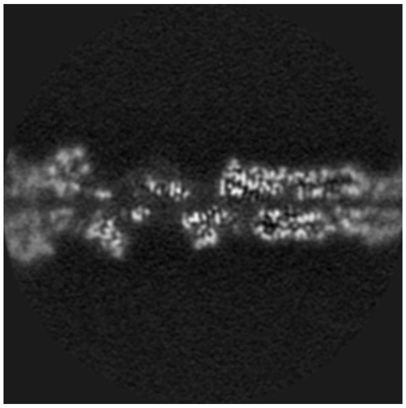

Y Index: 140

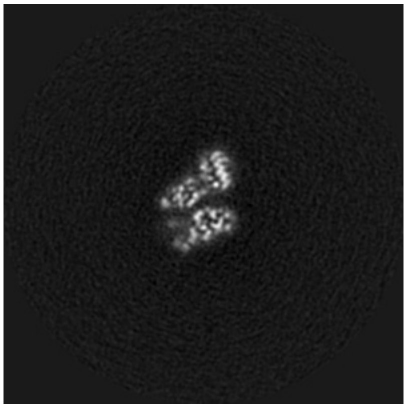

Z Index: 140

The images above show central slices of the map in three orthogonal directions.

6.3 Largest variance slices

6.3.1 Primary map

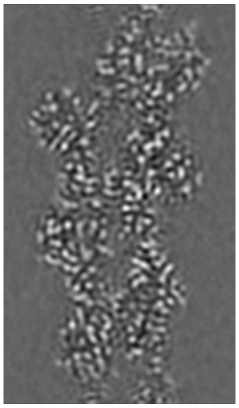

X Index: 43

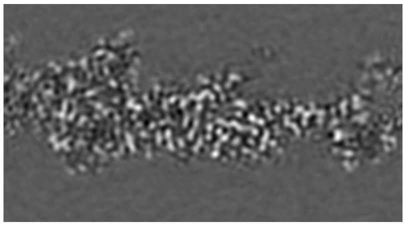

Y Index: 63

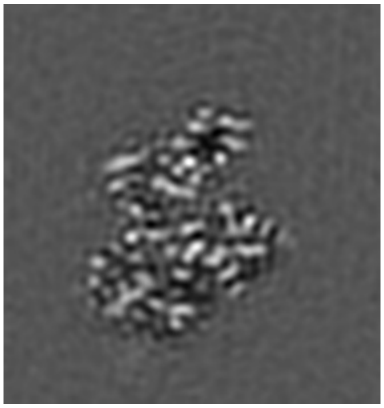

Z Index: 76

6.3.2 Raw map

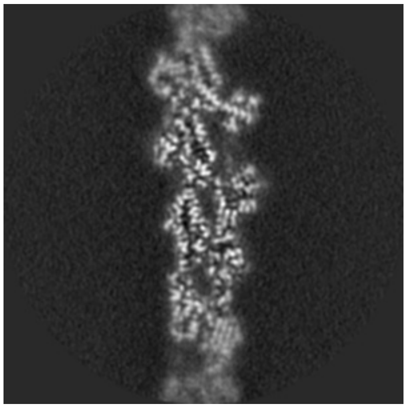

X Index: 148

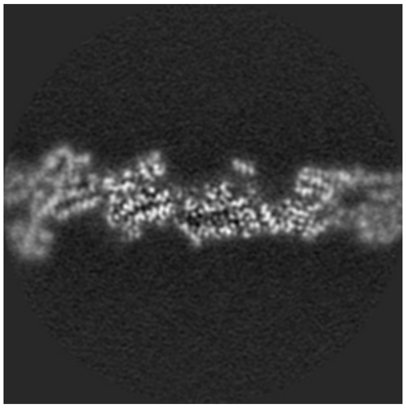

Y Index: 148

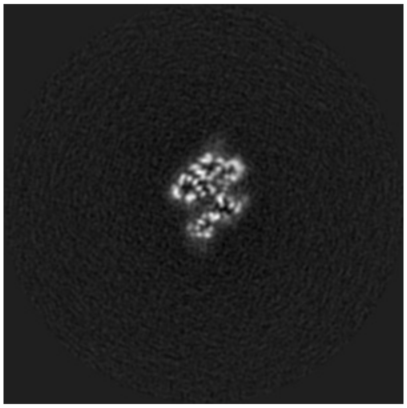

Z Index: 158

The images above show the largest variance slices of the map in three orthogonal directions.

6.4 Orthogonal standard-deviation projections (False-color) ⓘ

6.4.1 Primary map

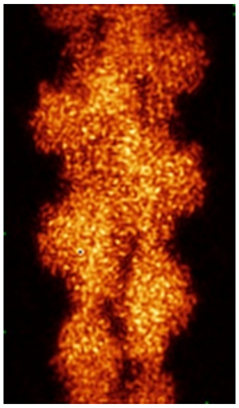
X

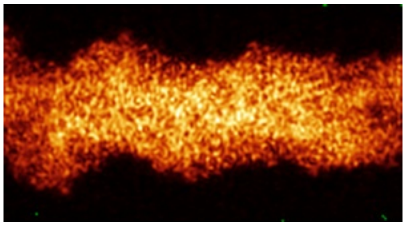
Y

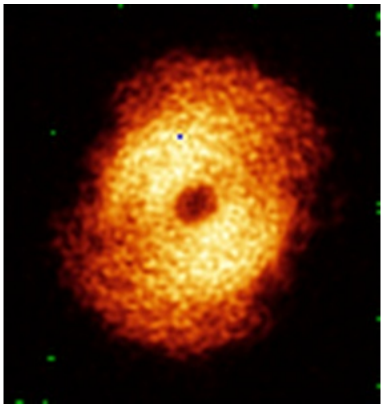
Z

6.4.2 Raw map

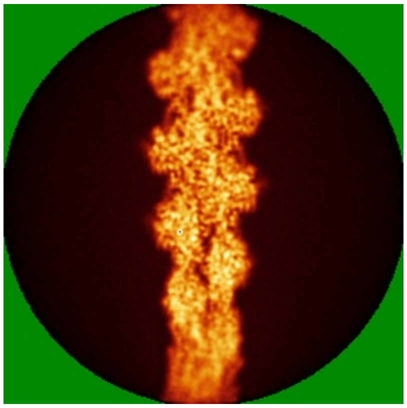
X

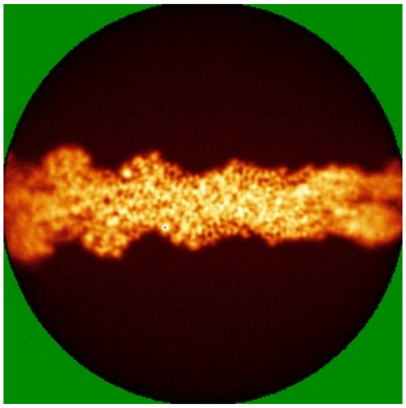
Y

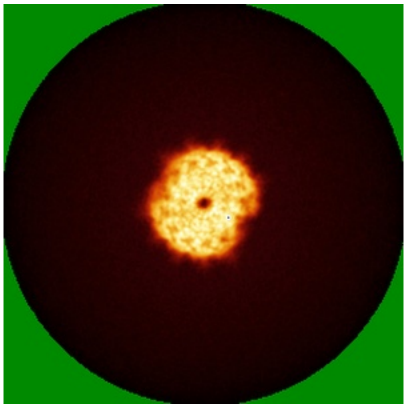
Z

The images above show the map standard deviation projections with false color in three orthogonal directions. Minimum values are shown in green, max in blue, and dark to light orange shades represent small to large values respectively.

6.5 Orthogonal surface views (i)

6.5.1 Primary map

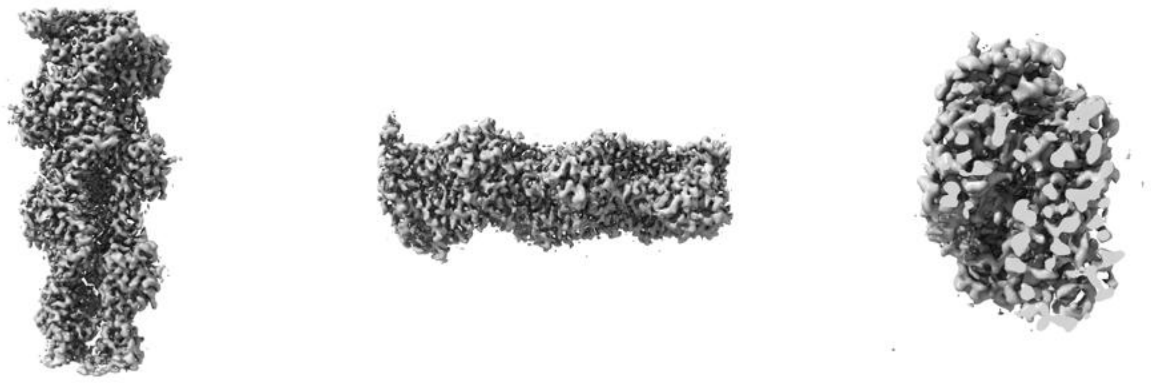

X Y Z

The images above show the 3D surface view of the map at the recommended contour level 0.16. These images, in conjunction with the slice images, may facilitate assessment of whether an appropriate contour level has been provided.

6.5.2 Raw map

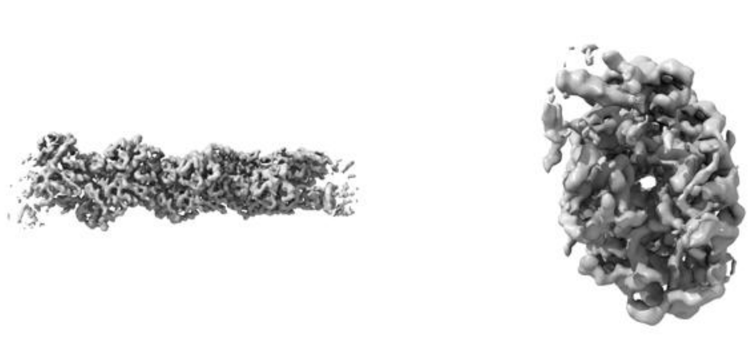

X Y Z

These images show the 3D surface of the raw map. The raw map's contour level was selected so that its surface encloses the same volume as the primary map does at its recommended contour level.

6.6 Mask visualisation (i)

This section was not generated. No masks/segmentation were deposited.

7 Map analysis (i)

This section contains the results of statistical analysis of the map.

7.1 Map-value distribution (i)

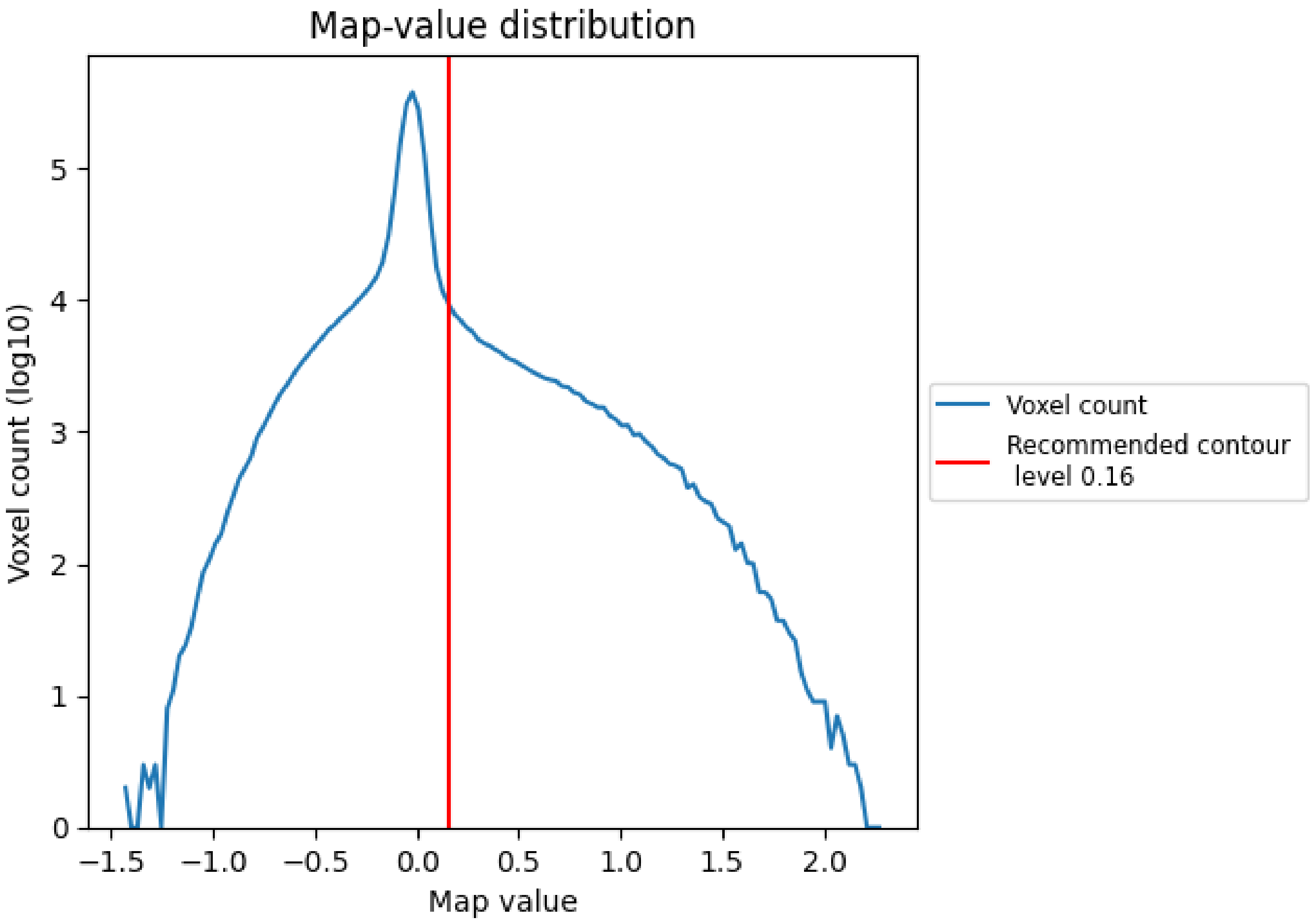

The map-value distribution is plotted in 128 intervals along the x-axis. The y-axis is logarithmic. A spike in this graph at zero usually indicates that the volume has been masked.

7.2 Volume estimate (i)

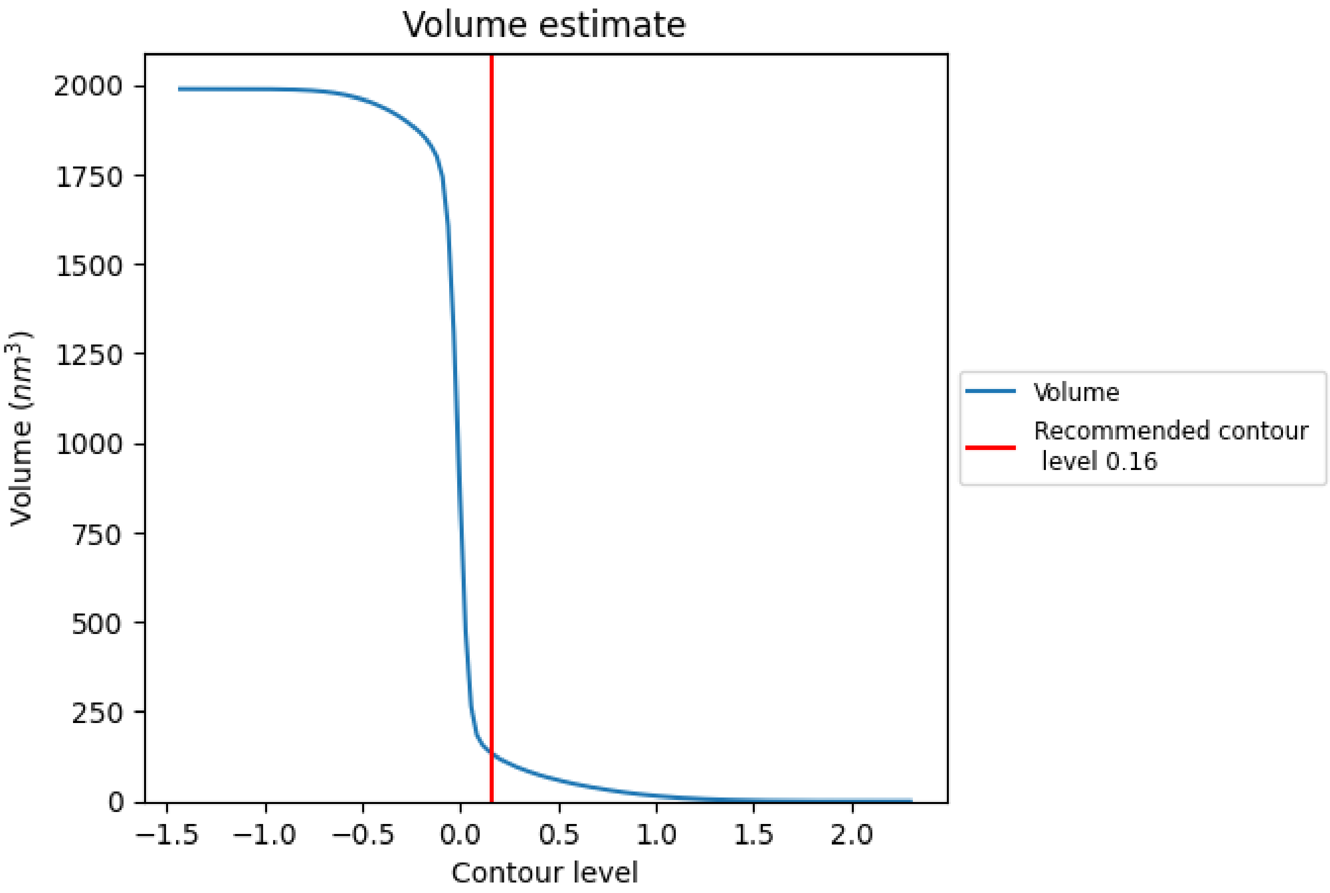

The volume at the recommended contour level is 134 nm^3; this corresponds to an approximate mass of 121 kDa.

The volume estimate graph shows how the enclosed volume varies with the contour level. The recommended contour level is shown as a vertical line and the intersection between the line and the curve gives the volume of the enclosed surface at the given level.

7.3 Rotationally averaged power spectrum (i)

This section was not generated. The rotationally averaged power spectrum is only generated for cubic maps.

8 Fourier-Shell correlation (i)

Fourier-Shell Correlation (FSC) is the most commonly used method to estimate the resolution of single-particle and subtomogram-averaged maps. The shape of the curve depends on the imposed symmetry, mask and whether or not the two 3D reconstructions used were processed from a common reference. The reported resolution is shown as a black line. A curve is displayed for the half-bit criterion in addition to lines showing the 0.143 gold standard cut-off and 0.5 cut-off.

8.1 FSC (i)

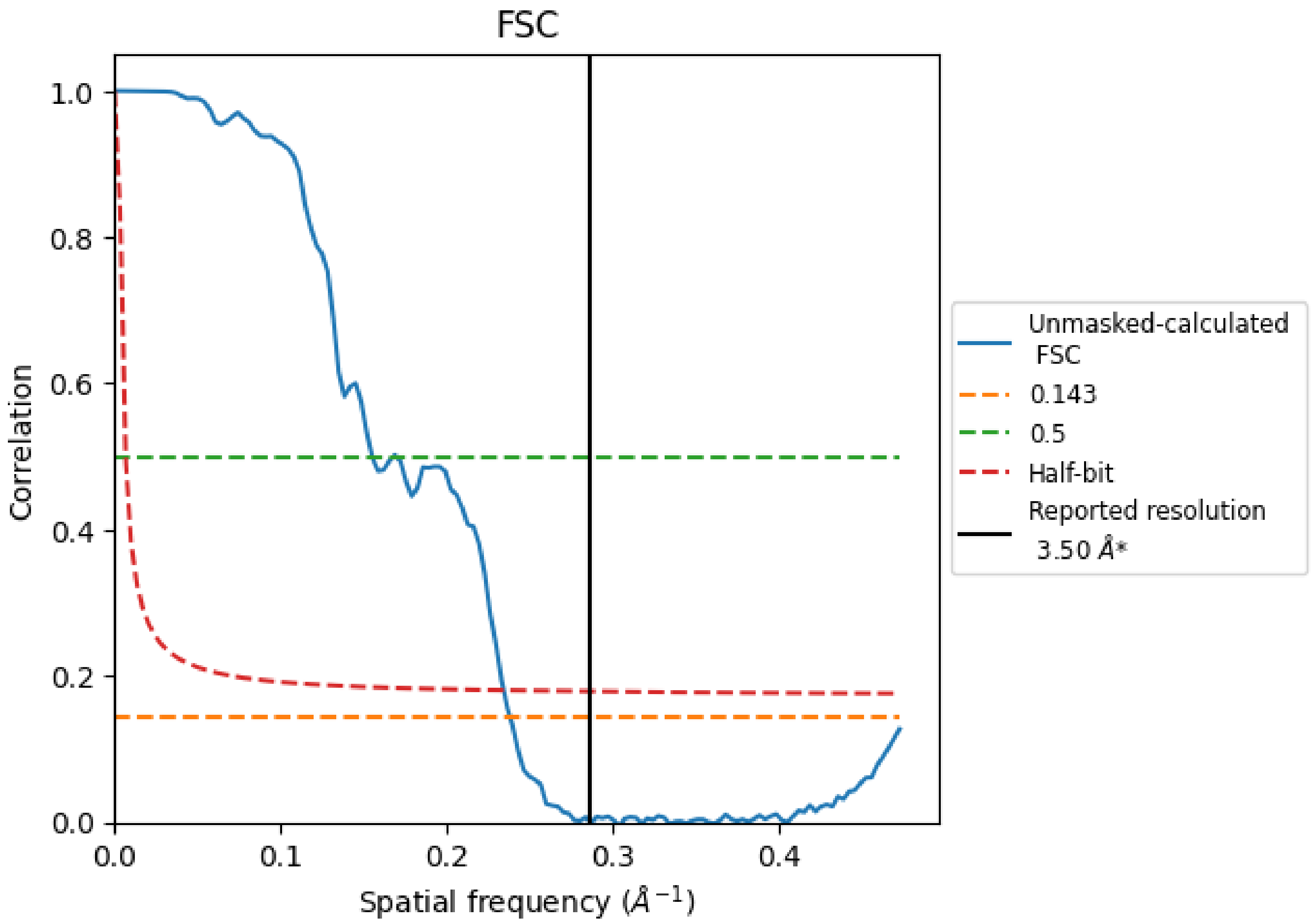

*Reported resolution corresponds to spatial frequency of 0.286 Å^{-1}

8.2 Resolution estimates (i)

Resolution estimate (Å)	Estimation criterion (FSC cut-off)		
	0.143	0.5	Half-bit
Reported by author	3.50	-	-
Author-provided FSC curve	-	-	-
Unmasked-calculated*	4.20	6.45	4.27

*Resolution estimate based on FSC curve calculated by comparison of deposited half-maps. The value from deposited half-maps intersecting FSC 0.143 CUT-OFF 4.20 differs from the reported value 3.5 by more than 10 %

9 Map-model fit (i)

This section contains information regarding the fit between EMDB map EMD-44154 and PDB model 9B3R. Per-residue inclusion information can be found in section 3 on page 5.

9.1 Map-model overlay (i)

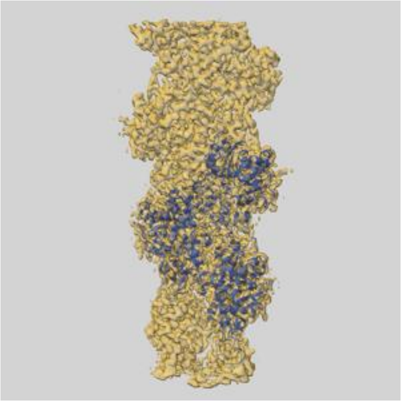

X

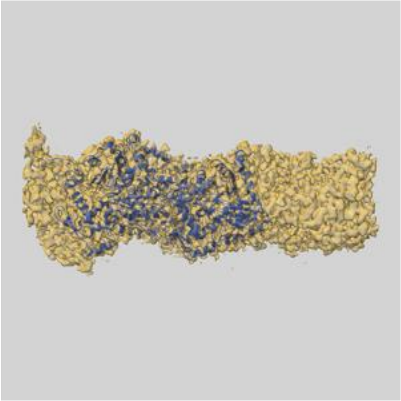

Y

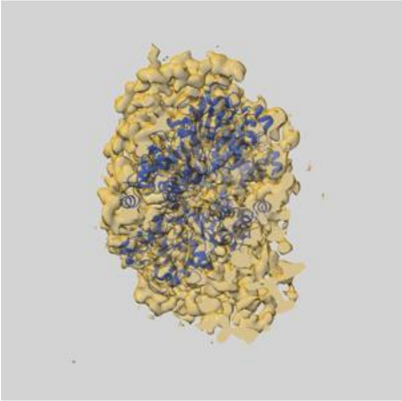

Z

The images above show the 3D surface view of the map at the recommended contour level 0.16 at 50% transparency in yellow overlaid with a ribbon representation of the model coloured in blue. These images allow for the visual assessment of the quality of fit between the atomic model and the map.

9.2 Q-score mapped to coordinate model (i)

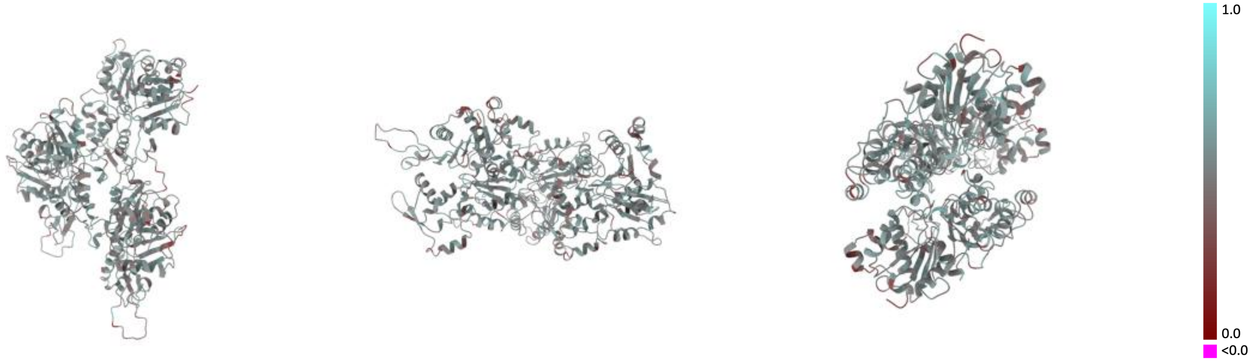

X Y Z

The images above show the model with each residue coloured according its Q-score. This shows their resolvability in the map with higher Q-score values reflecting better resolvability. Please note: Q-score is calculating the resolvability of atoms, and thus high values are only expected at resolutions at which atoms can be resolved. Low Q-score values may therefore be expected for many entries.

9.3 Atom inclusion mapped to coordinate model (i)

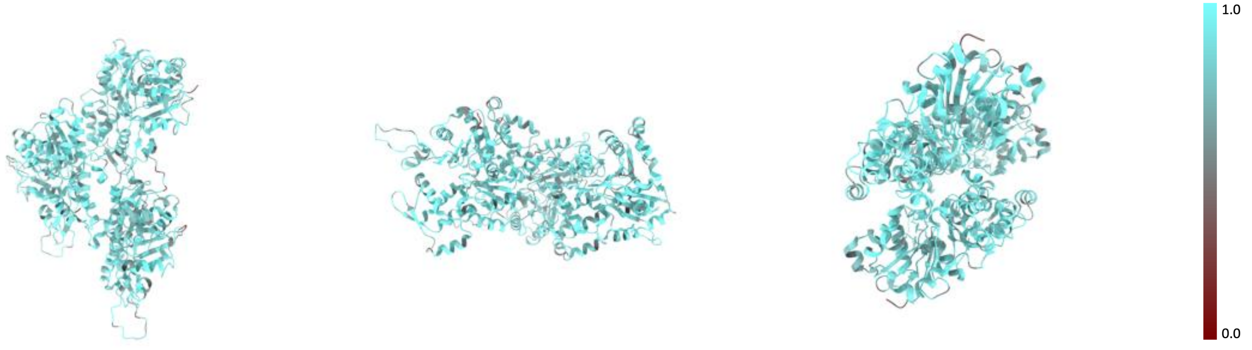

X Y Z

The images above show the model with each residue coloured according to its atom inclusion. This shows to what extent they are inside the map at the recommended contour level (0.16).

9.4 Atom inclusion (i)

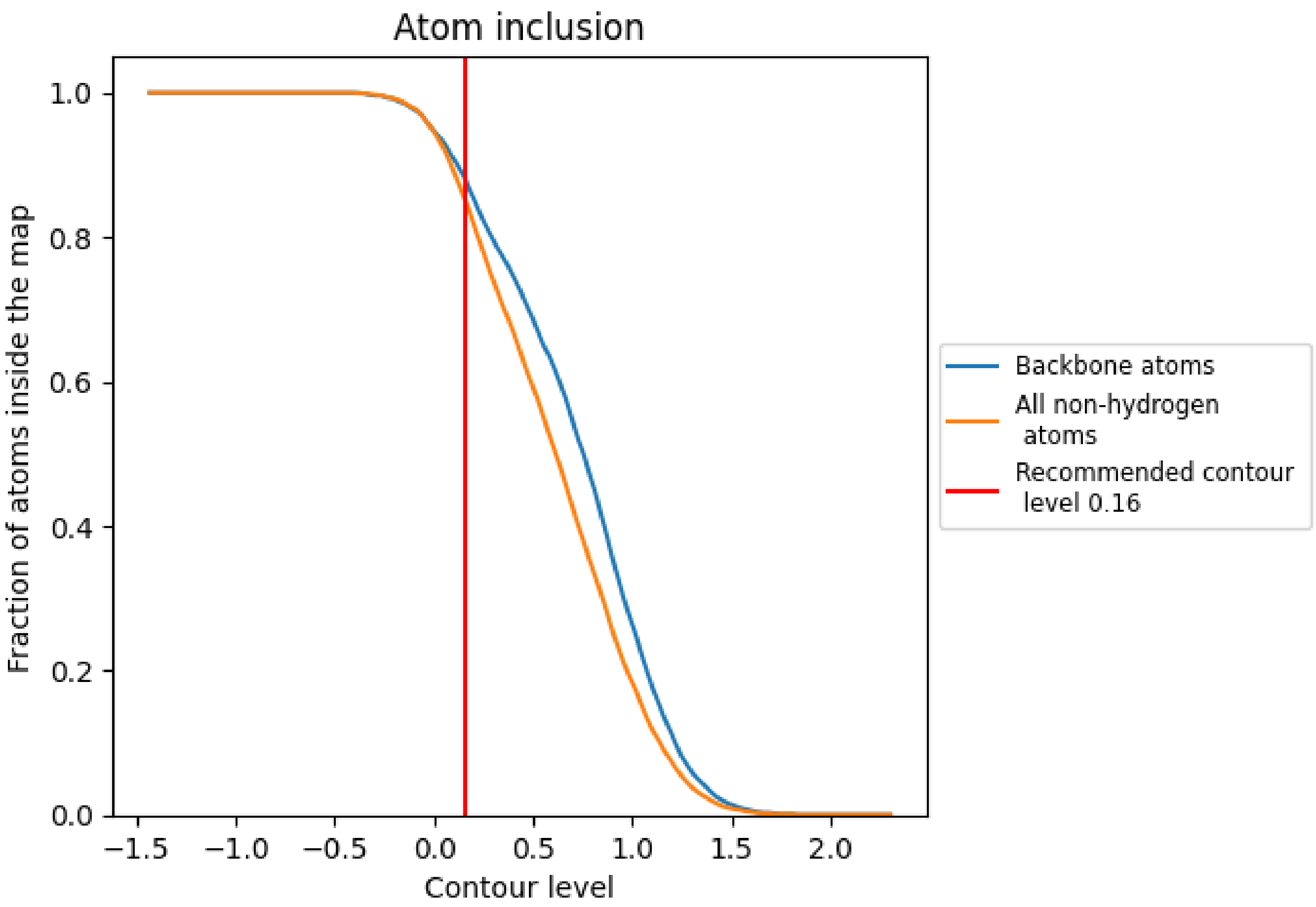

At the recommended contour level, 88% of all backbone atoms, 85% of all non-hydrogen atoms, are inside the map.

9.5 Map-model fit summary (i)

The table lists the average atom inclusion at the recommended contour level (0.16) and Q-score for the entire model and for each chain.

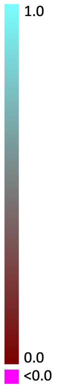

Chain	Atom inclusion	Q-score
All	0.8500	0.5010
A	0.8530	0.5050
D	0.8460	0.5000
E	0.8520	0.4990

Full wwPDB EM Validation Report (i)

May 21, 2024 – 06:16 PM EDT

PDB ID	:	9B3Q
EMDB ID	:	EMD-44153
Title	:	The structure of the human cardiac F-actin mutant A331P
Authors	:	Doran, M.H.; Sousa, D.; Rynkiewicz, M.J.; Lehman, W.; Cammarato, A.
Deposited on	:	2024-03-20
Resolution	:	3.60 Å(reported)

This is a Full wwPDB EM Validation Report for a publicly released PDB entry.

We welcome your comments at *validation@mail.wwpdb.org*
A user guide is available at
https://www.wwpdb.org/validation/2017/EMValidationReportHelp
with specific help available everywhere you see the (i) symbol.

The types of validation reports are described at
http://www.wwpdb.org/validation/2017/FAQs#types.

The following versions of software and data (see references (i)) were used in the production of this report:

EMDB validation analysis	:	0.0.1.dev92
Mogul	:	1.8.5 (274361), CSD as541be (2020)
MolProbity	:	4.02b-467
buster-report	:	1.1.7 (2018)
Percentile statistics	:	20191225.v01 (using entries in the PDB archive December 25th 2019)
MapQ	:	1.9.13
Ideal geometry (proteins)	:	Engh & Huber (2001)
Ideal geometry (DNA, RNA)	:	Parkinson et al. (1996)
Validation Pipeline (wwPDB-VP)	:	2.36.2

1 Overall quality at a glance (i)

The following experimental techniques were used to determine the structure:
ELECTRON MICROSCOPY

The reported resolution of this entry is 3.60 Å.

Percentile scores (ranging between 0-100) for global validation metrics of the entry are shown in the following graphic. The table shows the number of entries on which the scores are based.

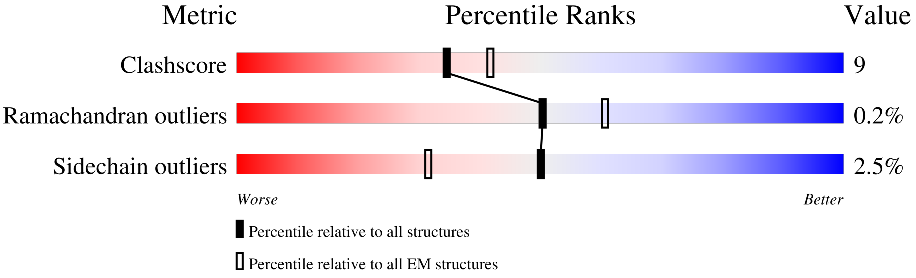

Metric	Whole archive (#Entries)	EM structures (#Entries)
Clashscore	158937	4297
Ramachandran outliers	154571	4023
Sidechain outliers	154315	3826

The table below summarises the geometric issues observed across the polymeric chains and their fit to the map. The red, orange, yellow and green segments of the bar indicate the fraction of residues that contain outliers for >=3, 2, 1 and 0 types of geometric quality criteria respectively. A grey segment represents the fraction of residues that are not modelled. The numeric value for each fraction is indicated below the corresponding segment, with a dot representing fractions <=5% The upper red bar (where present) indicates the fraction of residues that have poor fit to the EM map (all-atom inclusion < 40%). The numeric value is given above the bar.

Mol	Chain	Length	Quality of chain
1	A	377	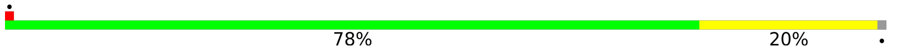
1	D	377	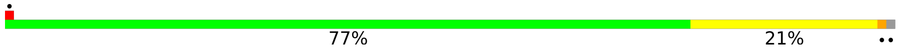
1	E	377	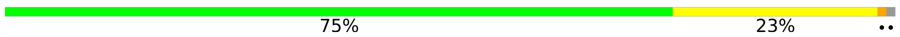

2 Entry composition (i)

There are 3 unique types of molecules in this entry. The entry contains 8814 atoms, of which 0 are hydrogens and 0 are deuteriums.

In the tables below, the AltConf column contains the number of residues with at least one atom in alternate conformation and the Trace column contains the number of residues modelled with at most 2 atoms.

- Molecule 1 is a protein called Actin, alpha cardiac muscle 1.

Mol	Chain	Residues	Atoms					AltConf	Trace
1	A	372	Total<br>2910	C<br>1844	N<br>490	O<br>556	S<br>20	0	0
1	D	372	Total<br>2910	C<br>1844	N<br>490	O<br>556	S<br>20	0	0
1	E	372	Total<br>2910	C<br>1844	N<br>490	O<br>556	S<br>20	0	0

There are 3 discrepancies between the modelled and reference sequences:

Chain	Residue	Modelled	Actual	Comment	Reference
A	331	PRO	ALA	variant	UNP P68032
D	331	PRO	ALA	variant	UNP P68032
E	331	PRO	ALA	variant	UNP P68032

- Molecule 2 is MAGNESIUM ION (three-letter code: MG) (formula: Mg).

Mol	Chain	Residues	Atoms		AltConf
2	A	1	Total<br>1	Mg<br>1	0
2	D	1	Total<br>1	Mg<br>1	0
2	E	1	Total<br>1	Mg<br>1	0

- Molecule 3 is ADENOSINE-5'-DIPHOSPHATE (three-letter code: ADP) (formula: $C_{10}H_{15}N_5O_{10}P_2$).

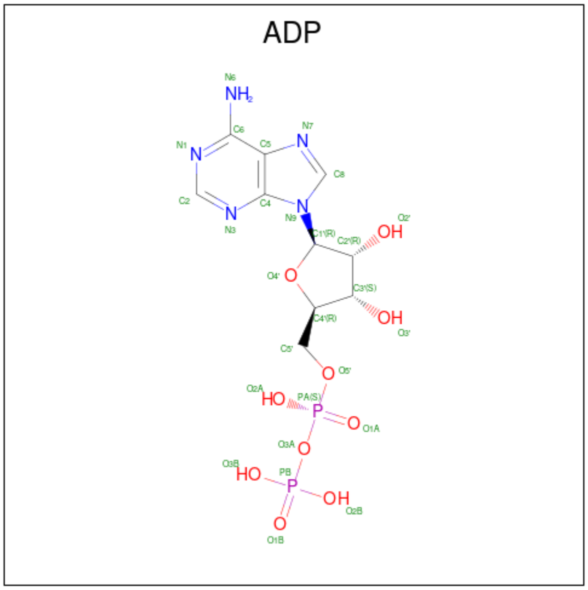

Mol	Chain	Residues	Atoms	AltConf
3	A	1	Total C N O P<br>27 10 5 10 2	0
3	D	1	Total C N O P<br>27 10 5 10 2	0
3	E	1	Total C N O P<br>27 10 5 10 2	0

3 Residue-property plots ⓘ

These plots are drawn for all protein, RNA, DNA and oligosaccharide chains in the entry. The first graphic for a chain summarises the proportions of the various outlier classes displayed in the second graphic. The second graphic shows the sequence view annotated by issues in geometry and atom inclusion in map density. Residues are color-coded according to the number of geometric quality criteria for which they contain at least one outlier: green = 0, yellow = 1, orange = 2 and red = 3 or more. A red diamond above a residue indicates a poor fit to the EM map for this residue (all-atom inclusion < 40%). Stretches of 2 or more consecutive residues without any outlier are shown as a green connector. Residues present in the sample, but not in the model, are shown in grey.

- Molecule 1: Actin, alpha cardiac muscle 1

Chain A:

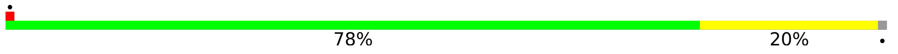

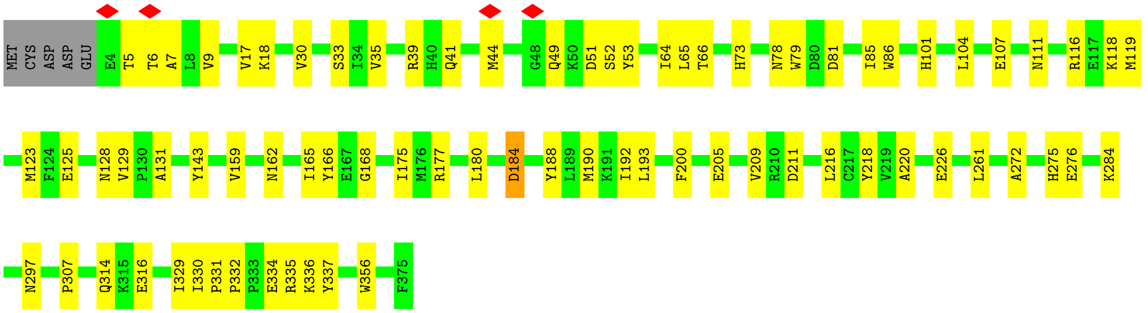

- Molecule 1: Actin, alpha cardiac muscle 1

Chain D:

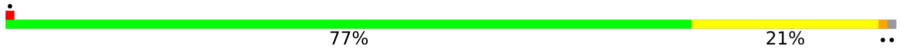

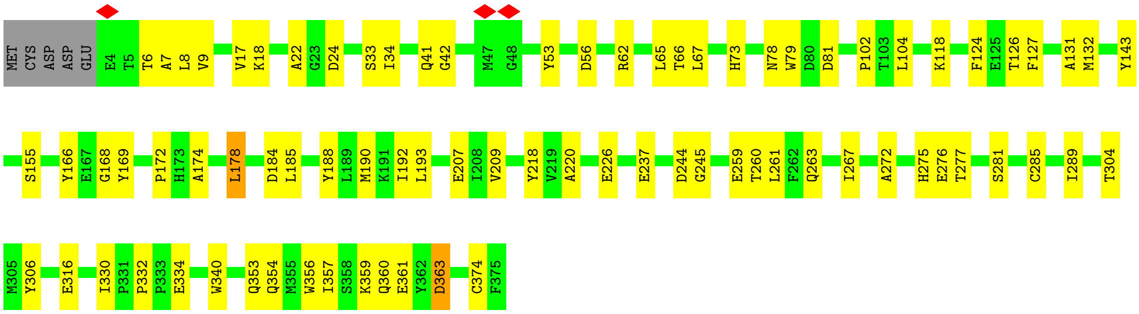

- Molecule 1: Actin, alpha cardiac muscle 1

Chain E:

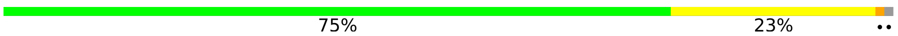

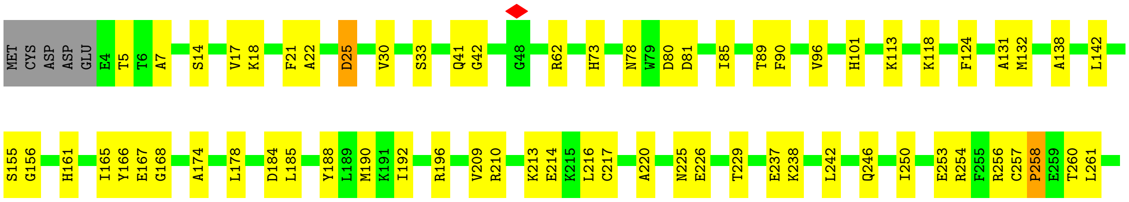

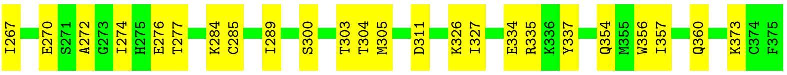
I267
E270
S271
A272
G273
I274
H275
E276
T277
K284
C285
I289
S300
T303
T304
M305
D311
K326
I327
E334
R335
K336
Y337
Q354
M355
W356
I357
Q360
K373
C374
F375

4 Experimental information (i)

Property	Value	Source
EM reconstruction method	HELICAL	Depositor
Imposed symmetry	HELICAL, twist=-166.45°, rise=27.93 Å, axial sym=C1	Depositor
Number of segments used	140667	Depositor
Resolution determination method	FSC 0.143 CUT-OFF	Depositor
CTF correction method	PHASE FLIPPING AND AMPLITUDE CORRECTION	Depositor
Microscope	FEI TITAN KRIOS	Depositor
Voltage (kV)	300	Depositor
Electron dose ($e^-/\text{Å}^2$)	60	Depositor
Minimum defocus (nm)	600	Depositor
Maximum defocus (nm)	8000	Depositor
Magnification	Not provided	
Image detector	GATAN K3 (6k x 4k)	Depositor
Maximum map value	2.144	Depositor
Minimum map value	-1.373	Depositor
Average map value	0.000	Depositor
Map value standard deviation	0.182	Depositor
Recommended contour level	0.14	Depositor
Map size (Å)	100.509995, 106.857994, 185.15	wwPDB
Map dimensions	175, 101, 95	wwPDB
Map angles (°)	90.0, 90.0, 90.0	wwPDB
Pixel spacing (Å)	1.058, 1.058, 1.058	Depositor

5 Model quality (i)

5.1 Standard geometry (i)

Bond lengths and bond angles in the following residue types are not validated in this section: HIC, MG, ADP

The Z score for a bond length (or angle) is the number of standard deviations the observed value is removed from the expected value. A bond length (or angle) with $|Z| > 5$ is considered an outlier worth inspection. RMSZ is the root-mean-square of all Z scores of the bond lengths (or angles).

Mol	Chain	Bond lengths		Bond angles	
		RMSZ	#|Z| >5	RMSZ	#|Z| >5
1	A	0.31	0/2961	0.53	0/4010
1	D	0.30	0/2961	0.51	0/4010
1	E	0.31	0/2961	0.53	1/4010 (0.0%)
All	All	0.31	0/8883	0.52	1/12030 (0.0%)

Chiral center outliers are detected by calculating the chiral volume of a chiral center and verifying if the center is modelled as a planar moiety or with the opposite hand.A planarity outlier is detected by checking planarity of atoms in a peptide group, atoms in a mainchain group or atoms of a sidechain that are expected to be planar.

Mol	Chain	#Chirality outliers	#Planarity outliers
1	E	0	1

There are no bond length outliers.

All (1) bond angle outliers are listed below:

Mol	Chain	Res	Type	Atoms	Z	Observed(^{o})	Ideal(^{o})
1	E	258	PRO	CB-CA-C	-5.31	98.72	112.00

There are no chirality outliers.

All (1) planarity outliers are listed below:

Mol	Chain	Res	Type	Group
1	E	256	ARG	Sidechain

5.2 Too-close contacts (i)

In the following table, the Non-H and H(model) columns list the number of non-hydrogen atoms and hydrogen atoms in the chain respectively. The H(added) column lists the number of hydrogen

atoms added and optimized by MolProbity. The Clashes column lists the number of clashes within the asymmetric unit, whereas Symm-Clashes lists symmetry-related clashes.

Mol	Chain	Non-H	H(model)	H(added)	Clashes	Symm-Clashes
1	A	2910	0	2880	52	0
1	D	2910	0	2880	48	0
1	E	2910	0	2880	59	0
2	A	1	0	0	0	0
2	D	1	0	0	0	0
2	E	1	0	0	0	0
3	A	27	0	12	0	0
3	D	27	0	12	2	0
3	E	27	0	12	0	0
All	All	8814	0	8676	154	0

The all-atom clashscore is defined as the number of clashes found per 1000 atoms (including hydrogen atoms). The all-atom clashscore for this structure is 9.

All (154) close contacts within the same asymmetric unit are listed below, sorted by their clash magnitude.

Atom-1	Atom-2	Interatomic distance (Å)	Clash overlap (Å)
1:E:18:LYS:NZ	1:E:337:TYR:CE1	2.45	0.84
1:E:18:LYS:HD3	1:E:337:TYR:CD1	2.19	0.77
1:E:18:LYS:HG2	1:E:30:VAL:HG12	1.69	0.75
1:A:53:TYR:HD2	1:A:65:LEU:HD21	1.55	0.72
1:D:178:LEU:HD22	1:D:277:THR:HG21	1.72	0.72
1:E:7:ALA:HB3	1:E:22:ALA:HB2	1.74	0.70
1:E:304:THR:O	1:E:335:ARG:NH1	2.26	0.69
1:E:18:LYS:HZ2	1:E:337:TYR:HE1	1.30	0.66
1:A:18:LYS:HG2	1:A:30:VAL:HG13	1.76	0.66
1:A:41:GLN:HE22	1:D:172:PRO:HD3	1.60	0.66
1:D:18:LYS:HE2	3:D:402:ADP:O2A	1.96	0.66
1:A:44:MET:SD	1:D:168:GLY:HA3	2.36	0.64
1:E:178:LEU:HD22	1:E:277:THR:HG21	1.78	0.64
1:D:190:MET:HG3	1:D:209:VAL:HG21	1.79	0.64
1:E:257:CYS:HB3	1:E:258:PRO:HD3	1.80	0.63
1:E:18:LYS:HD3	1:E:337:TYR:HD1	1.61	0.63
1:A:78:ASN:ND2	1:A:81:ASP:OD2	2.31	0.62
1:D:259:GLU:OE2	1:D:263:GLN:NE2	2.33	0.62
1:A:131:ALA:HB1	1:A:356:TRP:HB3	1.81	0.62
1:D:334:GLU:N	1:D:334:GLU:OE2	2.32	0.62
1:D:272:ALA:HB1	1:D:276:GLU:HB2	1.81	0.62
1:E:25:ASP:OD1	1:E:25:ASP:N	2.33	0.61

Continued on next page...

Continued from previous page...

Atom-1	Atom-2	Interatomic distance (Å)	Clash overlap (Å)
1:A:220:ALA:HB1	1:A:226:GLU:HG3	1.82	0.61
1:D:131:ALA:HB1	1:D:356:TRP:HB3	1.83	0.61
1:D:17:VAL:HG23	1:D:33:SER:HB2	1.83	0.60
1:A:64:ILE:HD11	1:D:166:TYR:HD2	1.67	0.59
1:A:17:VAL:HG23	1:A:33:SER:HB2	1.82	0.59
1:E:300:SER:O	1:E:304:THR:OG1	2.19	0.58
1:A:107:GLU:OE2	1:A:116:ARG:NH1	2.32	0.58
1:D:260:THR:HG21	1:D:267:ILE:HD11	1.84	0.58
1:E:131:ALA:HB1	1:E:356:TRP:HB3	1.84	0.58
1:E:216:LEU:HD23	1:E:250:ILE:HD11	1.85	0.58
1:D:53:TYR:HD2	1:D:65:LEU:HD21	1.67	0.58
1:A:18:LYS:NZ	1:A:337:TYR:CE1	2.71	0.58
1:D:220:ALA:HB1	1:D:226:GLU:HG3	1.85	0.57
1:E:78:ASN:ND2	1:E:81:ASP:OD2	2.37	0.57
1:A:190:MET:HG3	1:A:209:VAL:HG11	1.86	0.57
1:E:190:MET:HG3	1:E:209:VAL:HG11	1.86	0.57
1:E:260:THR:HG21	1:E:267:ILE:HD11	1.86	0.57
1:A:314:GLN:HB2	1:A:329:ILE:HD13	1.86	0.57
1:E:196:ARG:NH2	1:E:253:GLU:OE1	2.25	0.57
1:E:213:LYS:HA	1:E:217:CYS:SG	2.45	0.57
1:D:124:PHE:CE1	1:D:132:MET:HG2	2.40	0.56
1:D:166:TYR:O	1:D:168:GLY:N	2.35	0.56
1:D:78:ASN:ND2	1:D:81:ASP:OD2	2.39	0.55
1:A:334:GLU:O	1:A:336:LYS:N	2.38	0.55
1:A:9:VAL:HG12	1:A:104:LEU:HB3	1.88	0.55
1:D:353:GLN:NE2	1:D:354:GLN:OE1	2.38	0.55
1:E:17:VAL:HG23	1:E:33:SER:HB2	1.89	0.55
1:A:39:ARG:NH1	1:E:270:GLU:OE2	2.40	0.55
1:A:297:ASN:HB2	1:A:329:ILE:HA	1.88	0.55
1:E:166:TYR:O	1:E:168:GLY:N	2.39	0.54
1:E:167:GLU:O	1:E:167:GLU:HG3	2.08	0.54
1:D:79:TRP:NE1	1:D:118:LYS:HD3	2.23	0.54
1:E:334:GLU:OE1	1:E:334:GLU:N	2.35	0.54
1:E:220:ALA:HB1	1:E:226:GLU:HG3	1.90	0.53
1:E:261:LEU:HD12	1:E:274:ILE:HD13	1.90	0.53
1:A:51:ASP:OD1	1:A:52:SER:N	2.42	0.52
1:D:285:CYS:HB3	1:D:289:ILE:HD11	1.92	0.52
1:D:126:THR:HG22	1:D:127:PHE:CD1	2.45	0.51
1:D:174:ALA:O	1:D:281:SER:OG	2.28	0.51
1:E:156:GLY:O	1:E:303:THR:OG1	2.26	0.51
1:D:218:TYR:OH	1:D:226:GLU:OE1	2.28	0.50

Continued on next page...

Continued from previous page...

Atom-1	Atom-2	Interatomic distance (Å)	Clash overlap (Å)
1:D:9:VAL:HG12	1:D:340:TRP:NE1	2.26	0.50
1:D:237:GLU:OE2	1:D:237:GLU:N	2.38	0.50
1:E:272:ALA:HB1	1:E:276:GLU:HB2	1.91	0.50
1:E:354:GLN:HA	1:E:354:GLN:OE1	2.12	0.50
1:A:190:MET:HG2	1:A:209:VAL:HG21	1.93	0.50
1:A:81:ASP:O	1:A:85:ILE:HG22	2.12	0.50
1:D:155:SER:HB3	1:D:304:THR:HG23	1.94	0.50
1:D:34:ILE:HD12	1:D:67:LEU:HD13	1.95	0.49
1:A:41:GLN:OE1	1:A:41:GLN:N	2.37	0.49
1:A:159:VAL:HG11	1:A:177:ARG:HE	1.77	0.49
1:E:5:THR:HG21	1:E:101:HIS:HA	1.93	0.48
1:A:275:HIS:CD2	1:A:276:GLU:HG2	2.48	0.48
1:D:6:THR:HG23	1:D:7:ALA:H	1.78	0.48
1:E:285:CYS:HB3	1:E:289:ILE:HD11	1.95	0.48
1:A:18:LYS:NZ	1:A:337:TYR:HE1	2.12	0.48
1:A:18:LYS:HZ3	1:A:30:VAL:HG11	1.79	0.47
1:A:41:GLN:H	1:A:41:GLN:CD	2.18	0.47
1:A:275:HIS:CG	1:A:316:GLU:HG2	2.49	0.47
1:D:330:ILE:O	1:D:332:PRO:HD3	2.14	0.47
1:D:7:ALA:HB3	1:D:22:ALA:HB2	1.95	0.47
1:E:188:TYR:O	1:E:192:ILE:HG23	2.13	0.47
1:E:165:ILE:HG22	1:E:166:TYR:O	2.14	0.47
1:D:53:TYR:CD2	1:D:65:LEU:HD21	2.47	0.47
1:A:184:ASP:N	1:A:184:ASP:OD1	2.48	0.47
1:D:244:ASP:OD1	1:D:245:GLY:N	2.49	0.46
1:A:330:ILE:O	1:A:332:PRO:HD3	2.15	0.46
1:E:305:MET:HA	1:E:335:ARG:NH1	2.30	0.46
1:A:165:ILE:HG22	1:A:166:TYR:O	2.17	0.45
1:D:41:GLN:HG2	1:D:42:GLY:N	2.31	0.45
1:A:166:TYR:O	1:A:168:GLY:N	2.50	0.45
1:A:6:THR:HG23	1:A:7:ALA:N	2.32	0.45
1:A:86:TRP:HH2	1:A:119:MET:HG3	1.80	0.45
1:A:5:THR:HG21	1:A:101:HIS:HA	1.99	0.45
1:A:188:TYR:O	1:A:192:ILE:HG23	2.17	0.45
1:D:65:LEU:HD12	1:D:65:LEU:H	1.82	0.45
1:A:18:LYS:HZ3	1:A:30:VAL:CG1	2.30	0.45
1:A:218:TYR:HA	1:A:307:PRO:HD2	1.99	0.45
1:E:260:THR:HG21	1:E:267:ILE:CD1	2.47	0.44
1:A:297:ASN:O	1:A:330:ILE:N	2.44	0.44
1:E:18:LYS:HD3	1:E:337:TYR:CE1	2.51	0.44
1:E:41:GLN:HG2	1:E:42:GLY:N	2.33	0.44

Continued on next page...

Continued from previous page...

Atom-1	Atom-2	Interatomic distance (Å)	Clash overlap (Å)
1:A:35:VAL:HG11	1:A:81:ASP:HB3	1.99	0.44
1:A:123:MET:HG3	1:A:129:VAL:HG21	2.00	0.44
1:D:9:VAL:HG22	1:D:104:LEU:HD23	2.00	0.44
1:E:21:PHE:HZ	1:E:96:VAL:HG11	1.82	0.44
1:A:49:GLN:OE1	1:D:169:TYR:OH	2.24	0.44
1:D:357:ILE:HD11	1:D:374:CYS:SG	2.58	0.43
1:E:18:LYS:NZ	1:E:337:TYR:HE1	2.00	0.43
1:E:357:ILE:HG12	1:E:373:LYS:HD3	1.99	0.43
1:A:272:ALA:HB1	1:A:276:GLU:HB2	2.01	0.43
1:E:216:LEU:O	1:E:254:ARG:NH1	2.51	0.43
1:E:217:CYS:HB3	1:E:254:ARG:O	2.19	0.43
1:D:275:HIS:CD2	1:D:316:GLU:HG2	2.53	0.43
1:E:185:LEU:HD11	1:E:261:LEU:HD21	1.99	0.43
1:D:359:LYS:NZ	1:D:363:ASP:OD1	2.47	0.43
1:E:326:LYS:C	1:E:327:ILE:HD13	2.38	0.43
1:D:185:LEU:HD11	1:D:261:LEU:HD23	2.00	0.43
1:E:196:ARG:HE	1:E:196:ARG:HB2	1.72	0.43
1:E:210:ARG:O	1:E:214:GLU:HG3	2.19	0.43
1:D:9:VAL:HG12	1:D:340:TRP:CD1	2.54	0.43
1:E:242:LEU:HD12	1:E:246:GLN:HB2	2.00	0.43
1:E:18:LYS:CE	1:E:337:TYR:CE1	3.01	0.42
1:A:79:TRP:CZ2	1:A:118:LYS:HB3	2.54	0.42
1:D:275:HIS:CD2	1:D:276:GLU:HG2	2.55	0.42
1:A:284:LYS:HE3	1:A:284:LYS:HB3	1.82	0.42
1:D:8:LEU:N	1:D:102:PRO:O	2.53	0.42
1:A:216:LEU:HD23	1:A:216:LEU:HA	1.87	0.42
1:E:138:ALA:HB2	1:E:161:HIS:CD2	2.55	0.42
1:E:62:ARG:H	1:E:62:ARG:HG2	1.73	0.41
1:E:225:ASN:O	1:E:229:THR:HG23	2.20	0.41
1:A:162:ASN:O	1:A:175:ILE:HG23	2.20	0.41
1:A:18:LYS:HD3	1:A:337:TYR:HD1	1.86	0.41
1:D:6:THR:HG23	1:D:7:ALA:N	2.34	0.41
1:A:193:LEU:HD23	1:A:193:LEU:HA	1.88	0.41
1:A:200:PHE:HB3	1:A:205:GLU:HB3	2.01	0.41
1:D:193:LEU:HD23	1:D:193:LEU:HA	1.91	0.41
1:E:238:LYS:HB2	1:E:238:LYS:HE2	1.87	0.41
1:A:180:LEU:HD22	1:A:261:LEU:HD13	2.01	0.41
1:E:155:SER:OG	1:E:303:THR:HB	2.21	0.41
1:E:174:ALA:HA	1:E:284:LYS:HE3	2.02	0.41
1:D:188:TYR:O	1:D:192:ILE:HG23	2.21	0.41
1:E:185:LEU:HD11	1:E:261:LEU:CD2	2.51	0.41

Continued on next page...

Continued from previous page...

Atom-1	Atom-2	Interatomic distance (Å)	Clash overlap (Å)
1:D:62:ARG:NH1	1:D:207:GLU:HB2	2.35	0.41
1:E:118:LYS:HB3	1:E:118:LYS:HE3	1.88	0.41
1:A:53:TYR:CD2	1:A:65:LEU:HD21	2.45	0.40
1:A:125:GLU:HA	1:A:125:GLU:OE2	2.21	0.40
1:E:90:PHE:HD1	1:E:90:PHE:HA	1.76	0.40
1:E:237:GLU:OE1	1:E:237:GLU:N	2.41	0.40
1:E:85:ILE:O	1:E:89:THR:HG22	2.22	0.40
1:D:306:TYR:CE1	3:D:402:ADP:H2	2.39	0.40
1:E:113:LYS:HB2	1:E:113:LYS:HE2	1.87	0.40

There are no symmetry-related clashes.

5.3 Torsion angles (i)

5.3.1 Protein backbone (i)

In the following table, the Percentiles column shows the percent Ramachandran outliers of the chain as a percentile score with respect to all PDB entries followed by that with respect to all EM entries.

The Analysed column shows the number of residues for which the backbone conformation was analysed, and the total number of residues.

Mol	Chain	Analysed	Favoured	Allowed	Outliers	Percentiles	
1	A	369/377 (98%)	349 (95%)	18 (5%)	2 (0%)	29	68
1	D	369/377 (98%)	356 (96%)	13 (4%)	0	100	100
1	E	369/377 (98%)	352 (95%)	17 (5%)	0	100	100
All	All	1107/1131 (98%)	1057 (96%)	48 (4%)	2 (0%)	50	79

All (2) Ramachandran outliers are listed below:

Mol	Chain	Res	Type
1	A	331	PRO
1	A	335	ARG

5.3.2 Protein sidechains (i)

In the following table, the Percentiles column shows the percent sidechain outliers of the chain as a percentile score with respect to all PDB entries followed by that with respect to all EM

entries.

The Analysed column shows the number of residues for which the sidechain conformation was analysed, and the total number of residues.

Mol	Chain	Analysed	Rotameric	Outliers	Percentiles	
1	A	315/320 (98%)	309 (98%)	6 (2%)	57	80
1	D	315/320 (98%)	306 (97%)	9 (3%)	42	72
1	E	315/320 (98%)	306 (97%)	9 (3%)	42	72
All	All	945/960 (98%)	921 (98%)	24 (2%)	50	75

All (24) residues with a non-rotameric sidechain are listed below:

Mol	Chain	Res	Type
1	A	66	THR
1	A	111	ASN
1	A	128	ASN
1	A	143	TYR
1	A	184	ASP
1	A	211	ASP
1	D	24	ASP
1	D	56	ASP
1	D	66	THR
1	D	143	TYR
1	D	178	LEU
1	D	184	ASP
1	D	360	GLN
1	D	361	GLU
1	D	363	ASP
1	E	14	SER
1	E	25	ASP
1	E	80	ASP
1	E	124	PHE
1	E	132	MET
1	E	142	LEU
1	E	184	ASP
1	E	311	ASP
1	E	360	GLN

Sometimes sidechains can be flipped to improve hydrogen bonding and reduce clashes. All (4) such sidechains are listed below:

Mol	Chain	Res	Type
1	A	12	ASN

Continued on next page...

Continued from previous page...

Mol	Chain	Res	Type
1	A	275	HIS
1	D	263	GLN
1	D	275	HIS

5.3.3 RNA (i)

There are no RNA molecules in this entry.

5.4 Non-standard residues in protein, DNA, RNA chains (i)

3 non-standard protein/DNA/RNA residues are modelled in this entry.

In the following table, the Counts columns list the number of bonds (or angles) for which Mogul statistics could be retrieved, the number of bonds (or angles) that are observed in the model and the number of bonds (or angles) that are defined in the Chemical Component Dictionary. The Link column lists molecule types, if any, to which the group is linked. The Z score for a bond length (or angle) is the number of standard deviations the observed value is removed from the expected value. A bond length (or angle) with $|Z| > 2$ is considered an outlier worth inspection. RMSZ is the root-mean-square of all Z scores of the bond lengths (or angles).

Mol	Type	Chain	Res	Link	Bond lengths			Bond angles		
					Counts	RMSZ	#$|Z| > 2$	Counts	RMSZ	#$|Z| > 2$
1	HIC	D	73	1	8,11,12	1.66	2 (25%)	6,14,16	1.50	1 (16%)
1	HIC	A	73	1	8,11,12	1.63	2 (25%)	6,14,16	1.40	1 (16%)
1	HIC	E	73	1	8,11,12	1.64	2 (25%)	6,14,16	1.39	1 (16%)

In the following table, the Chirals column lists the number of chiral outliers, the number of chiral centers analysed, the number of these observed in the model and the number defined in the Chemical Component Dictionary. Similar counts are reported in the Torsion and Rings columns. '-' means no outliers of that kind were identified.

Mol	Type	Chain	Res	Link	Chirals	Torsions	Rings
1	HIC	D	73	1	-	2/5/6/8	0/1/1/1
1	HIC	A	73	1	-	1/5/6/8	0/1/1/1
1	HIC	E	73	1	-	1/5/6/8	0/1/1/1

All (6) bond length outliers are listed below:

Mol	Chain	Res	Type	Atoms	Z	Observed(Å)	Ideal(Å)
1	D	73	HIC	CD2-CG	3.66	1.41	1.36
1	A	73	HIC	CD2-CG	3.59	1.41	1.36

Continued on next page...

Continued from previous page...

Mol	Chain	Res	Type	Atoms	Z	Observed(Å)	Ideal(Å)
1	E	73	HIC	CD2-CG	3.58	1.41	1.36
1	D	73	HIC	CZ-NE2	-2.10	1.42	1.48
1	E	73	HIC	CZ-NE2	-2.05	1.42	1.48
1	A	73	HIC	CZ-NE2	-2.04	1.42	1.48

All (3) bond angle outliers are listed below:

Mol	Chain	Res	Type	Atoms	Z	Observed(^{o})	Ideal(^{o})
1	D	73	HIC	CB-CA-C	-2.96	105.91	111.47
1	A	73	HIC	CB-CA-C	-2.74	106.33	111.47
1	E	73	HIC	CB-CA-C	-2.64	106.52	111.47

There are no chirality outliers.

All (4) torsion outliers are listed below:

Mol	Chain	Res	Type	Atoms
1	A	73	HIC	CA-CB-CG-ND1
1	D	73	HIC	CA-CB-CG-ND1
1	E	73	HIC	CA-CB-CG-ND1
1	D	73	HIC	CA-CB-CG-CD2

There are no ring outliers.

No monomer is involved in short contacts.

5.5 Carbohydrates (i)

There are no monosaccharides in this entry.

5.6 Ligand geometry (i)

Of 6 ligands modelled in this entry, 3 are monoatomic - leaving 3 for Mogul analysis.

In the following table, the Counts columns list the number of bonds (or angles) for which Mogul statistics could be retrieved, the number of bonds (or angles) that are observed in the model and the number of bonds (or angles) that are defined in the Chemical Component Dictionary. The Link column lists molecule types, if any, to which the group is linked. The Z score for a bond length (or angle) is the number of standard deviations the observed value is removed from the expected value. A bond length (or angle) with $|Z| > 2$ is considered an outlier worth inspection. RMSZ is the root-mean-square of all Z scores of the bond lengths (or angles).

Mol	Type	Chain	Res	Link	Bond lengths			Bond angles		
					Counts	RMSZ	#|Z| > 2	Counts	RMSZ	#|Z| > 2
3	ADP	E	402	2	24,29,29	0.94	1 (4%)	29,45,45	1.59	6 (20%)
3	ADP	D	402	2	24,29,29	0.94	1 (4%)	29,45,45	1.55	5 (17%)
3	ADP	A	402	2	24,29,29	0.93	1 (4%)	29,45,45	1.62	5 (17%)

In the following table, the Chirals column lists the number of chiral outliers, the number of chiral centers analysed, the number of these observed in the model and the number defined in the Chemical Component Dictionary. Similar counts are reported in the Torsion and Rings columns. '-' means no outliers of that kind were identified.

Mol	Type	Chain	Res	Link	Chirals	Torsions	Rings
3	ADP	E	402	2	-	2/12/32/32	0/3/3/3
3	ADP	D	402	2	-	2/12/32/32	0/3/3/3
3	ADP	A	402	2	-	2/12/32/32	0/3/3/3

All (3) bond length outliers are listed below:

Mol	Chain	Res	Type	Atoms	Z	Observed(Å)	Ideal(Å)
3	D	402	ADP	C5-C4	2.34	1.47	1.40
3	A	402	ADP	C5-C4	2.30	1.47	1.40
3	E	402	ADP	C5-C4	2.29	1.47	1.40

All (16) bond angle outliers are listed below:

Mol	Chain	Res	Type	Atoms	Z	Observed(^{o})	Ideal(^{o})
3	A	402	ADP	PA-O3A-PB	-4.91	115.99	132.83
3	E	402	ADP	PA-O3A-PB	-4.74	116.56	132.83
3	D	402	ADP	PA-O3A-PB	-4.36	117.86	132.83
3	A	402	ADP	N3-C2-N1	-3.22	123.65	128.68
3	A	402	ADP	C3'-C2'-C1'	3.12	105.67	100.98
3	E	402	ADP	N3-C2-N1	-2.98	124.03	128.68
3	D	402	ADP	C3'-C2'-C1'	2.96	105.43	100.98
3	D	402	ADP	N3-C2-N1	-2.91	124.13	128.68
3	E	402	ADP	C3'-C2'-C1'	2.90	105.35	100.98
3	E	402	ADP	C4-C5-N7	-2.62	106.67	109.40
3	D	402	ADP	C4-C5-N7	-2.55	106.74	109.40
3	A	402	ADP	C4-C5-N7	-2.44	106.85	109.40
3	E	402	ADP	C2'-C3'-C4'	2.07	106.66	102.64
3	E	402	ADP	O3B-PB-O2B	2.04	115.43	107.64
3	A	402	ADP	C2'-C3'-C4'	2.03	106.59	102.64
3	D	402	ADP	C2'-C3'-C4'	2.01	106.55	102.64

There are no chirality outliers.

All (6) torsion outliers are listed below:

Mol	Chain	Res	Type	Atoms
3	A	402	ADP	C5'-O5'-PA-O3A
3	D	402	ADP	C5'-O5'-PA-O2A
3	D	402	ADP	C5'-O5'-PA-O3A
3	E	402	ADP	C5'-O5'-PA-O2A
3	E	402	ADP	C5'-O5'-PA-O3A
3	A	402	ADP	C5'-O5'-PA-O2A

There are no ring outliers.

1 monomer is involved in 2 short contacts:

Mol	Chain	Res	Type	Clashes	Symm-Clashes
3	D	402	ADP	2	0

The following is a two-dimensional graphical depiction of Mogul quality analysis of bond lengths, bond angles, torsion angles, and ring geometry for all instances of the Ligand of Interest. In addition, ligands with molecular weight > 250 and outliers as shown on the validation Tables will also be included. For torsion angles, if less then 5% of the Mogul distribution of torsion angles is within 10 degrees of the torsion angle in question, then that torsion angle is considered an outlier. Any bond that is central to one or more torsion angles identified as an outlier by Mogul will be highlighted in the graph. For rings, the root-mean-square deviation (RMSD) between the ring in question and similar rings identified by Mogul is calculated over all ring torsion angles. If the average RMSD is greater than 60 degrees and the minimal RMSD between the ring in question and any Mogul-identified rings is also greater than 60 degrees, then that ring is considered an outlier. The outliers are highlighted in purple. The color gray indicates Mogul did not find sufficient equivalents in the CSD to analyse the geometry.

Ligand ADP E 402

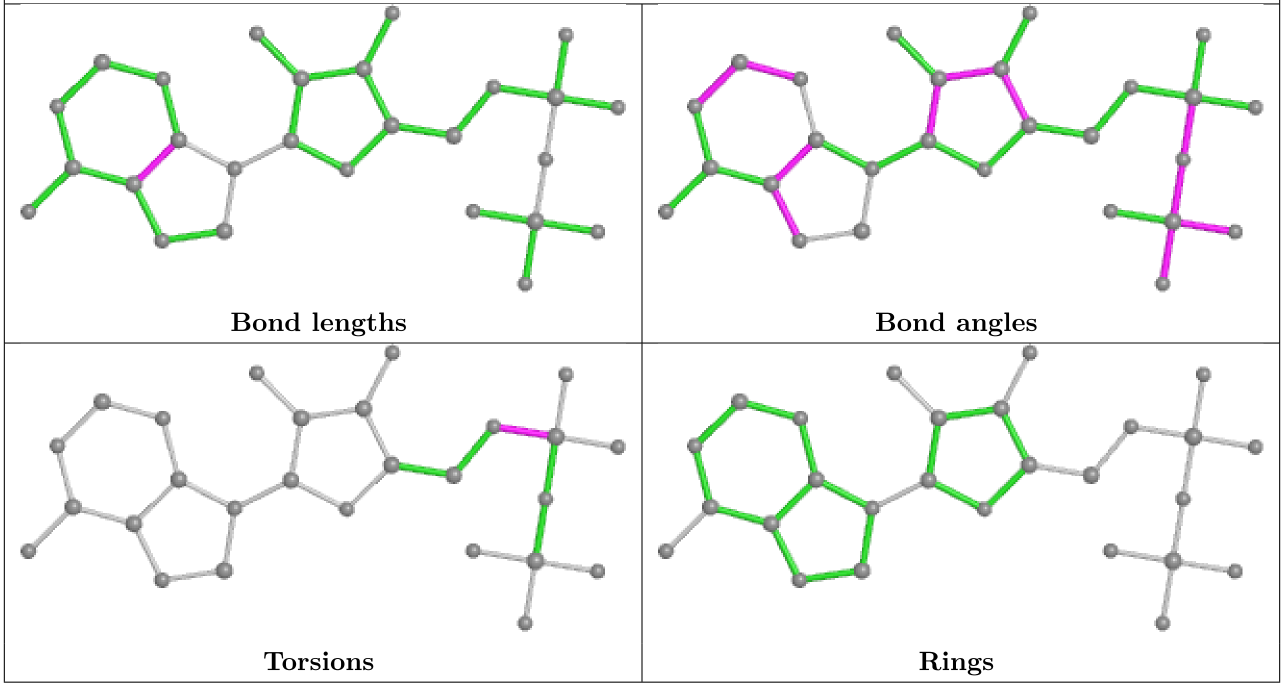

Ligand ADP D 402

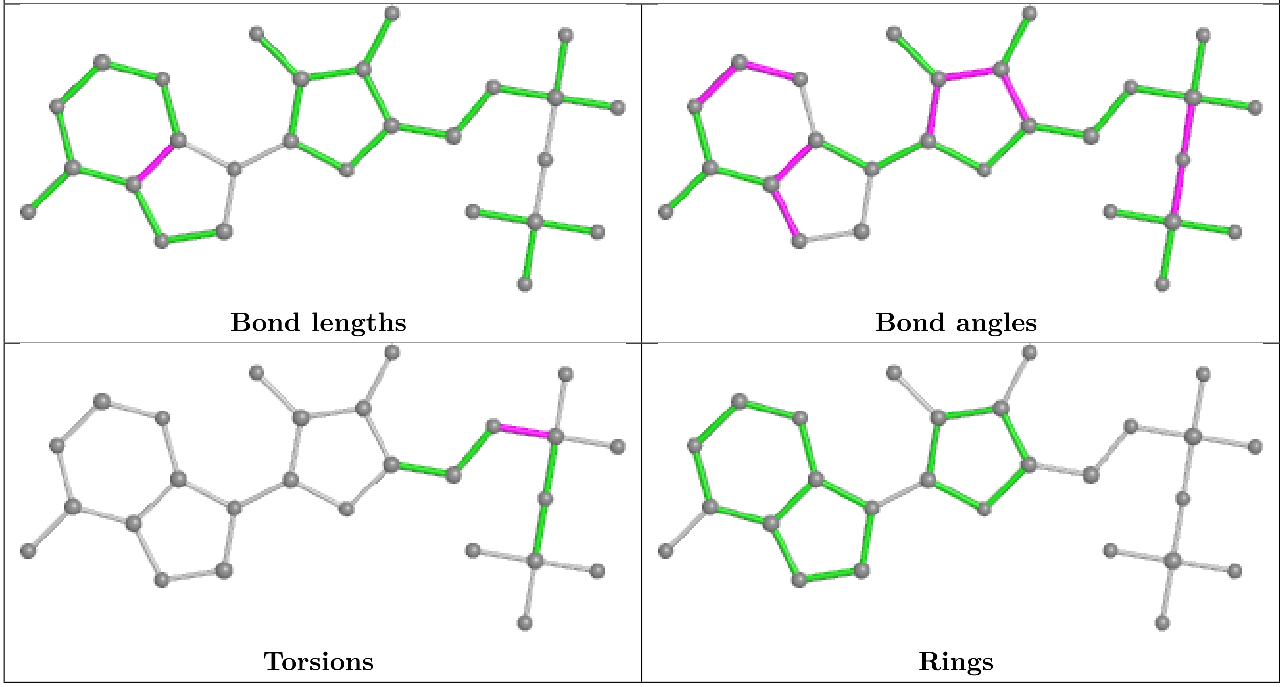

Ligand ADP A 402

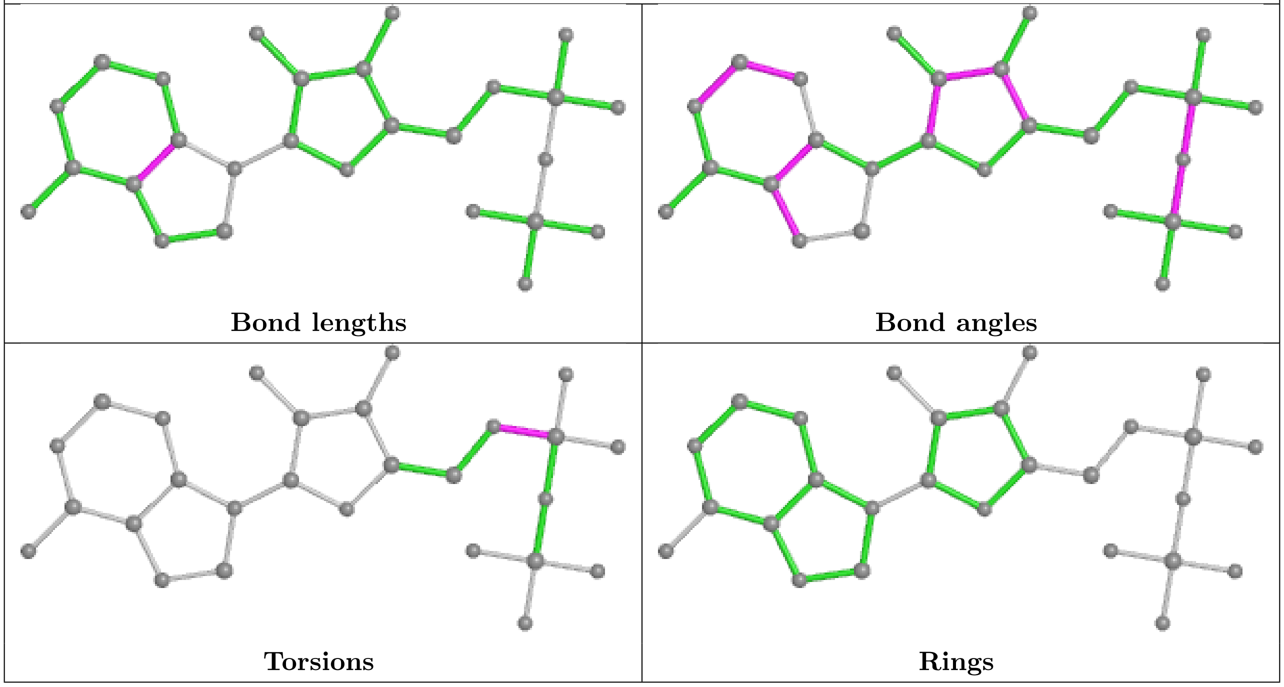

5.7 Other polymers (i)

There are no such residues in this entry.

5.8 Polymer linkage issues (i)

There are no chain breaks in this entry.

6 Map visualisation (i)

This section contains visualisations of the EMDB entry EMD-44153. These allow visual inspection of the internal detail of the map and identification of artifacts.

Images derived from a raw map, generated by summing the deposited half-maps, are presented below the corresponding image components of the primary map to allow further visual inspection and comparison with those of the primary map.

6.1 Orthogonal projections (i)

6.1.1 Primary map

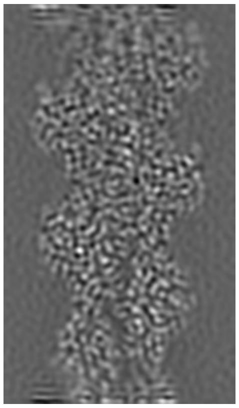
X

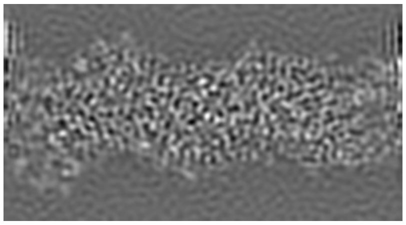
Y

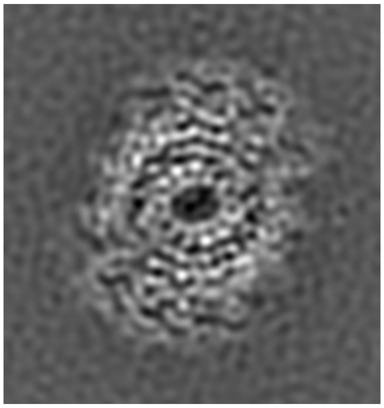
Z

6.1.2 Raw map

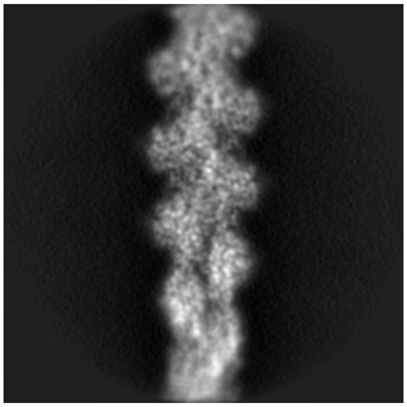
X

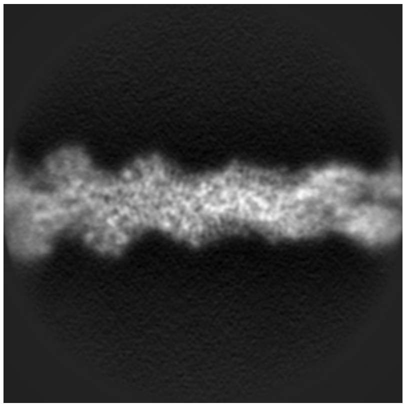
Y

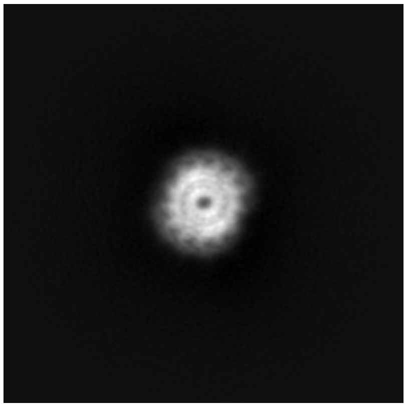
Z

The images above show the map projected in three orthogonal directions.

6.2 Central slices (i)

6.2.1 Primary map

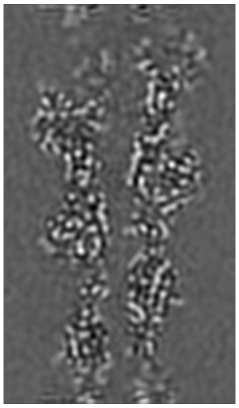

X Index: 47

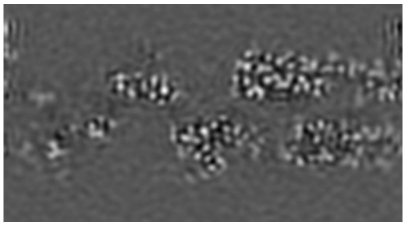

Y Index: 50

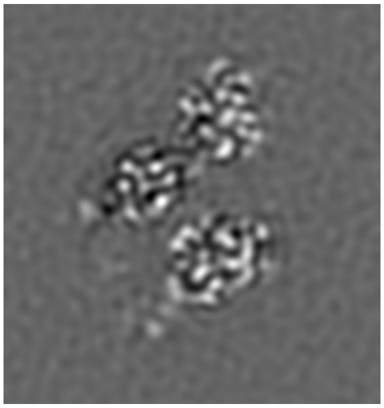

Z Index: 87

6.2.2 Raw map

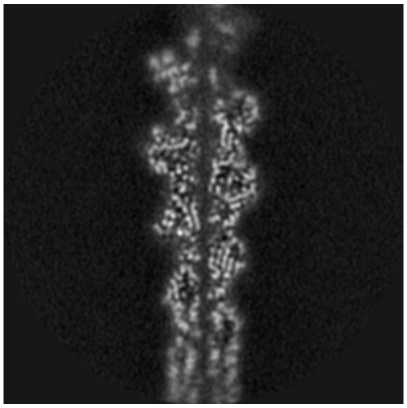

X Index: 140

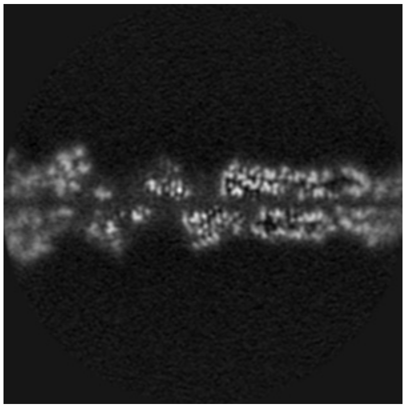

Y Index: 140

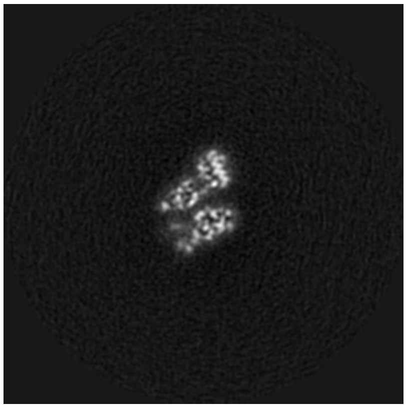

Z Index: 140

The images above show central slices of the map in three orthogonal directions.

6.3 Largest variance slices (i)

6.3.1 Primary map

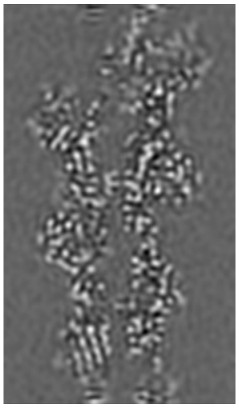

X Index: 44

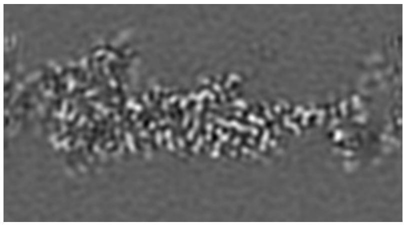

Y Index: 63

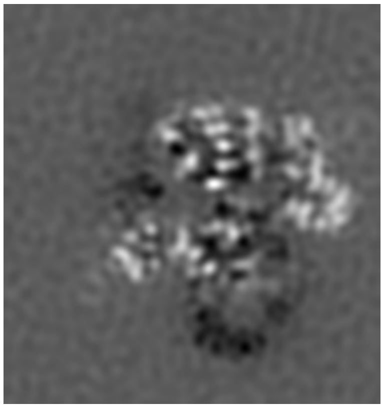

Z Index: 1

6.3.2 Raw map

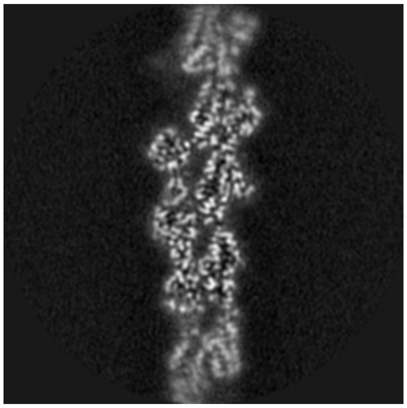

X Index: 133

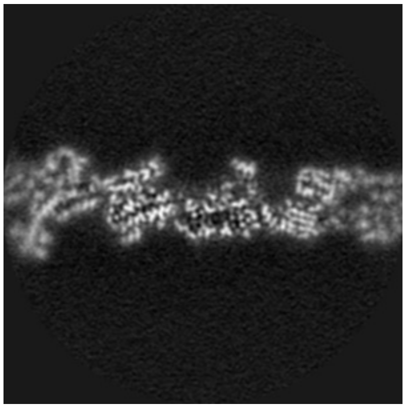

Y Index: 148

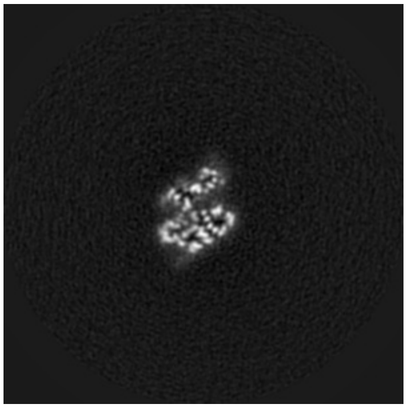

Z Index: 132

The images above show the largest variance slices of the map in three orthogonal directions.

6.4 Orthogonal standard-deviation projections (False-color) (i)

6.4.1 Primary map

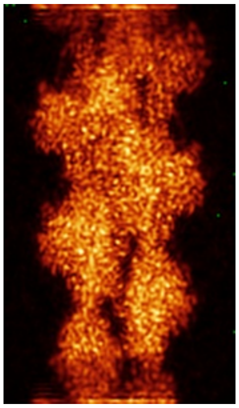
X

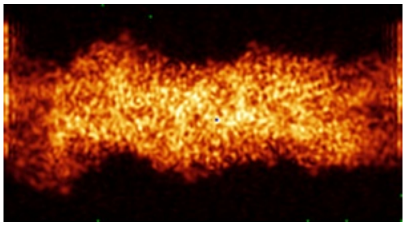
Y

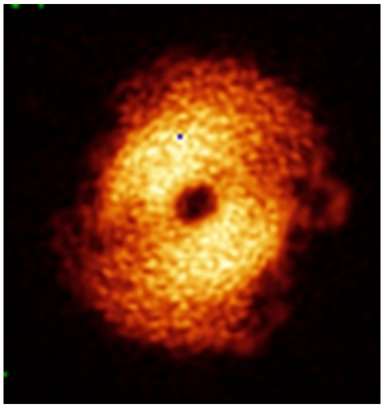
Z

6.4.2 Raw map

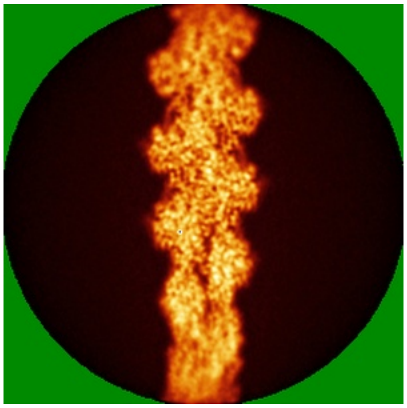
X

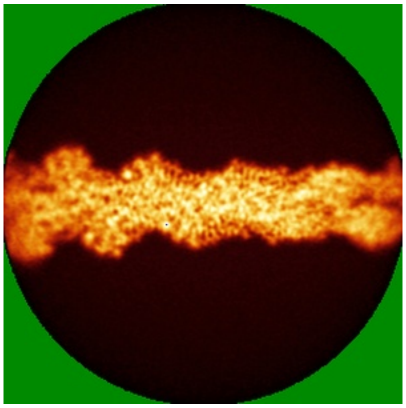
Y

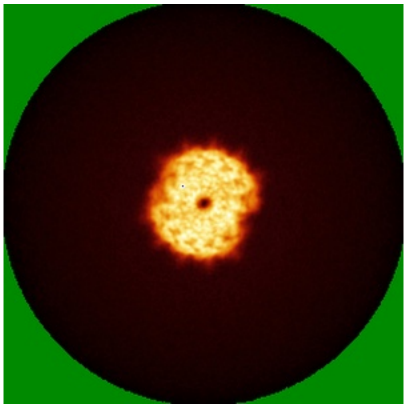
Z

The images above show the map standard deviation projections with false color in three orthogonal directions. Minimum values are shown in green, max in blue, and dark to light orange shades represent small to large values respectively.

6.5 Orthogonal surface views (i)

6.5.1 Primary map

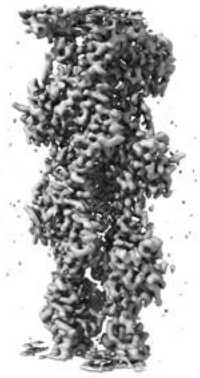

X

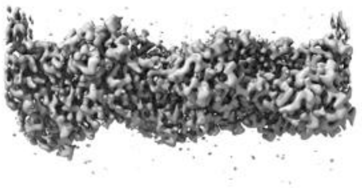

Y

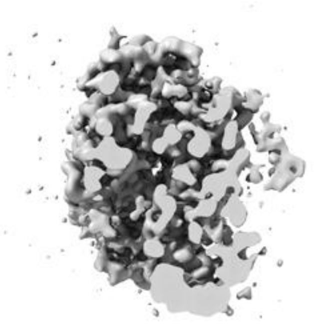

Z

The images above show the 3D surface view of the map at the recommended contour level 0.14. These images, in conjunction with the slice images, may facilitate assessment of whether an appropriate contour level has been provided.

6.5.2 Raw map

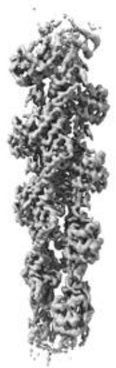

X

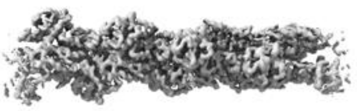

Y

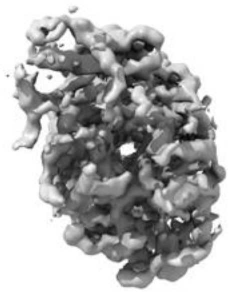

Z

These images show the 3D surface of the raw map. The raw map's contour level was selected so that its surface encloses the same volume as the primary map does at its recommended contour level.

6.6 Mask visualisation (i)

This section shows the 3D surface view of the primary map at 50% transparency overlaid with the specified mask at 0% transparency

A mask typically either:

- Encompasses the whole structure
- Separates out a domain, a functional unit, a monomer or an area of interest from a larger structure

6.6.1 emd_44153_msk_1.map (i)

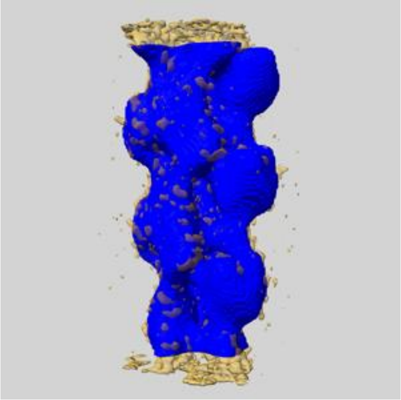

X

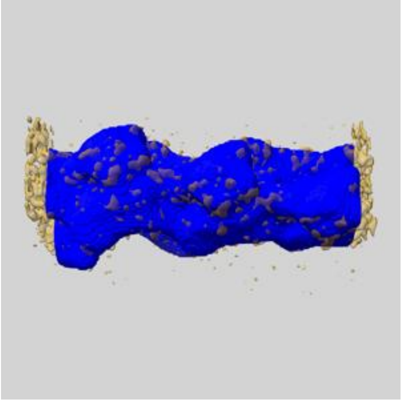

Y

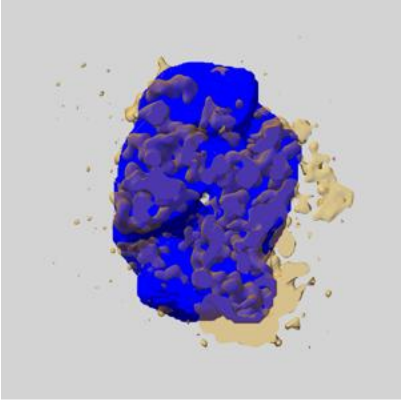

Z

7 Map analysis (i)

This section contains the results of statistical analysis of the map.

7.1 Map-value distribution (i)

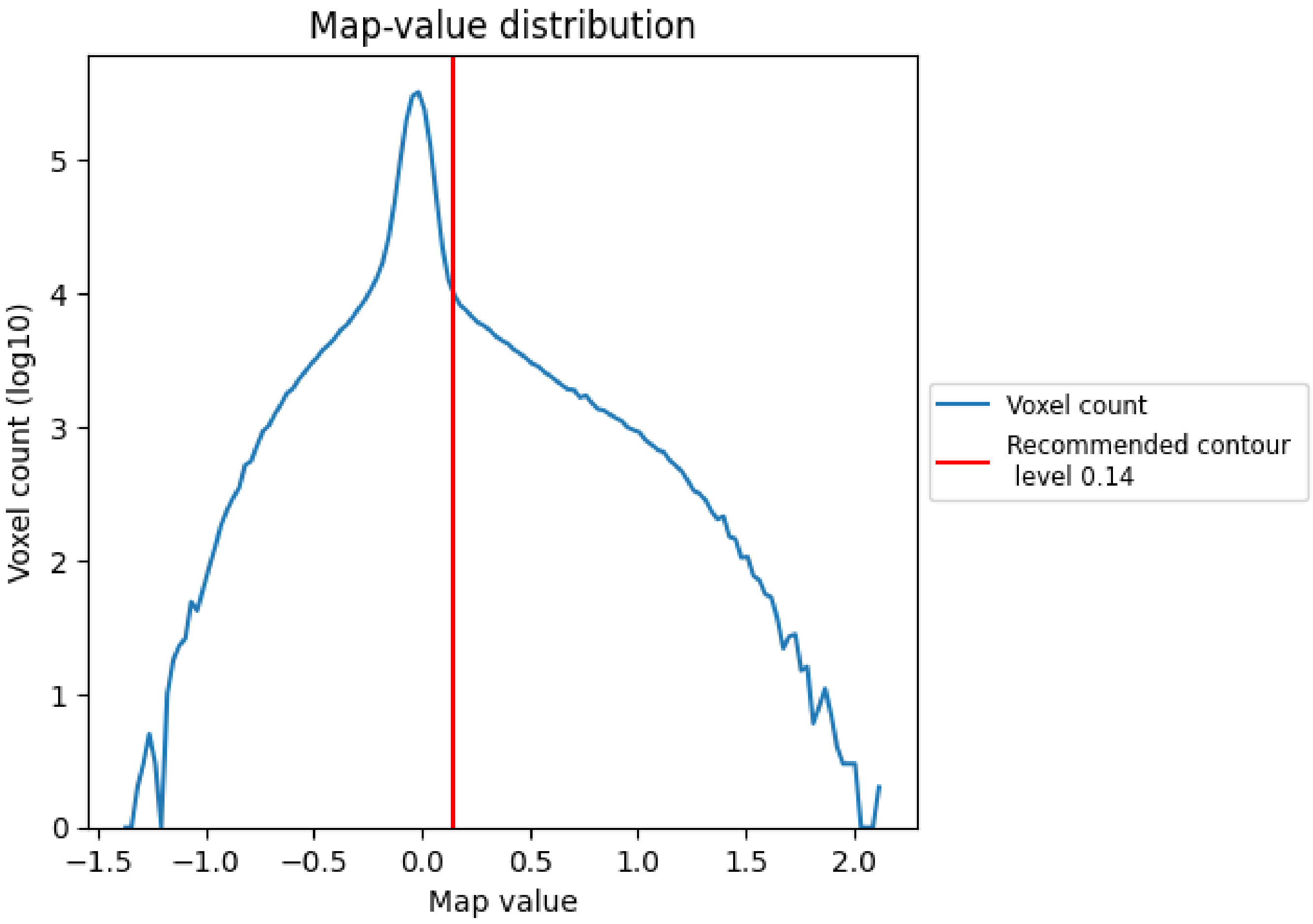

The map-value distribution is plotted in 128 intervals along the x-axis. The y-axis is logarithmic. A spike in this graph at zero usually indicates that the volume has been masked.

7.2 Volume estimate (i)

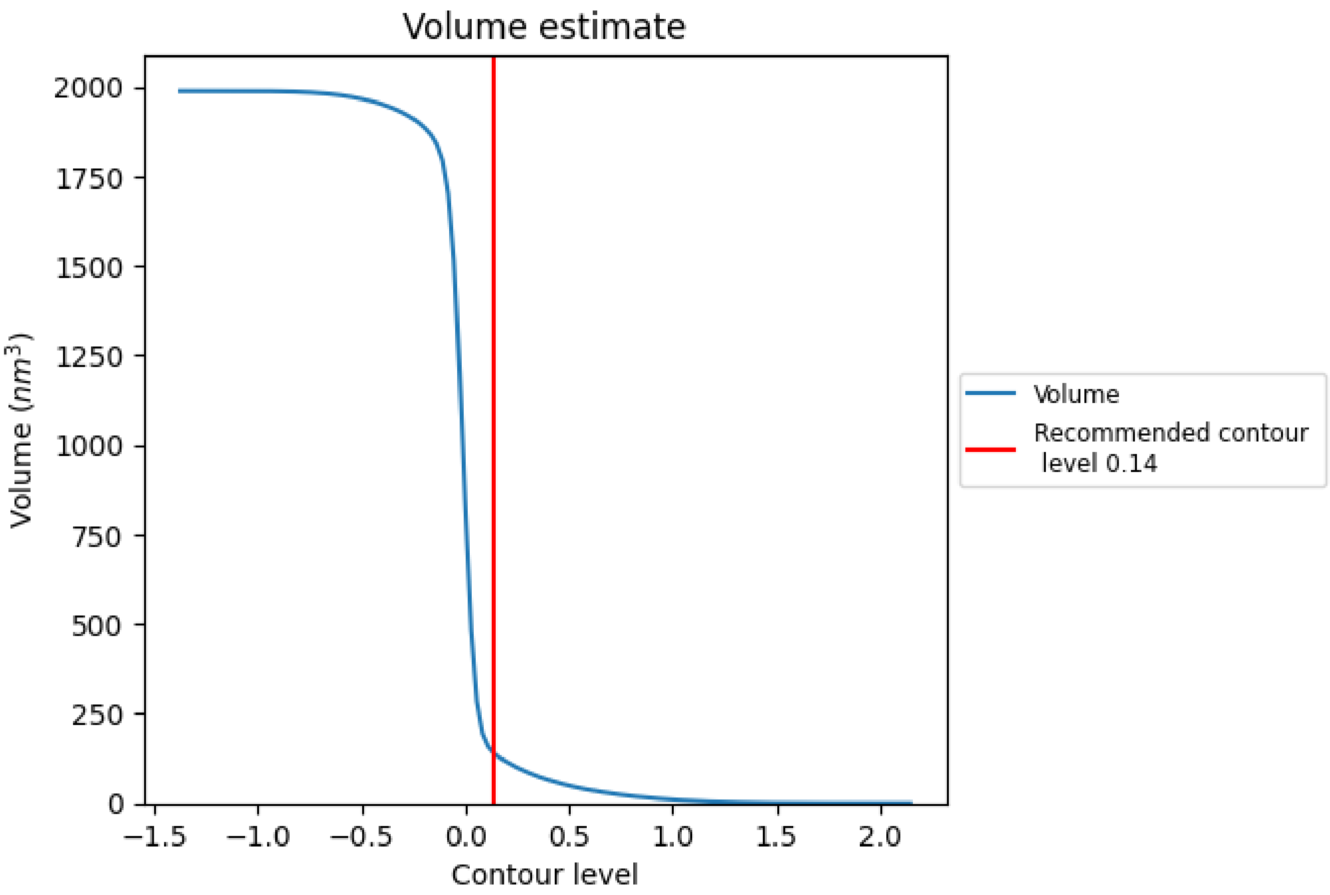

The volume at the recommended contour level is 139 nm^3; this corresponds to an approximate mass of 126 kDa.

The volume estimate graph shows how the enclosed volume varies with the contour level. The recommended contour level is shown as a vertical line and the intersection between the line and the curve gives the volume of the enclosed surface at the given level.

7.3 Rotationally averaged power spectrum (i)

This section was not generated. The rotationally averaged power spectrum is only generated for cubic maps.

8 Fourier-Shell correlation (i)

Fourier-Shell Correlation (FSC) is the most commonly used method to estimate the resolution of single-particle and subtomogram-averaged maps. The shape of the curve depends on the imposed symmetry, mask and whether or not the two 3D reconstructions used were processed from a common reference. The reported resolution is shown as a black line. A curve is displayed for the half-bit criterion in addition to lines showing the 0.143 gold standard cut-off and 0.5 cut-off.

8.1 FSC (i)

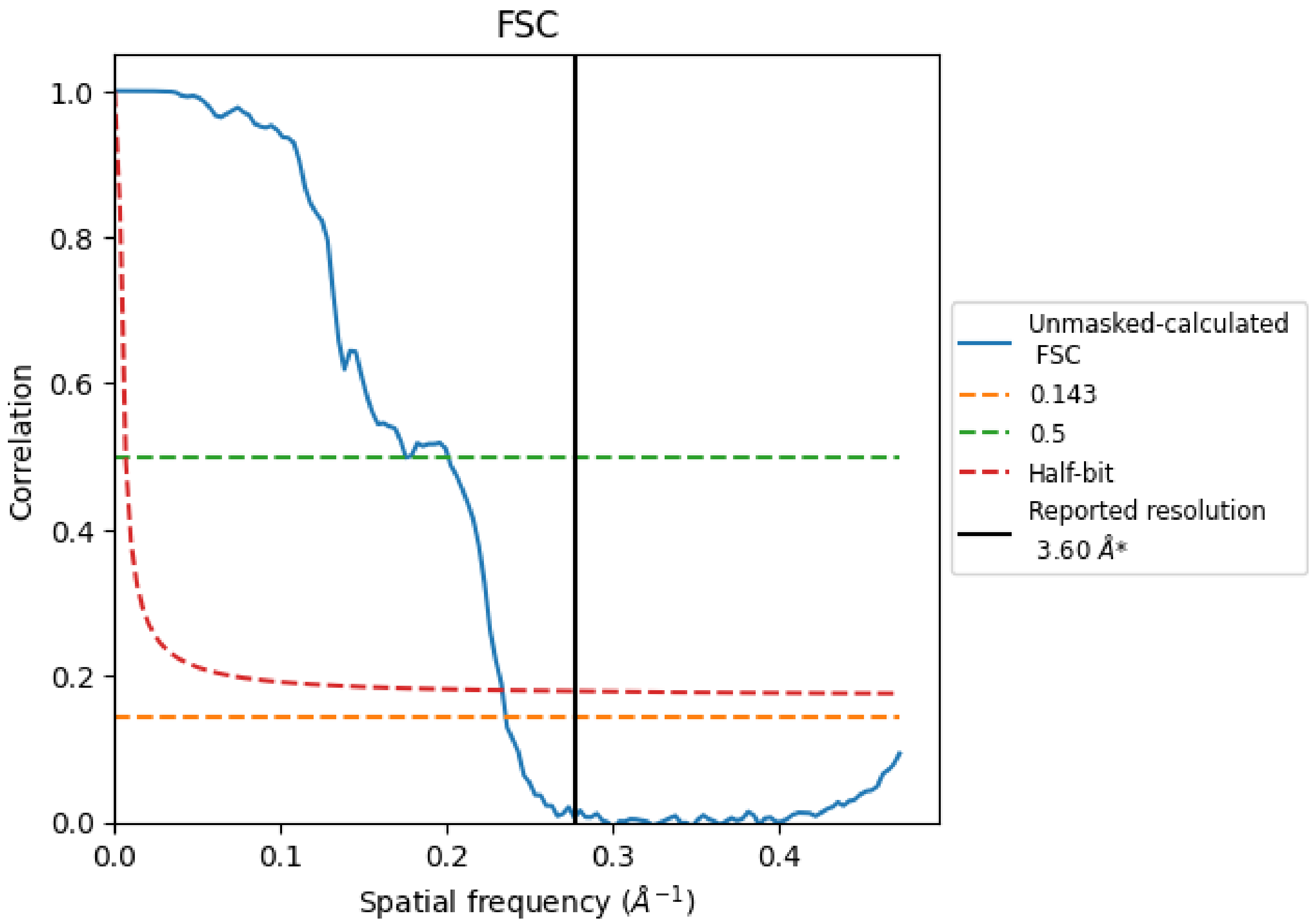

*Reported resolution corresponds to spatial frequency of 0.278 Å^{-1}

8.2 Resolution estimates (i)

Resolution estimate (Å)	Estimation criterion (FSC cut-off)		
	0.143	0.5	Half-bit
Reported by author	3.60	-	-
Author-provided FSC curve	-	-	-
Unmasked-calculated*	4.25	5.70	4.28

*Resolution estimate based on FSC curve calculated by comparison of deposited half-maps. The value from deposited half-maps intersecting FSC 0.143 CUT-OFF 4.25 differs from the reported value 3.6 by more than 10 %

9 Map-model fit

This section contains information regarding the fit between EMDB map EMD-44153 and PDB model 9B3Q. Per-residue inclusion information can be found in section 3 on page 5.

9.1 Map-model overlay

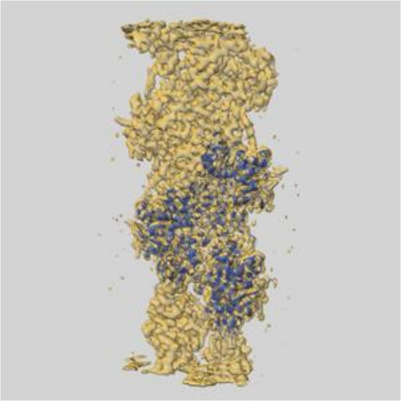
X

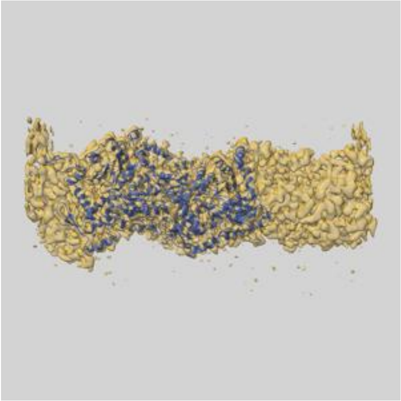
Y

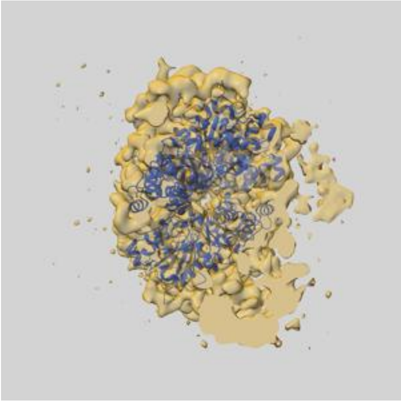
Z

The images above show the 3D surface view of the map at the recommended contour level 0.14 at 50% transparency in yellow overlaid with a ribbon representation of the model coloured in blue. These images allow for the visual assessment of the quality of fit between the atomic model and the map.

9.2 Q-score mapped to coordinate model (i)

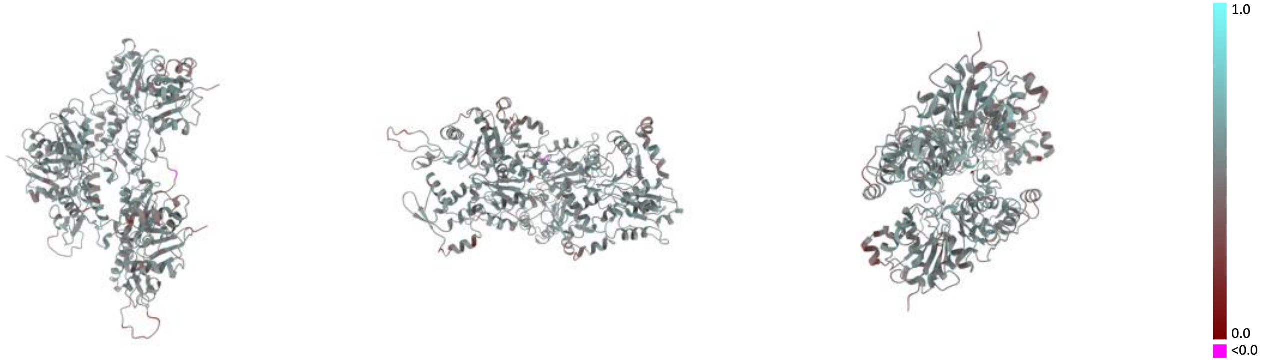

X Y Z

The images above show the model with each residue coloured according its Q-score. This shows their resolvability in the map with higher Q-score values reflecting better resolvability. Please note: Q-score is calculating the resolvability of atoms, and thus high values are only expected at resolutions at which atoms can be resolved. Low Q-score values may therefore be expected for many entries.

9.3 Atom inclusion mapped to coordinate model (i)

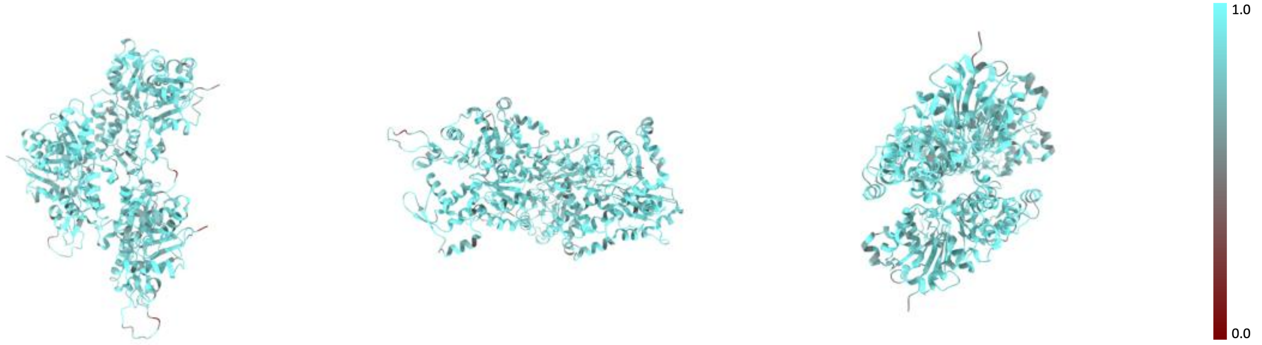

X Y Z

The images above show the model with each residue coloured according to its atom inclusion. This shows to what extent they are inside the map at the recommended contour level (0.14).

9.4 Atom inclusion (i)

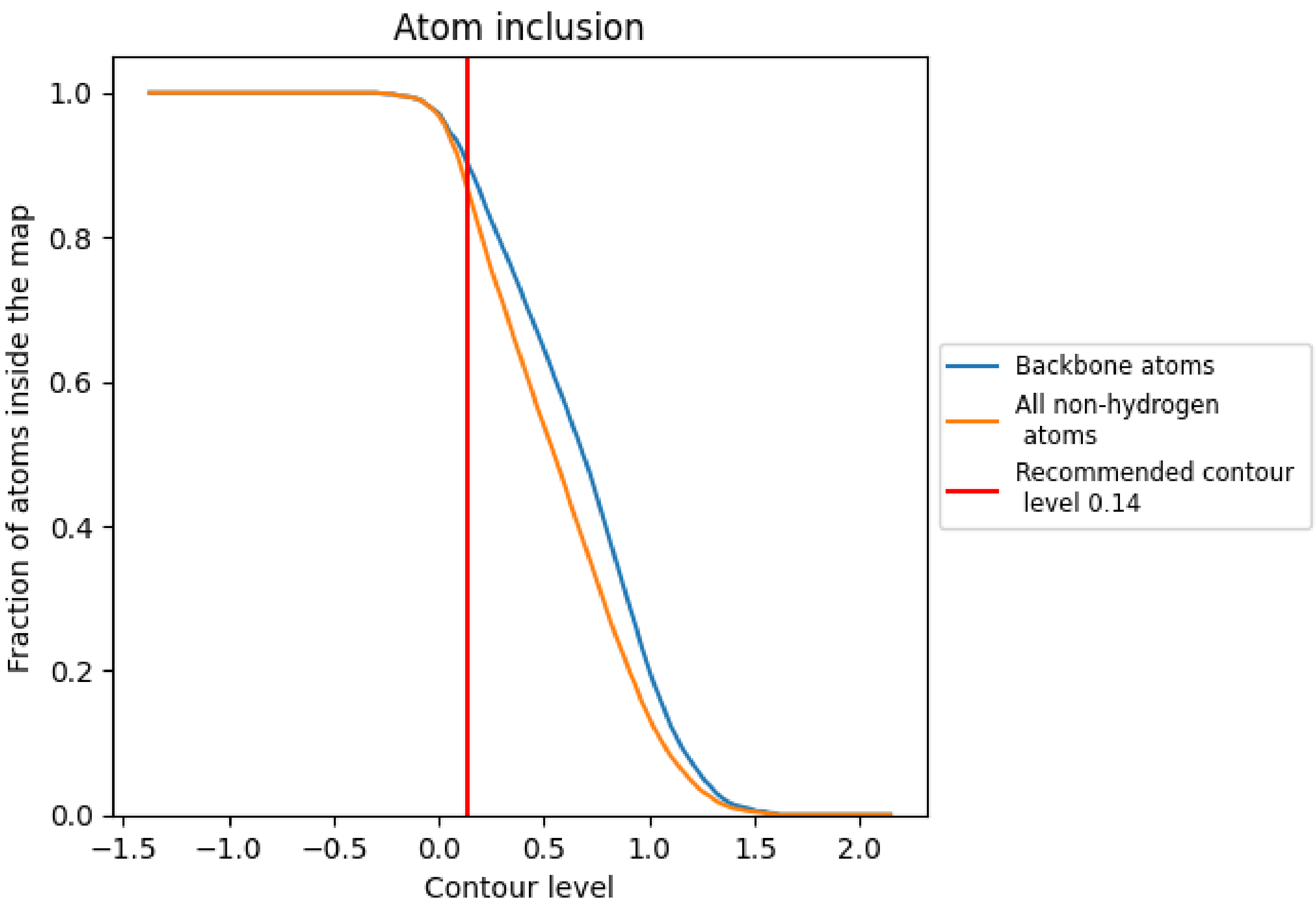

At the recommended contour level, 90% of all backbone atoms, 86% of all non-hydrogen atoms, are inside the map.

9.5 Map-model fit summary (i)

The table lists the average atom inclusion at the recommended contour level (0.14) and Q-score for the entire model and for each chain.

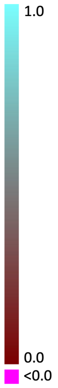

Chain	Atom inclusion	Q-score
All	0.8640	0.5130
A	0.8640	0.5190
D	0.8560	0.5070
E	0.8710	0.5130
